# Anti-PD-L1 therapy altered inflammation but not survival in a lethal murine hepatitis virus-1 pneumonia model

**DOI:** 10.3389/fimmu.2023.1308358

**Published:** 2024-01-08

**Authors:** Colleen S. Curran, Xizhong Cui, Yan Li, Mark Jeakle, Junfeng Sun, Cumhur Y. Demirkale, Samuel Minkove, Victoria Hoffmann, Rhea Dhamapurkar, Symya Chumbris, Cameron Bolyard, Akunna Iheanacho, Peter Q. Eichacker, Parizad Torabi-Parizi

**Affiliations:** ^1^ National Heart Lung and Blood Institute, National Institutes of Health, Bethesda, MD, United States; ^2^ Critical Care Medicine Department, Clinical Center, National Institutes of Health, Bethesda, MD, United States; ^3^ Division of Veterinary Resources, National Institutes of Health, Bethesda, MD, United States; ^4^ Texcell North-America, Inc., Frederick, MD, United States

**Keywords:** pneumonia, MHV-1, COVID-19, immunotherapy, PD-L1, CD66a, ACE, ACE2

## Abstract

**Introduction:**

Because prior immune checkpoint inhibitor (ICI) therapy in cancer patients presenting with COVID-19 may affect outcomes, we investigated the beta-coronavirus, murine hepatitis virus (MHV)-1, in a lethal pneumonia model in the absence (Study 1) or presence of prior programmed cell death ligand-1 (PD-L1) antibody (PD-L1mAb) treatment (Study 2).

**Methods:**

In Study 1, animals were inoculated intratracheally with MHV-1 or vehicle and evaluated at day 2, 5, and 10 after infection. In Study 2, uninfected or MHV-1-infected animals were pretreated intraperitoneally with control or PD-L1-blocking antibodies (PD-L1mAb) and evaluated at day 2 and 5 after infection. Each study examined survival, physiologic and histologic parameters, viral titers, lung immunophenotypes, and mediator production.

**Results:**

Study 1 results recapitulated the pathogenesis of COVID-19 and revealed increased cell surface expression of checkpoint molecules (PD-L1, PD-1), higher expression of the immune activation marker angiotensin converting enzyme (ACE), but reduced detection of the MHV-1 receptor CD66a on immune cells in the lung, liver, and spleen. In addition to reduced detection of PD-L1 on all immune cells assayed, PD-L1 blockade was associated with increased cell surface expression of PD-1 and ACE, decreased cell surface detection of CD66a, and improved oxygen saturation despite reduced blood glucose levels and increased signs of tissue hypoxia. In the lung, PD-L1mAb promoted S100A9 but inhibited ACE2 production concomitantly with pAKT activation and reduced FOXO1 levels. PD-L1mAb promoted interferon-γ but inhibited IL-5 and granulocyte-macrophage colony-stimulating factor (GM-CSF) production, contributing to reduced bronchoalveolar lavage levels of eosinophils and neutrophils. In the liver, PD-L1mAb increased viral clearance in association with increased macrophage and lymphocyte recruitment and liver injury. PD-L1mAb increased the production of virally induced mediators of injury, angiogenesis, and neuronal activity that may play role in COVID-19 and ICI-related neurotoxicity. PD-L1mAb did not affect survival in this murine model.

**Discussion:**

In Study 1 and Study 2, ACE was upregulated and CD66a and ACE2 were downregulated by either MHV-1 or PD-L1mAb. CD66a is not only the MHV-1 receptor but also an identified immune checkpoint and a negative regulator of ACE. Crosstalk between CD66a and PD-L1 or ACE/ACE2 may provide insight into ICI therapies. These networks may also play role in the increased production of S100A9 and neurological mediators in response to MHV-1 and/or PD-L1mAb, which warrant further study. Overall, these findings support observational data suggesting that prior ICI treatment does not alter survival in patients presenting with COVID-19.

## Introduction

An ongoing question since the severe acute respiratory syndrome coronavirus 2 (SARS-CoV-2) outbreak has been whether prior immune checkpoint inhibitor (ICI) therapy impacts outcomes in cancer patients with infection (coronavirus disease 2019, COVID-19) (1). ICIs counter the immunosuppressive effects that their targeted checkpoint molecules (e.g., PD-1 and PD-L1) exert on innate and adaptive immune responses, and they are highly effective for several cancer types (2). Similar ICI-induced immunostimulatory host defense effects against viral infections are being investigated (1). In COVID-19 patients, mononuclear cell PD-L1 transcript levels (3) and CD4+ T-cell surface PD-1 protein levels (4) are increased, possibly reflective of an evolving immunosuppressive response conducive to ICI therapy. However, ICIs also induce immune-related adverse events (irAEs), including pneumonitis, and could aggravate virus-associated inflammatory injury (5). To date, observational clinical studies have provided insufficient data to determine whether ICIs impact outcomes in SARS-CoV-2-infected cancer patients (1). Therefore, since ICIs’ potent antitumor effects were first demonstrated in controlled murine experiments, we investigated the effects of ICI treatment in a murine model.

Various murine models exist to examine the effects of the beta-coronavirus SARS-CoV-2 in mice. These can include adenoviral transduction of the SARS-CoV-2 receptor, human angiotensin-converting enzyme-2 (ACE2), into different mouse strains, transgenic mice that express human ACE2 under the control of mouse ACE2 or epithelial cell-specific promoters, and the formation of mouse-adapted SARS-CoV-2 viral strains that develop murine infectivity after serial *in-vivo* passaging of SARS-CoV-2 in mice (6). These approaches increase the susceptibility of mice of various genetic backgrounds to mount an immune response to a virus that is inherently incapable of murine infection. These models also need to be contained in a biosafety level (BSL)-3 laboratory.

Murine hepatitis virus (MHV) (7), a BSL-2 beta-coronavirus characterized before the identification of common cold beta-coronaviruses (8) and the first SARS-CoV (9), is a natural murine pathogen consisting of various strains. MHV susceptibility is dependent upon the viral strain and host genetics (10) similar to the human predisposition to COVID-19 (11). Models of infection in susceptible mice are described as neurotropic [MHV-JHM:C57BL/6 mice (12)], hepatotropic [MHV-3:Balb/c mice (13)], and pneumotropic [MHV-1:A/J mice (10)]. Tropism in these models also depends on the route of administration. For example, MHV-A59 infection in C57BL/6 mice manifests acute encephalitis (14), acute pneumonia (15), or hepatitis (16) after intracranial, intranasal, or intraperitoneal inoculation, respectively.

Because MHV-1 is primarily pneumotropic, produces severe acute respiratory syndrome (SARS)-like pathology in A/J mice (17), and is a BSL-2 pathogen and, thus, an accessible model for a vast majority of researchers, we investigated the effects of MHV-1 [50 plaque-forming units (PFU), intratracheally (IT)]-challenged A/J mice pretreated with anti-PD-L1 (300 μg, IP). MHV-1 binds carcinoembryonic antigen-related cell adhesion molecule (CEACAM)-1, also known as CD66a, for viral entry and infection (17). Like the SARS-CoV-2 receptor, ACE2 (18), CD66a is expressed on epithelial, endothelial, and myeloid cells and platelets (19, 20). ACE2 is an endogenous inhibitor of its analog, ACE, in the renin–angiotensin–aldosterone system (RAAS) (18). ACE is also an immune cell activation marker (21) and tissue protein induced in CD66a-deficient mice (22), suggesting that ACE and ACE2 may be altered in an MHV-1 model.

In Study 1, we investigated whether MHV-1-induced pathology was associated with PD-L1, PD-l, ACE, and CD66a cell surface expression on lung, liver, and spleen immune cells. In Study 2, we investigated the effects of PD-L1mAb pretreatment on PD-L1, PD-l, ACE, and CD66a cell surface expression, survival, tissue MHV-1 titers, inflammatory lung injury, molecular cell signaling, and the production of novel mediators in animals challenged with either MHV-1 or its diluent control. Both studies examined aspects of MHV-1 pathogenesis not previously described and emphasized the similarity of this virus to SARS-CoV-2.

## Methods

### Animals

Female 12-week-old A/J mice (Jackson Laboratory, Bar Harbor, ME, USA) weighing 20–25 g were maintained under pathogen-free conditions for Study 1 (374 mice) and Study 2 (620 mice). All studies were approved by the NIH Clinical Center Animal Care and Use Committee and carried out in accordance with NIH Animal Care and Use guidelines.

### Virus preparation

MHV-1 (Parkes strain, ATCC, Manassas, VA, USA) was propagated in NCTC Clone 1469 mouse epithelial cells (Texcell-North America, Frederick, MD, USA) to generate a viral solution with 4.9 × 10^4^ PFU/ml and stored (−80°C) until use. The epithelial cell supernatant was determined free of *Mycoplasma* and endotoxin (Texcell) and was used as a diluent-control challenge during experimentations.

### Mouse viral pneumonia model and measurements

After anesthesia with 3%–5% inhalational isoflurane, mice were intratracheally (IT) inoculated with 50 µl of MHV-1 or diluent control. IT instillation was performed with a 24-gauge IV catheter (3/4″) (Somerset, NJ, USA) after passage just beyond the vocal cords. In Study 1 and Study 2, survival was assessed in blinded treatment groups, and animals alive at 14 days were considered survivors. Viral loads in the lung and liver, body weights and temperature, oxygen saturation, complete blood cell counts, coagulation measures, lung wet-to-dry weight ratios, lung lavage cell and protein concentrations, blood and lung lavage cytokine levels, lung and liver histology assessment, and flow cytometry were performed in both Study 1 and Study 2. Olink detection of soluble proteins and immunoblots of whole lung lysates were also performed in Study 2. Survival was assessed 14 days after the challenge in animal cohorts separate from those used for timed measurements.

### Antibody treatment

Mice were administered 300 μg of isotype antibody (isomAb: BP0090, clone LTF2, Bio X Cell, West Lebanon, NH, USA) or PD-L1 monoclonal antibody (PD-L1mAb: BP0101, clone 10F.9G2, Bio X Cell) in 200 µl of sterile saline, intraperitoneally (IP), every third day beginning 12 days before and until 3 days after the MHV-1 challenge. Mice assessed at 5 days or later had received six total doses of PD-L1mAb or isomAb.

### Apportionment of laboratory measures across experiments

Because all sampling could not be done in an individual animal, experiments were devoted to performing either of the following: 1) bronchoalveolar lavage (BAL), lung wet-to-dry lung weight ratios (WDRs), tissue histology, and serum cytokines; 2) flow cytometry and coagulation parameter testing, tissue virus titers, and serum electrolyte determinations; or 3) complete blood cell count (CBC) and lung protein and RNA determinations as described in the *Results* section.

### Weight, temperature, and oxygen saturation measures

On the day of the challenge and on each subsequent experimental day, survivors were weighed. All animals prior to anesthesia for sample collection had anal temperatures (TCAT-2 temperature controller, Physitemp, Clifton, NJ, USA) and oxygen saturation (O_2_Sat), heart rate, and respiratory rate (MouseOx Plus Oximeter with pulse collar sensor, Starr Life Sciences, Oakmont, PA, USA) measured.

### Lung and liver virus titer measurements

Whole lung and partial liver specimens were collected using the aseptic technique and placed into 2 ml of 10% DMEM in sterile polypropylene tubes. Tissue samples were weighed and then homogenized for 40 s at maximal speed (Polytron PT1200E, Switzerland). The samples were stored at −80°C for titer determination (Texcell-North America, Frederick, MD, USA).

### Bronchoalveolar lavage procedure and determination of wet-to-dry lung weight ratios

After blood was drawn from the inferior vena cava, animals were immediately euthanized by cervical dislocation under isoflurane anesthesia. Using aseptic procedures, the trachea and lung were removed by cutting laterally through the rib cage. The right lung lobe was ligated with a 4-0 sterile suture, and the right upper and lower lobes were removed for wet-to-dry lung weight ratio determinations (60°C for 72 h), and the middle lobe was removed for histology studies. Bronchial alveolar lavage (BAL) was performed on the left lung lobe by injecting 4 aliquots of 0.2 ml 1× PBS into the lungs and drawing back with an 18-gauge catheter secured to the trachea. Cell count was performed on unconcentrated BAL fluid, while cytokine and protein levels were measured on the lavage supernatant after centrifugation.

### Complete blood cell count, bronchoalveolar lavage cell count and total protein analysis, and blood electrolyte measures

CBC and BAL white blood cell counts and differentials, and blood red cell, hemoglobin, and platelet concentrations were determined (Heska Element HT5 veterinary hematology analyzer, Loveland, CO, USA). Electrolytes (Na, K, CL, Ca), glucose, and lactate were measured on whole blood (STAT Profile Prime Plus Critical Care Analyzer, Nova Biomedical, Waltham, MA, USA).

### Blood coagulation and related tests

Collected blood was immediately transferred to a plastic tube with 3.2% sodium citrate in a 9:1 blood-to-sodium citrate volume ratio. Plasma was harvested after centrifugation at 12,500 rpm for 5 min. Prothrombin time (PT), activated partial thromboplastin time (aPTT), and thrombin time were tested (Start System, Stago, Mount Olive, NJ, USA). Fibrinogen, thrombin–antithrombin complex (TAT), and tissue factor pathway inhibitor (TFPI) (Abcam, Cambridge, UK) and tissue factor (TF), plasminogen activator inhibitor (PAI), tissue plasminogen activator (tPA), and D-dimer (MyBioSource, San Diego, CA, USA) were measured by ELISA based on the manufacturers’ instructions.

### Histology

The lung and liver were fixed in 10% buffered formaldehyde (24 h) and transferred to 70% ethanol. The tissue was stained with hematoxylin and eosin, and slides were prepared (Histoserv Inc., Germantown, MD, USA). Histopathology parameters were evaluated and enumerated on a 0- to 3-point scale by a histologist blinded to specimen study group assignments, with the following definitions: 0, no area affected; 1,<25% area affected; 2, 25%–75% area affected; and 3, >75% area affected. Numerically assessed parameters included severity of pneumonia score, fibrin deposition, tissue necrosis, perivascular and alveolar edema, and alveolar hemorrhage. Vascular inflammation was defined as the percentage of blood vessels affected based on the following scale: 0, none affected; 1,<25% affected; 2, 25%–75% affected; and 3, >75% affected. Thrombi are reported as the number of thrombi per ×40 high power field (HPF). Inflammatory cell infiltration and syncytia occurrence were determined to be present or not. After unblinding, the percentage of animals in a group with either of these parameters present was calculated.

### Serum and BAL detection of soluble proteins

Serum was isolated from whole blood in serum separator Microtubes (101093-958, VWR, Radnor, PA, USA). Serum protein biomarkers were determined by Olink using the Olink Target 92 Mouse Exploratory reagent kit in a Fluidigm® Biomark™ system. Serum and BAL fluid were evaluated with a Bio-Plex Pro-Mouse Group I Cytokine 23-plex assay (M60009RDPD, Bio-Rad, Hercules, CA, USA) on a Bio-Rad Bio-Plex 200 system.

### Immunoblots

Tissue was lysed in Cytobuster (Millipore 71009) and 1:33 Halt protease/phosphatase inhibitor (861281, Thermo Fisher Scientific, Roskilde, Denmark) at a volume 3× the lung weight. Each sample was mixed vigorously on a TissueLyser II (Qiagen, Germantown, MD, USA) in safe-lock tubes (022363344, Eppendorf, Enfield, CT, USA) containing one stainless steel bead 5 mm (69989, Qiagen). Lungs were mixed with 2× volume 30 s at 30 Hz, ×3 with 1 min cooling on ice between mixing. The tissue lysate was centrifuged (14,000 rpm, 5 min, 4°C) and 1× volume was added prior to mixing (30 s at 30 Hz) and centrifugation (14,000 rpm, 20 min, 4°C). The supernatant was removed and 25 μl of the supernatant was mixed with 4× loading dye (NP0007, Thermo Fisher Scientific) and 1:100 reducing agent (NP009, Thermo Fisher Scientific). Samples were sonicated, heated (99°C for 5 min), and run on 4%–12% or 12% NuPAGE gels (NP0335 or NP0341, Thermo Fisher Scientific). Proteins were transferred with a nitrocellulose Trans-Blot Turbo Transfer pack (1704159, Bio-Rad, Hercules, CA, USA) using a Bio-Rad Trans-Blot Turbo System. Blots were cut for multiple detection and incubated with antibodies (overnight, 4°C) (**Table 1**). Blots were washed in 0.5% PBST and subsequently incubated with HRP-conjugated secondary antibodies (1 h, 4°C). Bound secondary antibody was visualized following incubation of the membrane with Super Signal West chemiluminescent HRP substrate (Thermo Fisher Scientific/Pierce) and using ChemiDoc™ MP Imaging System (Bio-Rad). Luminescence was quantified and evaluated via the application of ImageJ software (National Institutes of Health).

**Table 1:** Immunoblot antibody sources and concentrations.

Immunoblot antibodies	Source	Catalog no.	Amount
ACE2	Abcam	ab108252	1:1,000
Actin	BD Biosciences	612656	1:5,000
Akt	Cell Signaling	9272s	1:1,000
Phospho-Akt (Thr308)	Cell Signaling	13038s	1:1,000
FoxO1	Cell Signaling	2880s	1:1,000
GLUT1	Cell Signaling	12939s	1:1,000
S100A8/Calgranulin A	Cell Signaling	47310	1:1,000
S100A9/Calgranulin B	Cell Signaling	sc-58706	1:200

### Cell isolation for flow cytometric analysis

Whole lung single-cell suspensions were stained as previously described (23). Anesthetized mice were intravenously administered 3 μg of CD45 antibody (APC-eFluor 780 eBioscience, see table) in 150 μl of PBS through retro-orbital injection, 3 min prior to euthanasia to identify circulating immune cell populations. Lungs were harvested *en bloc* and each lobe was perfused in a 60-mm plate with a 27-gauge needle and syringe containing a total of 3 ml of digestion mix that included 5% HI FBS (10082-147, Gibco, Grand Island, NY, USA) in RPMI (11875-093, Gibco) [media], 300 μg/ml of Liberase™ TL (5401020001, Sigma, St. Louis, MO, USA), and 10 μg/ml of DNase I (10104159001, Sigma). The lungs and digestion mix were transferred to two 1.5-ml Eppendorf tubes and disrupted with scissors. Open tubes were placed in a rack with parafilm lightly placed above the rack in an incubator for 35 min at 37°C. The lung digestion mix was then neutralized with the addition of 60 μl of EDTA (pH 8) for 5 min at 37°C and passed through a 21-gauge needle. The digestion mixture was then passed through a 100-μM mesh strainer (352360, Falcon/Corning, Salt Lake City, UT, USA) with 9 ml of media. Any tissue not fully digested was gently passed through the mesh with the head of a sterile 3-ml syringe plunger (309657, BD Bioscience, Franklin Lakes, NJ, USA) and further washed through the mesh with media. The 9-ml volume was equally distributed into three polypropylene round-bottom tubes (352063, BD Falcon, Durham, NC, USA) that were centrifuged (400×*g*/1,400 rpm, 12 min, 4°C) and the supernatants were aspirated. To increase the isolation of immune cells, cell pellets were subjected to a Percoll gradient. The pellets from the three tubes were combined into a 15-ml tube containing 10 ml of 56% media and 44% Percoll (17-0891-01, GE Healthcare, Pittsburgh, PA, USA) mixed with 1% 10× PBS to form an isotonic solution. The suspensions were centrifuged (800×*g*/2,000 rpm, 30 min, 4°C) and the suspended solution was removed with a hand pipette and replaced with 7 ml of media. The pellet suspended in 7 ml of media was centrifuged (450×*g*/1,500 rpm, 5 min, 4°C). The supernatant was aspirated, and the red blood cells were lysed in 1 ml of lysis buffer (Buffer EL, Qiagen, Germantown, MD, USA) for 3 min at RT. The reaction was quenched with 1 ml of media and the suspension was centrifuged (450×*g*/1,500 rpm, 5m, 4°C) prior to counting and distributing cells (1 × 10^6^ cells/well) to a 96-well plate (163320, Thermo Fisher Scientific, Roskilde, Denmark).

Livers were disrupted with scissors in 5 ml macrotubes (470225-006, VWR, Radnor, PA, USA) with 5 ml of a digestion mix that included 5% HI FBS (10082-147, Gibco, Grand Island, NY, USA) in RPMI (11875-093, Gibco) [media], 30 μg/ml of Liberase™ TL (5401020001, Sigma, St. Louis, MO, USA), and 20 of μg/ml DNase I (10104159001, Sigma). The tubes of the digestion mix were placed in a rack with parafilm lightly placed above the open tubes for 35 min at 37°C. The liver digestion mix was then neutralized with 100 μl of EDTA (pH 8) for 5 min at 37°C and passed through a 21-gauge needle. The digestion mixture was then passed through a 100-μM mesh strainer (352360, Falcon/Corning, Salt Lake City, UT, USA) with 9 ml of media. Any tissue not fully digested was gently passed through the mesh with the head of a sterile 3-ml syringe plunger (309657, BD Bioscience, Franklin Lakes, NJ, USA) and further washed through the mesh with media. The 9-ml volume was equally distributed into three polypropylene round-bottom tubes (352063, BD Falcon, Durham, NC, USA) that were centrifuged (400×*g*/1,400 rpm, 12 min, 4°C), and the supernatants were aspirated. The pellets from the three tubes were combined into a 15-ml tube containing 10 ml of 56% media and 44% Percoll (17-0891-01, GE Healthcare, Pittsburgh, PA, USA) mixed with 1% 10× PBS to form an isotonic solution. The suspensions were centrifuged (800×*g*/2,000 rpm, 30 min, 4°C) and the suspended solution was removed with a hand pipette and replaced with 7 ml of media. The pellet suspended in 7 ml of media was centrifuged (450×*g*/1,500 rpm, 5 min, 4°C). The supernatant was aspirated, and the red blood cells were lysed in 1 ml of lysis buffer (Buffer EL, Qiagen, Germantown, MD, USA) for 3 min at room temperature. The reaction was quenched with 1 ml of media and the suspension was centrifuged (450×*g*/1,500 rpm, 5 min, 4°C) prior to counting and distributing cells (1 × 10^6^ cells/well) to a 96-well plate (163320, Thermo Fisher Scientific, Roskilde, Denmark).

Spleens were gently passed through a 100-μM mesh strainer (352360, Falcon/Corning, Salt Lake City, UT, USA) with the head of a sterile 3-ml syringe plunger (309657, BD Bioscience, Franklin Lakes, NJ, USA), and the mesh was washed with 5 ml of media. The cell suspension was centrifuged (450×*g*/1,500 rpm, 5 min, 4°C). The supernatant was aspirated, and the red blood cells were lysed in 1 ml of lysis buffer (Buffer EL, Qiagen, Germantown, MD, USA) for 3 min at room temperature. The reaction was quenched with 1 ml of media, and the suspension was centrifuged (450×*g*/1,500 rpm, 5 min, 4°C) prior to counting and distributing cells (1 × 10^6^ cells/well) to a 96-well plate (163320, Thermo Fisher Scientific, Roskilde, Denmark).

### Cell staining for flow cytometric analysis

Plates were centrifuged (800×*g*/2,000 rpm, 2 min, 4°C) and decanted, and cell pellets were washed with 100 μl of PBS prior to suspending cells in 100 μl of PBS containing 2.5 μg/ml of Alexa Fluor 350-labeled succinimidyl ester (A10168, Thermo Fisher/Molecular Probes, 2.5 mg/ml in DMSO) for 20 min to detect viable cells through the UV laser. At the end of 20 min, 100 μl of 1% FBS PBS (FACS buffer) was added to the plate, and the plate was centrifuged (800×*g*/2,000 rpm, 2 min, 4°C), decanted, and treated with 1 μg of anti-mouse CD16/32 (101302, BioLegend, San Diego, CA, USA) in 50μl FACS buffer for 15m at 4°C followed by the addition of a 50-μl aliquot of fluorescently labeled antibodies (see **Table 2**) in 50 μl of Brilliant Stain Buffer (563794, BD Biosciences/Horizon) for 30 min at 4°C. An additional 100 μl of FACS buffer was added to the plate prior to centrifugation (800×*g*/2,000 rpm, 2 min, 4°C).

**Table 2:** Flow cytometry antibody sources and concentrations.

Flow cytometry antibodies	Source	Catalog no.	μl/1 × 10^6^ cells
ACE/CD143 Alexa Fluor-647 (clone 230214)	R&D Systems	FAB15131R-100UG	5
Rat IgG2A Alexa Fluor-647	R&D Systems	IC006R	5
CD117 BV650 (clone 2B8)	BD Biosciences	563399	2
CD11b BV650 (clone M1/70)	BD Biosciences	563402	1
CD11c BUV737 (clone HL3)	BD Biosciences	612796	2
CD11c PE (clone HL3)	BD Biosciences	557401	1
CD19 BUV737 (clone 1D3)	BD Biosciences	612781	2
CD19 BV421 (clone 1D3)	BD Biosciences	562701	1.25
CD274 [PDL1] BV605 (clone 10F.9G2)	BioLegend	124321	2.5
Rat IgG2b, k BV605 (clone RTK530)	BioLegend	400649	2.5
CD279 [PD-1] FITC (clone 29F.1A12)	BioLegend	135214	2
Rat IgG2a, k FITC (clone RTK2758)	BioLegend	400506	2
CD3e PE (clone 145-2C11)	BD Biosciences	553063	2
CD3e PE-Cyanine7 (clone 145-2C11)	BD Biosciences	552774	2
CD38 BV650 (clone 90/CD38)	BD Biosciences	740489	2
CD4 Pacific Blue (clone RM4-5)	BioLegend	100531	1
CD4 PerCP (clone RM4-5)	BioLegend	100538	1
CD44 PE-Cyanine7 (clone IM7)	eBiosciences/Thermo Fisher	25-0441-82	1
CD45 APC-eFluor 780 (clone 30-F11)	eBiosciences/Thermo Fisher	47-0451-82	0.5
CD45 PerCP (clone 30-F11)	BioLegend	103130	1
CD62L APC (clone MEL-14)	BioLegend	104412	1
CD66a (CEACAM1a) PE (clone Mab-CC1)	BioLegend	134506	1.25
Mouse IgG1, κ PE (clone MOPC-21)	BioLegend	400112	1.25
CD8 BV711 (clone 53-6.7)	BioLegend	100759	1
Ly-6C BV421 (clone AL-21)	BD Biosciences	562727	1
Ly-6G BV711 (clone 1A8)	BD Biosciences	563979	1
Siglec-F PE-Vio770 (clone ES22-10D8)	Miltenyi	130-102-167	10

### Cell fixation for flow cytometric analysis

Cluster tubes (4401, Corning, Salt Lake City, UT, USA) were labeled and filled with 500 μl of fixation buffer containing PBS with 2% methanol-free formaldehyde (669030, Polysciences, Warrington, PA, USA). Plates were decanted and 100 μl of fixation buffer was removed from the cluster tubes and transferred to the plate with a multichannel pipette. Cells were gently mixed with the fixation buffer by pipetting until the cells were fully lifted. Cells suspended in fixation buffer were transferred to respectively labeled cluster tubes for a 20-min incubation at room temperature. An additional 500 μl of PBS was added to the tubes prior to centrifugation (450×*g*/1,500 rpm, 5 min, 4°C). The tubes were aspirated to ~50 μl and 150 μl of PBS was added to each tube.

### Flow cytometric analysis

Samples were vortexed and acquired with a BD LSRFortessa flow cytometer (BD, Franklin Lakes, NJ, USA) and analyzed with BD FlowJo data analysis software. Gating was established as detailed in **Supplementary Figure 1**. For antibodies (**Table 2**) not included in the gating (PD-L1, CD66a, ACE, PD-1), the median fluorescence intensity (MFI) was generated for the isotype controls, and these values were subtracted from the respective primary antibody MFI for each sample (*n* = 6/group over two independent experiments). These values were used in the generation of heat maps and statistical analyses.

### Determination of PD-L1 antibody dose

In our review of checkpoint inhibitor therapy in preclinical sepsis models (24), we noted that the concentrations of checkpoint inhibitors employed were between 50 and 300 μg. In our *Staphylococcus aureus* pneumonia model (23), we examined these concentrations and determined serum antibody retrieval levels of 5 μg/ml and 50 μg/ml after 54 h post-intraperitoneal administration of 50 μg and 300 μg, respectively. We chose the higher dose to provide checkpoint molecule blockade throughout the *S. aureus* study and this MHV-1 study and to more closely match dosing and serum levels reported in patients (10 mg/kg/dose) (25).

### Statistical analysis

Survival times were plotted using the Kaplan–Meier survival curves and analyzed using stratified log-rank tests. Other continuous variables were analyzed using *t*-tests for two-group comparisons and linear mixed models to account for repeated measures. Standard residual diagnostics were used to check model assumptions. For some variables, logarithm transformation was used when necessary. Categorical variables were compared using chi-square tests or Fisher exact tests. For physiologic, cell, histologic, and cytokine measures, two-way ANOVA examined the MHV-1 challenge and time effects in Study 1 and the MHV-1 challenge and PD-L1mAb treatment effects on each day in Study 2. To assess differences in the expression of immune response targets, datasets from Study 1 and Study 2 flow cytometry experiments were first log-transformed and analyzed by two-way ANOVA with repeated measures models and by *post-hoc t*-tests. These same analytic steps were used for the Olink datasets at 2 and 5 days. All *p*-values are two-sided and considered significant if *p ≤*0.05. SAS version 9.4 (Cary, NC, USA) was used for all analyses.

## Results

### Study 1

#### MHV-1-induced lung injury and physiologic changes

**Figure 1:**
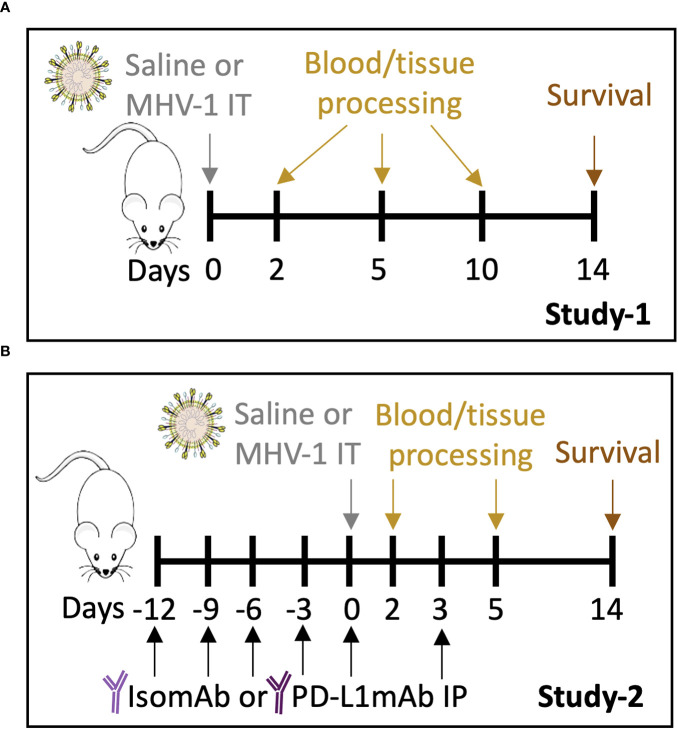
Study 1 and Study 2 protocols. **(A)** A/J mice intratracheally (IT) infected with saline vehicle control or 50 plaque-forming units (PFU)/mouse were harvested on days 2, 5, and 10 for blood and tissue processing. Survival was assessed on day 14. **(B)** A/J mice were administered intraperitoneally (IP) PD-L1mAb or isomAb every third day, starting 12 days before and continuing until 3 days after IT infection with saline vehicle control or 50 PFU/mouse. Mice were harvested on days 2 and 5 for blood and tissue processing. Survival was assessed on day 14.

**Figure 2:**
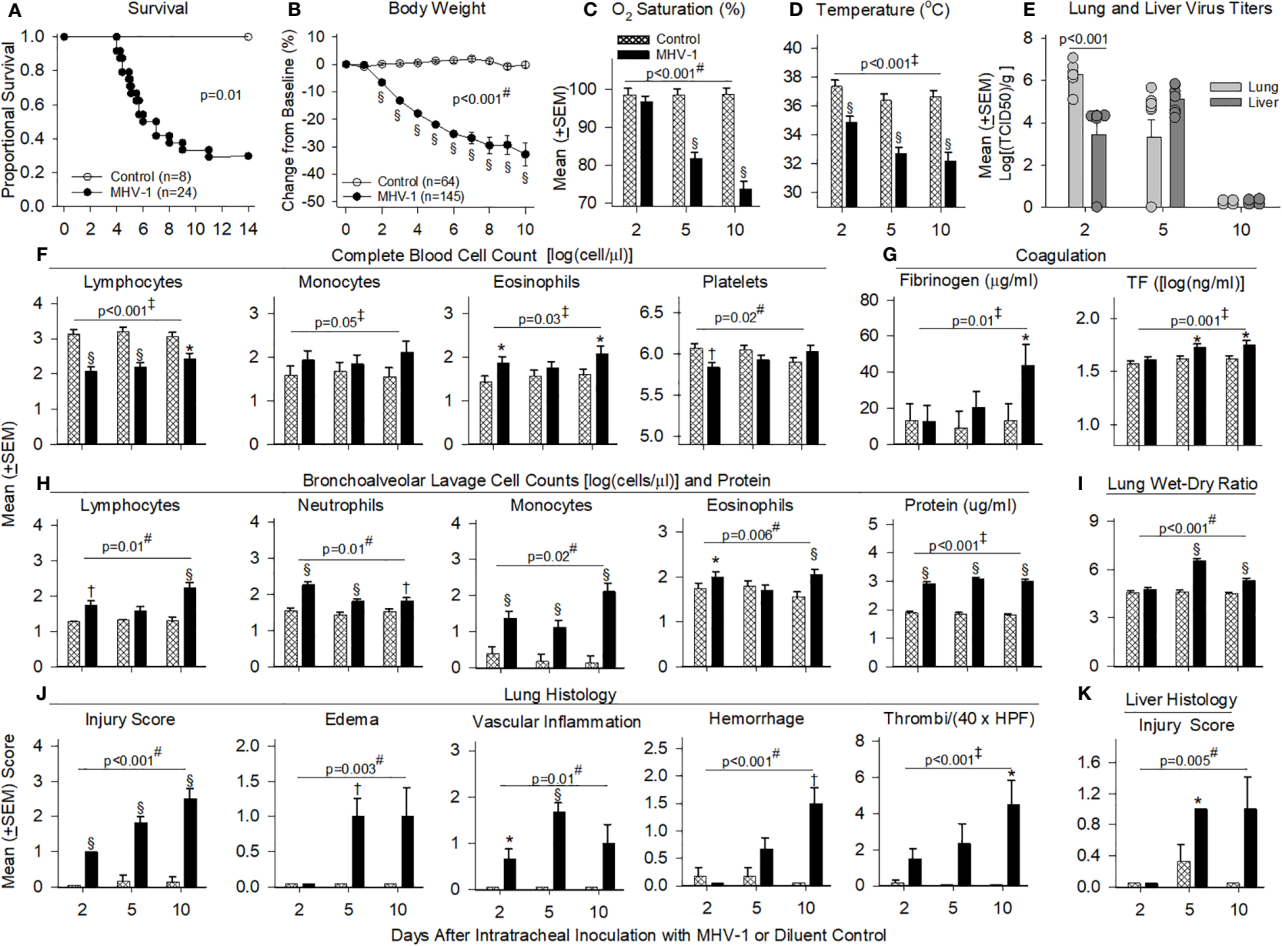
Mortality, physiology, complete blood cell counts, coagulation, bronchial alveolar lavage, and histology in animals challenged with MHV-1 compared with diluent control. **(A)** Kaplan–Meier survival curves of A/J mice infected with 50 plaque-forming units (PFU)/mouse MHV-1 compared with diluent controls. **(B)** Daily body weight measures displayed as a percent change from day 0 for diluent control and MHV-1-infected animals. **(C)** Mean O_2_ saturation from diluent control and MHV-1-infected animals (*n* = 12–24 mice/group). **(D)** Mean body temperature (°C) from diluent control and MHV-1-infected animals (*n* = 12–28 mice/group). **(E)** Viral titers [log(TCID50/g tissue)] from lung and liver of MHV-1-infected animals (*n* = 4–9 mice/group). **(F)** Complete blood cell counts for selected immune cell types [log(cell/μl)] isolated from diluent control and MHV-1-infected animals (*n* = 4–7 mice/group). **(G)** Fibrinogen (μg/ml) and tissue factor [TF, log(ng/ml)] from diluent control and MHV-1-infected animals (*n* = 5–10 mice/group). **(H)** Bronchoalveolar lavage cell count [log(cell/μl)] and protein isolated from diluent control and MHV-1-infected animals (*n* = 4–7 mice/group). **(I)** Mean lung wet-to-dry ratios from diluent control and MHV-1-infected animals (*n* = 5–7 mice/group). **(J)** Lung histology and **(K)** liver histology measurements from diluent control and MHV-1-infected animals (*n* = 4–7 mice/group). Each experimental chart represents three to four independent experiments. 0.01< *p ≤ 0.05, 0.001< †*p* ≤ 0.01, and §*p* ≤ 0.001 for MHV-1 versus control. ‡*p*-value for the overall challenge effect. #*p*-value for the challenge–time interaction. HPF, high power field; TCID50, median tissue culture infectious dose.

A dose-finding study was performed to determine the MHV-1 dose that would produce ~50% lethality for baseline assessments of the model (Study 1, **Figure 1A**) and to evaluate the beneficial and detrimental effects of interventions in this model (Study 2, **Figure 1B**). Compared with no lethality in diluent-control animals, an intratracheal MHV-1 dose of 50 PFU/mouse produced lethality, beginning at 4 days and reaching 60% by 14 days (*p* = 0.01) (**Figure 2A**, **Supplementary Figure 2**). This dose administered intratracheally is 100-fold lower than the dose delivered intranasally in another study (17), possibly due to the more targeted delivery of the virus to the lower airways. This MHV-1 dose was used in subsequent experiments. Study 1 examined MHV-1 effects (challenge effect) at day 2, 5, and 10 and whether the challenge effect differed over time (challenge–time interaction). In the absence of significant challenge–time interactions, the main effects of the MHV-1 challenge across all time points (overall effects) were estimated.

MHV-1 progressively decreased body weights starting at day 2 and oxygen saturation levels (O_2_Sat) starting at day 5 (*p* ≤ 0.001, **Figures 2B, C**). MHV-1 decreased body temperature and heart and respiratory rates [all days (*p* ≤ 0.001): **Figure 2D**, **Supplementary Figures 3A, B**]. In infected animals, MHV-1 titers were significantly higher in the lung than in liver tissue at day 2 but not at day 5 or 10 (**Figure 2E**).

MHV-1 decreased blood lymphocytes (all days) and platelets (day 2) but increased eosinophils (day 2 and 10) and monocytes (overall effect, *p* ≤ 0.05) (**Figure 2F**). Neutrophils were not significantly altered (**Supplementary Figure 3C**). MHV-1 increased fibrinogen (day 10) and tissue factor (day 5 and 10) (*p* ≤ 0.05, **Figure 2G**) but did not alter D-dimer, thrombin–antithrombin or tissue factor pathway inhibitor levels significantly (**Supplementary Figure 3D**). MHV-1 increased BAL immune cell counts (all days: *p* ≤ 0.01 except for lymphocytes and eosinophils at day 5, **Figure 2H**) as reflected in increased BAL protein (all days) and edema identified in lung wet-to-dry weight ratios (W/D: day 5 and 10) (*p* ≤ 0.001, **Figures 2H,I**).

On histology, MHV-1 progressively increased the proportion of lung tissue showing injury (all days), lung edema and vascular inflammation (day 5), hemorrhage (day 10), and thrombi formation (day 10) (*p* ≤ 0.05, **Figure 2J**). MHV-1 also induced lung necrosis (day 2 and 5), fibrin deposition (day 10), and the recruitment of lung macrophages (all days), neutrophils (day 2 and 5), and lymphocytes (day 5 and 10) (*p* ≤ 0.05, **Supplementary Figure 3E**). Taken together, signs of thrombosis, fibrin deposition, and hemorrhage suggest endothelial/vascular damage, secondary to inflammation. In the liver, MHV-1 similarly induced injury (day 5, *p* ≤ 0.05, **Figure 2K**) and necrosis (day 2 and 5) and recruited macrophages (all days), neutrophils (day 2 and 5), and lymphocytes (day 10) (*p* ≤ 0.05, **Supplementary Figure 3F**).

#### Effects of MHV-1 on serum and BAL cytokines and chemokines

**Figure 3:**
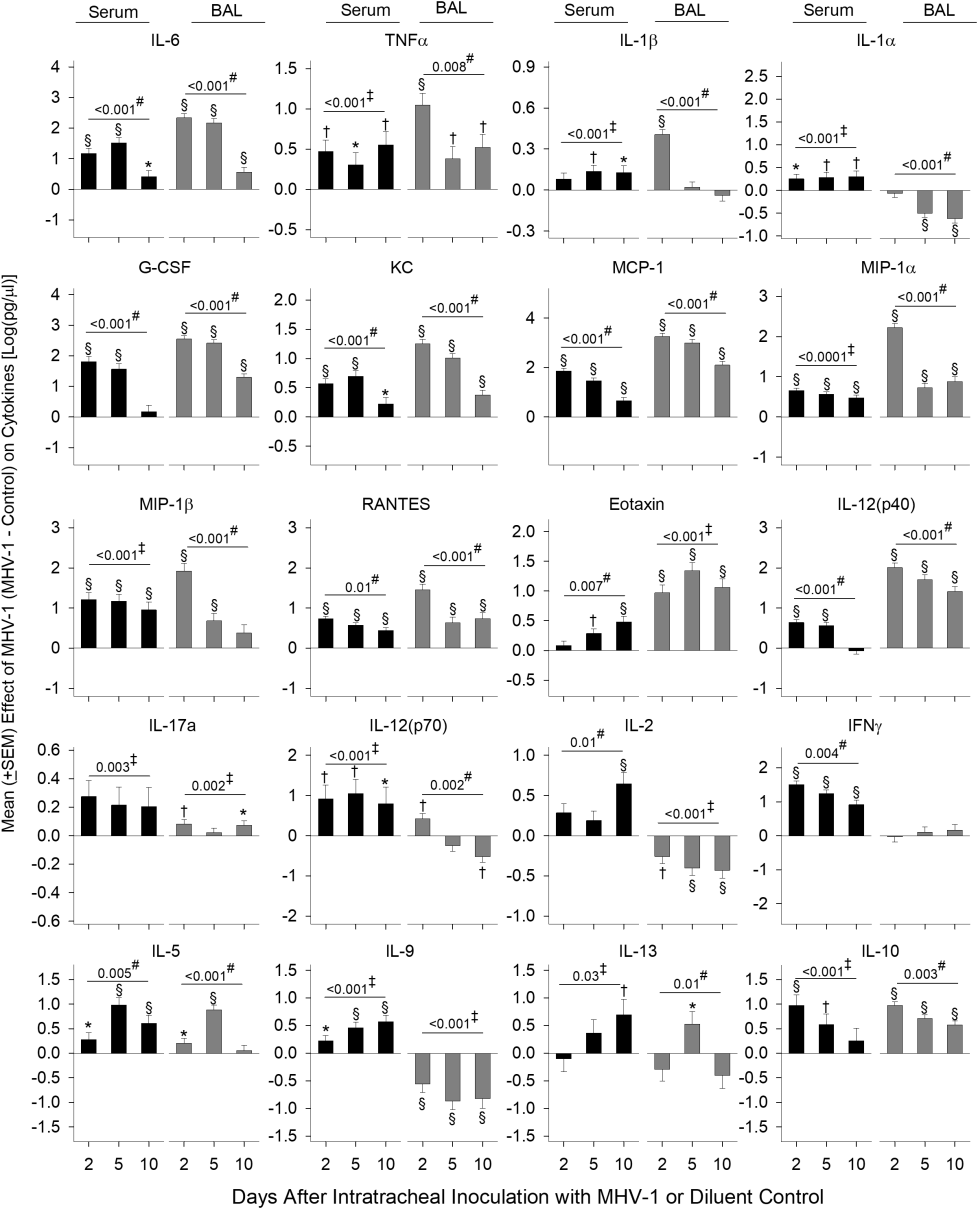
Serum and BAL mediators in MHV-1-challenged animals. Serum and BAL examined for mediators [log(pg/ml)] via a Bio-Plex assay from diluent control and MHV-1-infected animals (serum, *n* = 5–10 mice/group and BAL, *n* = 4–7 mice/group) over three to four independent experiments. The effect of the MHV-1 challenge on each mediator was calculated with mean concentrations for each mediator from MHV-1-infected animals minus the control value for each time of animal sacrifice, i.e., day 2, 5, and 10, respectively. See details in the section *Statistical method*. 0.01< *p ≤ 0.05, 0.001< †*p* ≤ 0.01, and §*p* ≤ 0.001 for MHV-1 versus control. ‡*p*-value for the overall challenge effect. #*p*-value for the challenge–time interaction.

Cytokines and chemokines in the serum (26) and BAL (27) from patients with COVID-19 have been assessed separately in various studies to identify biomarkers of disease severity. In our lethal model, we assessed mediator production from both serum and BAL at day 2, 5, and 10. Log transformed data of the mean cytokine levels in MHV-1 mice minus mean cytokine levels in control mice are displayed in **Figure 3** and the raw data are displayed in **Supplementary Table 1**.

MHV-1 increased serum and BAL inflammatory mediators, IL-6 and TNF (all days, *p* ≤ 0.05), which decreased over time, except for serum TNF (**Figure 3**). MHV-1 increased serum IL-1β and IL-1α on all days (*p* ≤ 0.05 except serum IL-1β at day 2) but only increased BAL IL-1β at day 2 and decreased BAL IL-1α at day 5 and 10 (*p* ≤ 0.001).

MHV-1 generally increased chemokines mediating myeloid cell recruitment including G-CSF, KC, MCP-1, MIP-1α, MIP-1β, RANTES, and eotaxin at each time point in both serum and BAL (*p* ≤ 0.05 except for serum G-CSF at day 10, serum eotaxin at day 2, and BAL MIP-1b at day 10). Chemokine levels decreased over time (*p* ≤ 0.01) except for serum eotaxin which increased over time (*p* = 0.007). Elevated serum MIP-1β and BAL eotaxin persisted throughout the study (*p* ≤ 0.001).

IL-12(p40), a component of IL-12/IL-23 and a factor in Th1 and Th17 cell development, increased in serum (day 2 and 5) and BAL on all days (*p* ≤ 0.001) with decreasing levels over time. IL-17a was induced in serum (overall effect: *p* = 0.003) and BAL (day 2 and 10: *p* ≤ 0.05).

IL-12(p70) and IL-2 elicit Th1 responses and stimulate interferon (IFN)-γ production. MHV-1 increased serum IL-12(p70) (day 2 and 5), IL-2 (10 days), and IFN-γ (all days) (*p* ≤ 0.001). In BAL, MHV-1 increased (day 2) and decreased (day 10) IL-12(p70) along with lower levels of IL-2 at all time points (*p* ≤ 0.01). BAL IFN-γ did not change compared with controls.

MHV-1 induced the production of Th2 cytokines IL-5, IL-9, and IL-13 in the serum. In BAL, IL-5 and IL-13 also increased (day 5), but IL-9 decreased (all days) (*p* ≤ 0.05). The anti-inflammatory cytokine IL-10 increased in the serum (day 2 and 5: *p* ≤ 0.01) and BAL (all days: *p* ≤ 0.001).

Overall, MHV-1 induced the production of all mediators assessed in the serum compared with controls. In BAL, mediators also increased except for reduced or absent Th1 (IL-12p70, IL-2, IFN-γ) cytokines, the Th2 cytokine IL-9, and IL-1α/β in BAL compared with controls. In BAL from patients with COVID-19 acute respiratory distress syndrome, three primary cytokines were altered (27). These included reduced levels of IFN-γ but higher levels of IL-9 and IL-1β compared with healthy controls, further implicating these cytokines in beta-coronavirus lung disease.

#### Effect of MHV-1 on lung, liver, and spleen immune cell phenotypes

**Figure 4:**
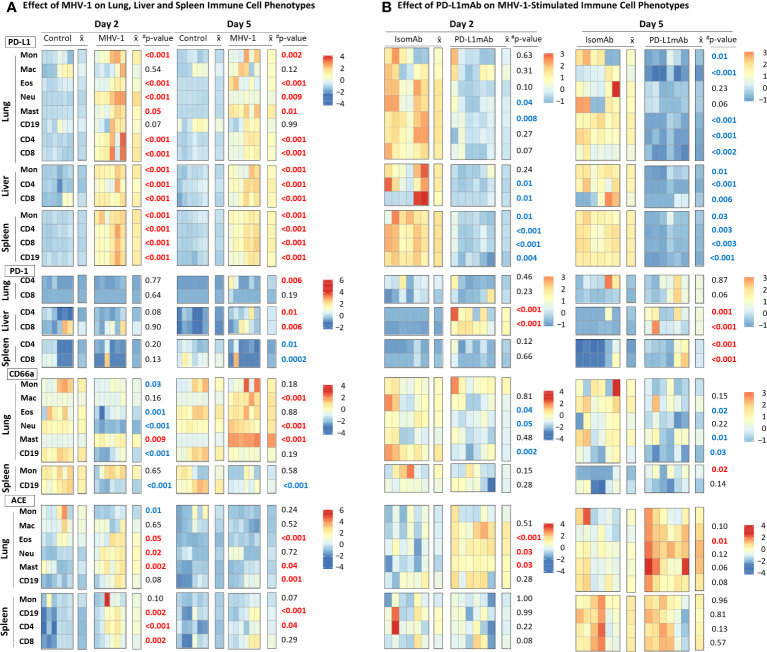
MHV-1 and PD-L1mAb induced immune cell phenotypes over time. **(A)** Lung, liver, and spleen immune cells from diluent control and MHV-1-infected animals were assessed for cell surface markers (PD-L1, PD-1, CD66a, ACE) at day 2 and 5. The median fluorescence intensities (MFIs) were obtained for each marker and the respective isotype MFIs were subtracted. Heatmaps of the control and MHV-1-challenged animals are displayed with each square representing a single animal (*n* = 6/group over two independent experiments). $\bar{\text{X}}$ represents the mean intensity for the group and time; #*p*-values comparing MHV-1 versus control. Identified markers at each timepoint increased (red) or decreased (blue), *p* ≤ 0.05. **(B)** Lung, liver, and spleen immune cells from animals pretreated with isomAb or PD-L1mAb and infected with MHV-1 were assessed for cell surface markers (PD-L1, PD-1, CD66a, ACE) at day 2 and 5. The MFIs were obtained for each marker and the respective isotype MFIs were subtracted. Heatmaps of isomAb and PD-L1mAb-treated and MHV-1-challenged animals are displayed with each square representing a single animal (*n* = 6/group over four independent experiments). $\bar{\text{X}}$ represents the mean intensity for the group and time. Identified markers at each timepoint increased (red) or decreased (blue), *p*< 0.05.

Because PD-L1 is an immunotherapeutic target in Study 2, we examined the cell surface expression of this marker and its ligand PD-1 on immune subsets in Study 1. We also monitored the expression of the MHV-1 receptor, CEACAM1/CD66a, which is an additional immunotherapeutic molecule targeted in ICI clinical trials (28). Lastly, we assessed immune cell surface expression of the activation marker, ACE (21), which may be a contributing factor to COVID-19 outcomes (29) and immunotherapeutic responses (30) in patients requiring antihypertensive treatment (e.g., ACE inhibition). The effects of the MHV-1 challenge on these markers expressed in the lung, liver, and/or spleen immune cells were assessed by flow cytometry, and the fluorescence intensities were charted by heatmap (**Figure 4A**, **Supplementary Figure 4**).

MHV-1 stimulated PD-L1 expression in almost all cells studied. MHV-1 increased PD-L1 early (day 2 and 5) on all subsets (except for lung macrophages and B cells) (*p* ≤ 0.05) and persisted at day 10 on lung eosinophils, neutrophils, and mast cells and on spleen monocytes and CD4, CD8, and CD19 cells (*p* ≤ 0.02). MHV-1-induced PD-L1 expression decreased over time (*p* ≤ 0.03) on all cell types evaluated except lung macrophages, eosinophils, mast cells, and CD19 cells.

MHV-1-induced PD-1 expression occurred late on the lung (CD4: day 5; CD8: day 10) and liver (CD4: day 5 and 10; CD8: day 5) T cells (*p* ≤ 0.01). In the spleen, MHV-1 decreased PD-1 on CD4 and CD8 cells at day 5, but the levels increased on CD4 cells at day 10 (*p* ≤ 0.03).

CD66a significantly changed over time in the lung and on spleen CD19 cells. MHV-1 decreased CD66a on lung monocytes and CD19 cells (day 2 and 10), eosinophils (day 2), and neutrophils (day 2) and on spleen CD19 cells (all days) but increased CD66a on lung macrophages (day 5 and 10), neutrophils (day 5 and 10), and mast cells (all days) (*p* ≤ 0.01). MHV-1 had no significant effect on the spleen monocyte CD66a.

MHV-1 increased ACE on lung eosinophils, neutrophils, and mast and CD19 cells and on spleen CD19, CD4, and CD8 cells at day 2 and/or day 5 but on none of these cells at day 10 (*p* ≤ 0.05). MHV-1 decreased ACE on lung monocytes at day 2 and 10 (*p* ≤ 0.03) but had no significant effects on spleen monocytes. ACE was not assessed on liver cells.

### Study 2

Animals were administered IP PD-L1mAb or isomAb every third day, starting 12 days before and continuing until 3 days after IT MHV-1 or diluent challenge, a regimen similar to those effective in murine tumor models (31, 32) (**Figure 1B**). Study 2 examined 2- and 5-day time points after infection for MHV-1 effects (challenge effect), PD-L1mAb therapy on each day (treatment effect), and whether the challenge effect differed in response to PD-L1mAb therapy (challenge–treatment interaction). In the absence of significant challenge–treatment interaction, the main effects of the MHV-1 challenge or PD-L1mAb treatment across all time points (overall effects) were estimated.

#### Effect of PD-L1mAb on MHV-1-stimulated immune cell phenotypes

To understand PD-L1mAb blocking effects compared with isomAb in diluent control and MHV-1-challenged mice, we examined cell surface PD-L1, PD-1, CD66a, and ACE on lung, liver, and/or spleen immune cells by flow cytometry. Examination of the effects of isomAb on immune cells of mice challenged with diluent control versus MHV-1 at day 2 (**Supplementary Figure 5**) and day 5 (**Supplementary Figure 6**) identified immune cell phenotypes consistent with Study 1, which did not include a treatment. In examining PD-L1mAb treatment in diluent control versus MHV-1 at day 2 (**Supplementary Figure 5**) and day 5 (**Supplementary Figure 6**), only 11 of 72 comparisons exhibited a challenge–treatment interaction. To further investigate the effects of PD-L1 blockade during beta-coronavirus infection, we generated heatmaps of only MHV-1-infected mice at day 2 and 5 and charted isomAb versus PD-L1mAb to assess the treatment effect (**Figure 4B**).

PD-L1mAb decreased the detection of PD-L1 on all cells studied in MHV-1 animals at either day 2 or 5 (**Figure 4B**). This decreased detection of PD-L1 occurred in response to the treatment antibody (PD-L1mAb, clone 10F.9G2) binding to the same PD-L1 epitope *in vivo* as the *ex-vivo* antibody (clone 10F.9G2), resulting in decreased PD-L1 detection by flow cytometry. These decreases were significant on both days for lung CD19 cells, liver CD4 and CD8 cells, and all splenic subsets (*p* ≤ 0.04). Mast cells were not detected in Study 2.

PD-L1mAb treatment increased PD-1 on liver CD4 and CD8 cells at day 2 and 5 and on spleen CD4 and CD8 cells at day 5 (*p* ≤ 0.001). PD-L1mAb increases in lung CD8 PD-1 at day 5 did not reach significance (*p* = 0.06).

PD-L1mAb decreased CD66a on lung macrophages, eosinophils, and CD19 cells at day 2 and lung macrophages, neutrophils, and CD19 at day 5 (*p* ≤ 0.05). Although treatment also decreased CD66a on lung monocytes, eosinophils, and CD19 cells at day 5, these effects were not significant (*p* ≤ 0.22). Only spleen monocytes showed an increase in CD66a with anti-PD-L1mab at day 5 (*p* = 0.02).

PD-L1mAb increased ACE on lung macrophages at day 2 and 5 and eosinophils and neutrophils at day 2 (*p* ≤ 0.03). PD-L1mAb also increased ACE on lung monocytes, eosinophils, neutrophils, and CD19 cells at day 5 in trends approaching significance (*p* ≤ 0.12). PD-L1mAb had variable and non-significant effects on ACE on spleen subsets.

#### Effect of PD-L1mAb on virus burden, survival, physiology, histology, and BAL cytokines

**Figure 5:**
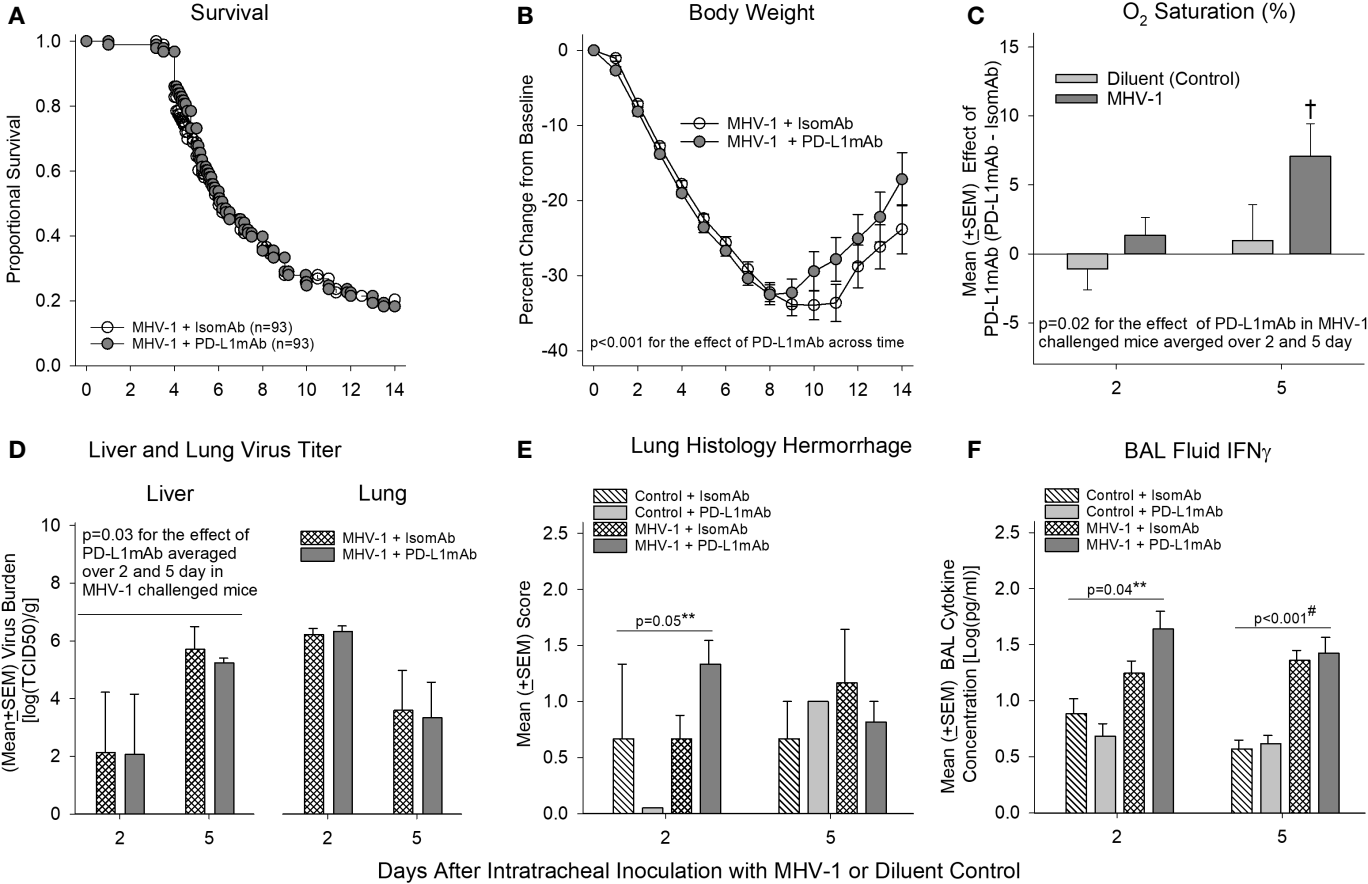
Mortality, physiology, tissue viral titers, histology, and bronchoalveolar lavage IFN-γ in animals pretreated with isomAb or PD-L1mAb and challenged with diluent or MHV-1. **(A)** Kaplan–Meier survival curves and **(B)** daily body weight measures displayed as a percent change from day 0 of A/J mice pretreated with isomAb or PD-L1mAb and challenged with 50 PFU/mouse or its diluent control (*n* = 93 at the start of the study for both groups). **(C)** O_2_ saturation for animals pretreated with isomAb or PD-L1mAb and challenged with diluent or MHV-1 (*n* = 19–33 mice/group). The effect of PD-L1mAb was calculated with the mean value from PD-L1mAb animals minus the control value for each time of animal sacrifice, i.e., 2 and 5 days, respectively. **(D)** Liver and lung viral titers (median tissue culture infectious dose, TCID50) [log(TCID50/g tissue)] from animals pretreated with isomAb or PD-L1mAb and challenged with diluent or MHV-1 (*n* = 4–6 mice/group). **(E)** Histological evaluation of lung hemorrhage from animals pretreated with isomAb or PD-L1 and challenged with diluent control or MHV-1 (*n* = 3–6 mice/group). **(F)** Bronchoalveolar lavage fluid examined by Bio-Plex for IFN-γ from animals pretreated with isomAb or PD-L1 and challenged with diluent control or MHV-1 (*n* = 5–9 mice/group). Each experimental chart represents three to four independent experiments. 0.001< †*p* ≤ 0.01 for PD-L1mAb versus isomAb within each challenge; #*p*-value for the overall challenge effect. **p-values for the challenge and treatment interaction. BAL, bronchoalveolar lavage.

Despite the highly consistent and significant effects PD-L1mAb had on reducing PD-L1 detection on immune cells and altering PD-1, CD66a, and ACE expression, PD-L1mAb treatment did not alter survival significantly in MHV-1-challenged animals (**Figure 5A**). Additionally, many of the blood, lavage, and histological parameters in the lung and liver did not exhibit a PD-L1mAb effect (**Supplementary Figures 7**, **8**).

However, some parameters did exhibit a PD-L1mAb effect. PD-L1mAb demonstrated increased weight gain in surviving infected animals (*p*< 0.001 across time: **Figure 5B**), increased O_2_ saturation (*p* = 0.02 treatment effect in MHV-1-infected animals averaged across 2 and 5 days: **Figure 5C** and **Supplementary Figure 7A**), and decreased liver MHV-1 titers (*p* ≤ 0.03 averaged over 2 and 5 days: **Figure 5D**). These beneficial PD-L1mAb responses were also associated with tissue injury. Specifically, PD-L1mAb treatment increased lung hemorrhage at day 2 (*p* = 0.05 challenge–treatment interaction: **Figure 5E**), liver injury score at day 2 (*p* = 0.02 overall treatment effect: **Supplementary Figure 8B**), fibrinogen levels at day 2 (*p* ≤ 0.05 overall treatment effect: **Supplementary Figure 7F**), and D-dimer levels at day 5 (*p* ≤ 0.04 overall treatment effect: **Supplementary Figure 7F**). These mixed PD-L1mAb responses occurred concomitantly with altered inflammation in the lung and liver.

**Figure 6:**
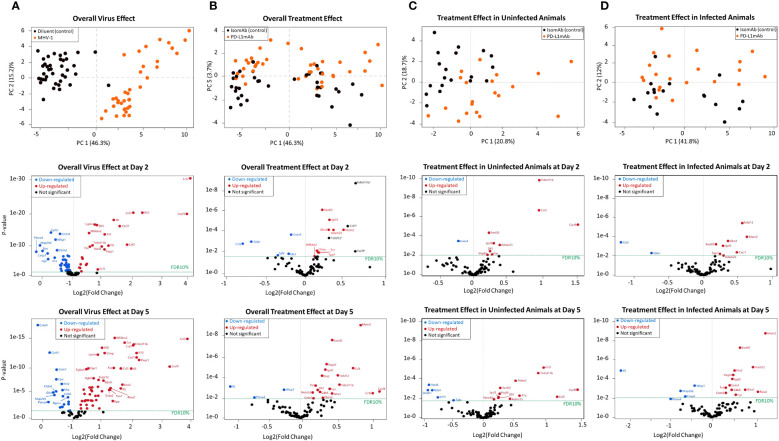
Principal component analysis and volcano plots of serum mediators detected via the Olink platform assessing the overall virus effect, overall treatment effect, treatment effect in non-infected animals, and treatment effect in infected animals. **(A)** Principal component analysis of the overall virus effect in all animals (both PD-L1 and isotype pretreated) assessed (both day 2 and 5 combined). Each dot is one animal challenged with the diluent control (black dot) or MHV-1 (orange dot). Representative volcano plots of the *p*-value versus log2(fold change) reflecting proteins that increased (red dot) or decreased (blue dot) at FDR10% in infected animals (both PD-L1 and isotype pretreated) are subsequently displayed for either day 2 or 5 overall virus effects. **(B)** Principal component analysis of the overall treatment effect in all animals (both diluent and MHV-1-challenged) assessed (both day 2 and 5 combined). Each dot is one animal pretreated with isomAb (black dot) or PD-L1mAb (orange dot). Representative volcano plots of the *p*-value versus log2(fold change) reflecting proteins that increased (red dot) or decreased (blue dot) at FDR10% in PD-L1-treated animals (both diluent- or MHV-1-challenged) are subsequently displayed for either day 2 or 5 overall treatment effects. *n* = 7–11 mice/group over three to four independent experiments. The horizontal dotted line in each volcano plot marks a *p*-value that corresponds to a 10% false discovery rate (FDR). *Proteins demonstrating an interaction effect with the virus cannot be fully explained by the treatment alone. **(C)** Principal component analysis of the treatment effect in uninfected animals, both day 2 or 5, animals combined. Each dot is one diluent-challenged animal pretreated with isomAb (black dot) or PD-L1mAb (orange dot). Representative volcano plots of the *p*-value versus log2(fold change) reflect proteins that increased (red dot) or decreased (blue dot) at FDR10% in response to treatment in uninfected animals at either day 2 or 5. **(D)** Principal component analysis of the treatment effect in animals infected with MHV-1, both day 2 and 5, animals combined. Each dot is one animal pretreated with isomAb (black dot) or PD-L1mAb (orange dot) and MHV-1-challenged. Representative volcano plots of the *p*-value versus log2(fold change) reflect proteins that increased (red dot) or decreased (blue dot) at FDR10% in response to treatment in infected animals at either 2 or 5 days. *n* = 7–11 mice/group over three to four independent experiments. The horizontal dotted line in each volcano plot marks a *p*-value that corresponds to 10% FDR.

In **Supplementary Figure 7G**, PD-L1mAb decreased the levels of BAL neutrophils at day 2 (*p* ≤ 0.05 overall treatment effect) and BAL eosinophils at day 5 (*p* ≤ 0.04 overall treatment effect), which may be associated with Csf2 [granulocyte-macrophage colony-stimulating factor (GM-CSF); (33)] and IL-5 (34) levels involved in promoting the chemotaxis and viability of neutrophils and eosinophils, respectively. In **Figure 6** and **Supplementary Table 2**, PD-L1mAb reduced the serum levels of Csf2 at day 2 (*p* = 0.012 overall treatment effect) and IL-5 at day 5 (*p* = 0.006 overall treatment effect). This reduced level of Th2 cytokines was associated with higher levels of BAL IFN-γ at day 2 in infected animals (*p* = 0.04 challenge–treatment interaction: **Figure 5F**), which was additionally induced by the virus (**Figure 3**). Additional BAL cytokines were unchanged (**Supplementary Figure 9**). In **Supplementary Figure 8B**, PD-L1mAb increased liver macrophage infiltration at day 2 (*p* = 0.02 overall treatment effect) and liver lymphocyte infiltration at day 5 (*p* = 0.001 overall treatment effect), supporting PD-L1mAb-induced viral clearance in the liver (**Figure 5D**) and increased liver injury (**Supplementary Figure 8B**).

#### Effects of MHV-1 and PD-L1mAb on serum proteins using the Olink platform

To aid in identifying novel biological processes in the MHV-1 and PD-L1mAb response, we utilized the Olink platform. This assay is based on proximity extension assay technology and assessed by PCR once a pair of oligonucleotide-labeled antibody probes bind independently to a target protein and close enough that the two oligonucleotides hybridize to form a unique identifier. The 92 murine proteins in the assay are associated with various biological processes and were assessed in the serum in our model.

When examined across serum samples from uninfected and infected animals with either PD-L1mAb or isomAb treatment, the principal component analysis showed that 46.3% of overall observed protein effects were related to MHV-1 (**Figure 6A**) and 3.7% were related to PD-L1mAb treatment (**Figure 6B**). At a false discovery rate of 10% (FDR10%), overall virus and treatment effects on serum proteins are shown in **Supplementary Table 2** and in representative volcano plots displayed in **Figures 6A, B**.

When assessing the main virus effect at day 2 and 5, 57 proteins were differentially expressed at day 2 and 75 at 5 days at FDR10% (**Figure 6A** and **Supplementary Table 2**). Eight proteins decreased at day 2 but increased at day 5. These were Ahr (aryl hydrocarbon receptor), Ca13 (carbonic anhydrase 13), Epo (erythropoietin), Igsf3 (immunoglobulin superfamily member 3), IL-1a (interleukin 1a), Tnfrsf12a [tumor necrosis factor (TNF) ligand superfamily member 12 also known as TNF-related weak inducer of apoptosis (TWEAK)], Crim1 (cysteine-rich motor neuron 1), and Tnr (tenascin-R). These shifts in virally induced serum proteins may reflect responses to hypoxia [Epo (35), IL-1a (36), Ahr (37)], changes in angiogenesis [Crim1 (38), Tnfrsf12a (39)], inflammation [Ahr (40), IL-1a (36), Tnfrsf12a (39)], or cell adhesion [Tnfrs12a and Igsf3 (41)], and additional responses not yet fully explored [Ca13 (42)].

Eight proteins were differentially expressed with respect to the overall virus effect at day 2 but not day 5 at FDR10%, and they were related to cellular growth (Hgf, hepatocyte growth factor; Tgfb1, transforming growth factor beta 1; Erbb4, human epidermal growth factor receptor 4), inflammation (IL-1b, IL-17a, and Dlk1, delta-like non-canonical notch ligand 1), adhesion (Epcam, epithelial cell adhesion molecule), and proliferation [Cant1, calcium-activated nucleotidase 1 (43)]. At day 5, 26 proteins were differentially expressed with respect to the overall virus effect that were not altered at day 2. Some of these proteins were associated with adhesion [Clmp: Coxsackie- And Adenovirus Receptor-Like Membrane Protein (44); Itgb6, integrin subunit beta 6; Itgb1bp2, integrin subunit beta 1 binding protein 2; Mia, melanoma inhibitory activity protein (45); Matn2, matrilin 2 (46)], cardiac function [Fstl3, follistatin like 3 (47); Tnni3, troponin I type 3 cardiac], and neurological function [Eno2, enolase 2/neuron-specific enolase (48); Gdnf, glial cell-derived neurotrophic factor; Ntf3, neurotrophin 3; Gfra1, glial cell-derived neurotrophic factor (GDNF) family receptor alpha 1 (49); Clmp (44); and Matn2 (46)].

The overall treatment effect at FDR10% revealed 18 differentially expressed proteins at day 2 and 29 at day 5. Of these, 12 were common to both times, including a number of upregulated neurological molecules [Adam23, ADAM metallopeptidase domain 23 (50); Sez6L2, seizure-related 6 homolog-like 2 (51); Tnr, tenascin R (52); and Matn2 (46)] (**Supplementary Table 2**, **Figure 6B**).

Subsequently, we evaluated the treatment effect in uninfected animals. Ten and 17 proteins were differentially expressed at day 2 and day 5, respectively. Of these, eight proteins were present at both times and were all increased at FDR10% (**Supplementary Table 3**, **Figure 6C**). These included chemokines [Ccl3 also known as macrophage inflammatory protein 1-alpha (MIP-1-alpha) and Cxcl9], the bone remodeling protein Tnfrsf11b (tumor necrosis factor receptor superfamily, member 11b also known as osteoprotegerin), neurological factors [Adam23 (50), Sez6L2 (seizure-related 6 homolog-like 2) (51), Igsf3 (immunoglobulin super family member 3) (53)], the notch ligand Dll1 (delta-like canonical Notch ligand 1), and Vegfd (vascular endothelial growth factor D).

However, given our interest in the effects of ICIs in the context of infection, we focused on the effects of PD-L1mAb in MHV-1-infected animals. At day 2, comparing isomAb to PD-L1mAb in infected animals at FDR10% (**Supplementary Table 3**, **Figure 6D**), PD-L1mAb downregulated serum proteins Csf2 (GM-CSF) and the intracellular enzyme, Qdpr (quinoid dihydropteridine reductase), which generates tetrahydrobiopterin (BH4) in nitric oxide production and the formation of neurotransmitters (e.g., serotonin, dopamine, norepinephrine, and epinephrine) (54). Upregulated serum proteins at day 2 were associated with neuronal activity [Adam23 (50), Sez6L2 (51), Gfra1 (49), Igsf3 (53), Matn2 (46)], lysosomal protein degradation [TPP1, tripeptidyl peptidase 1 (55)], and COVID-19 myocardial injury [Matn2 (56)] and disease severity [Tnfsf12 (57), CXCL1 (58)].

At day 5, comparing isomAb to PD-L1mAb in infected animals at FDR10% (**Supplementary Table 3**, **Figure 5D**), downregulated serum proteins are associated with apoptosis [Casp3, caspase 3 (59)], Th2/eosinophil responses [IL-5 (60)], and cellular function [Map2k6, mitogen-activated protein kinase kinase 6 (61); Wisp1, WNT1-inducible signaling pathway protein 1 (62)]. Concomitantly, increased proteins were related to COVID-19 inflammation [Tnf (58)], injury [Matn2 (56)] and severity [Tnfsf12 (57); Vegfd (63)], angiogenesis and endothelial cell growth [Vegfd (63); Flrt2, fibronectin leucine-rich transmembrane protein 2 (64)], neuronal activity [Sez6L2 (51), Gfra1 (49), Igsf3 (53), Tnr (52), and contactins (Cntn1 and Cntn4) (65)], thrombosis [cdh6, cadherin 6 (66)], and cell growth and survival [Riox2, ribosomal oxygenase 2 (67)].

#### Effects of MHV-1 and PD-L1mAb on hypoxic cell signaling

**Figure 7:**
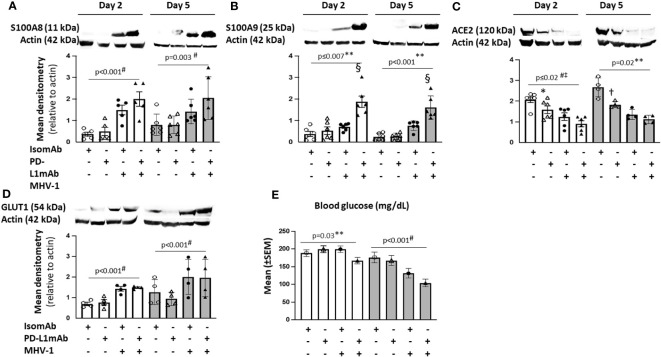
Lung cell signals in PD-L1mAb-pretreated MHV-1-challenged mice. **(A–D)** Whole lung lysates were assessed by immunoblot for S100A8 **(A)**, S100A9 dimer **(B)**, ACE2 **(C)**, or GLUT1 **(D)**. Representative images and densitometry relative to total actin are displayed for four to six animals/group over three independent experiments. **(E)** Mean blood glucose levels (mg/dl) from animals pretreated with isomAb or PD-L1 and challenged with diluent control or MHV-1 (*n* = 6–15 mice/group over three to four independent experiments). 0.01< *p ≤ 0.05, 0.001< †*p* ≤ 0.01, and §*p* ≤ 0.001 for PD‐L1mAb versus isomAb; #*p*-value for the overall challenge effect; ‡*p*-values for the overall treatment effect; **p-value for the challenge and treatment interaction.

To explore the molecular mechanisms of the PD-L1mAb response, we assessed markers associated with hypoxia and COVID-19 in our model. Hypoxia induces the production of S100A8 and S100A9 (68) studied in COVID-19 patients (69). We therefore examined hypoxic cell signals in total lung lysates. Assessing the overall MHV-1 challenge effect at day 2 and 5, MHV-1 increased S100A8, GLUT1, phosphorylated AKT (pAKT), and the pAKT to total AKT ratio but decreased ACE2 at day 2 and FOXO1 at day 2 and 5 (**Figure 7**, **Supplementary Figure 10**).

**Figure 8:**
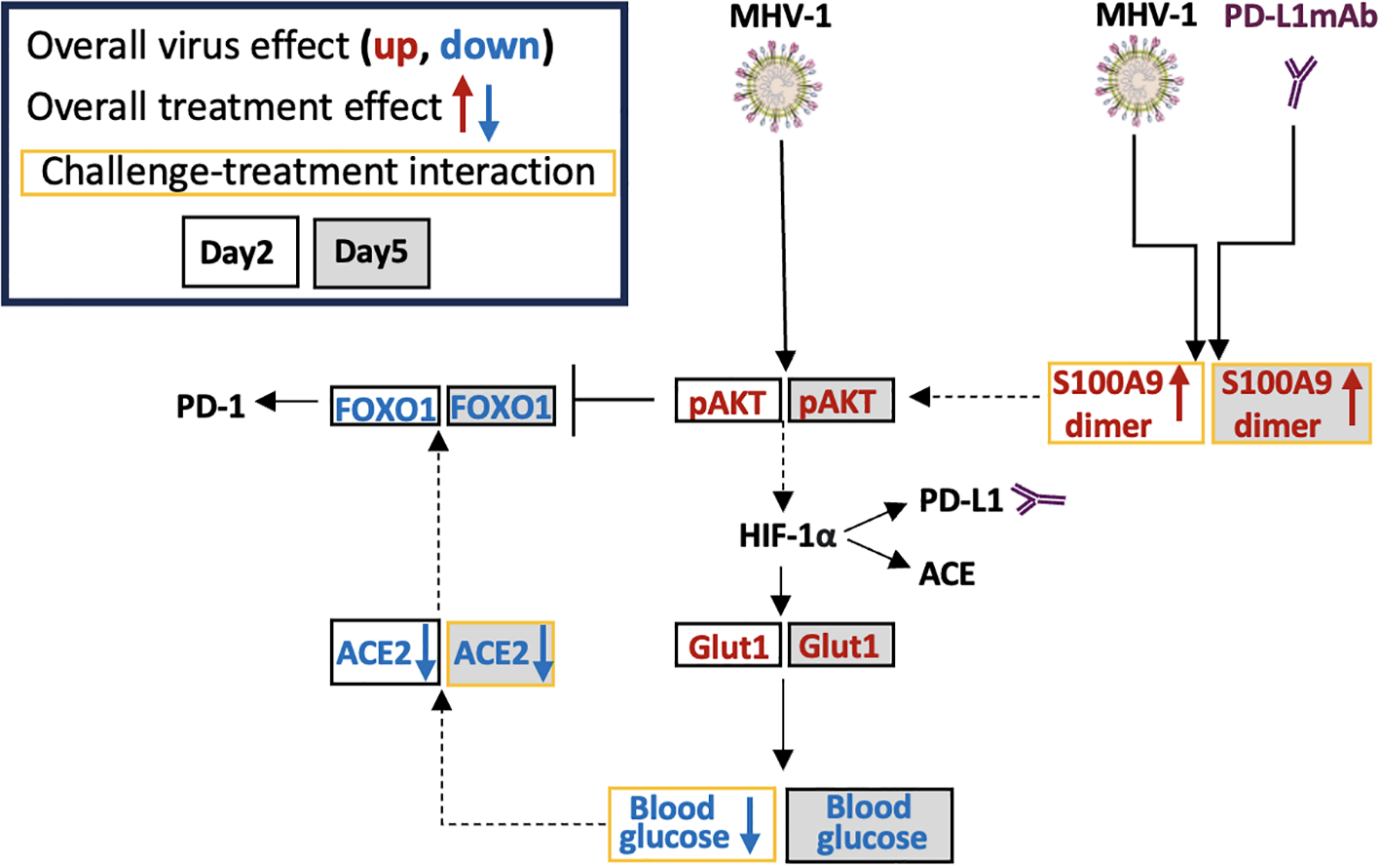
Possible MHV-1 and PD-L1mAb molecular signals. Mouse hepatitis virus 1 (MHV-1) is a pneumotropic beta-coronavirus that produces severe acute respiratory syndrome (SARS)-like pathology in A/J mice. Intraperitoneal injected anti-PD-L1 antibodies (PD-L1mAb) or isotype antibodies (isomAb) were administered every third day, starting 12 days before and continuing until 3 days after intratracheal infection with saline vehicle control or 50 plaque-forming units (PFU)/mouse. The overall virus effect measured the MHV-1 effect in mice challenged with MHV-1 and treated with isomAb and PD-L1mAb. The overall treatment effect measured the effects of PD-L1mAb in MHV-1- and diluent (saline)-challenged mice. The challenge–treatment interaction measured the difference between the overall virus effect and the overall treatment effect. MHV-1 activates/phosphorylates AKT (pAKT). PD-L1mAb and/or MHV-1 induce the production of the S100A9 dimer, which also activates AKT. Downstream of AKT is hypoxia-inducible transcription factor (HIF)-1α, which induces the production of PD-L1, angiotensin-converting enzyme (ACE), and glucose transporter 1 (Glut1). Cellular uptake of glucose from the surrounding microenvironment is regulated by Glut1. Blood glucose levels are associated with ACE2 levels and both of these factors are down-regulated by PD-L1mAb and/or MHV-1. Increased ACE2 activity stabilizes FOXO1, whereas AKT phosphorylation of FOXO1 promotes FOXO1 degradation. FOXO1 is a PD-1 regulatory transcription factor.

Although MHV-1 tended to increase S100A9 dimer levels at day 2 and 5, these increases were substantially greater with PD-L1mAb treatment on both days (*p* ≤ 0.001 challenge–treatment interactions). PD-L1mAb decreased ACE2 in uninfected but not infected animals at day 2 and 5 (*p* = 0.02 challenge–treatment interaction). Consistent with MHV-1-induced GLUT1 at day 2, blood glucose levels decreased in infected but not uninfected animals (*p* = 0.03 challenge–treatment interaction) and exhibited an overall treatment effect at day 5 (*p*< 0.001). Taken together, these data suggest that PD-L1mAb enhanced hypoxic responses (**Figure 8**).

## Discussion

Based on ICIs’ potent immunostimulatory effects, an ongoing question since the SARS-CoV-2 outbreak has been whether recent therapy with the agents worsens, improves, or has no effect on outcomes in cancer patients presenting with COVID-19. In a systematic review of 42 observational clinical studies addressing this question, we found no clear impact of prior ICI therapy on survival, severe events, or hospitalization, but the level of evidence was very low due largely to limited adjusted outcome analysis in the studies (1).

*In-vitro* studies have indicated that anti-PD-1 blockade increases the activation of T cells and reduces the percentage of exhausted T cells, suggesting a potential therapeutic benefit of ICIs in COVID-19 (70). In our meta-analysis of patients with prior ICI therapy, we assessed whether the impact of ICIs alters outcomes for cancer patients with COVID-19 (71). In this study, ICI patients required less COVID-19-related hospitalization and oxygen therapy and developed fewer complications supporting the role of ICIs in improving outcomes in COVID-19. However, ICI patients did not exhibit differences in mortality compared with non-ICI patients in the study.

Current *in-vivo* models of coronavirus infection involving the use of ICIs have assessed anti-PD-L1 (100 µg, unreported administration route) treatment 1 day before and after SARS-CoV-2 infection (unreported concentration and administration route) in adeno-associated virus (AAV)-expressing human ACE2 mice (72) or the administration of anti-PD-1 (200 µg, IP) for 3 days commencing upon MHV-A59 infection (800 PFU, IP) (73). These models cannot recapitulate natural respiratory infection and are not based on established pretreatment regimens associated with cancer therapy.

We therefore investigated the effects of MHV-1 (50 PFU, IT) in challenged A/J mice pretreated with anti-PD-L1 (300 μg, IP). We are the first to employ this model to examine the patterns of checkpoint molecule expression and markers of immune activation on immune cells and the effects of pretreatment with ICIs in a pneumonia model. Consistent with the findings from our systematic review, our controlled *in-vivo* study, involving a regimen of PD-L1mAb shown previously to have antineoplastic effects (25), did not alter survival. However, this study provides new insights into the pathogenesis of MHV-1 not previously explored.

In Study 1, following IT inoculation with a 50% lethal dose of MHV-1, infected animals demonstrated reduced weights and temperatures as early as day 2 and lower oxygen saturation evident at day 5. Our model also demonstrated peripheral lymphopenia, BAL lymphocytosis, abnormal coagulation parameters, vascular inflammation, and the presence of thrombi histologically, which are manifestations that recapitulate the findings in patients with COVID-19 (56–58).

We identified increased production of both proinflammatory and anti-inflammatory cytokines and chemokines in the serum and BAL up to day 10 after the challenge in this MHV-1 pneumonia model. Similar patterns of cytokine change have been noted in various studies of COVID-19 (26, 63, 70, 74–77). Generally, but not in all cases, these cytokine increases in infected animals here had diminished during recovery at day 10 in survivors. Similar decreases in circulating cytokine levels have been noted in patients recovering from COVID-19 (78). Notably, IL-6 has been closely associated with outcomes in COVID-19 patients (79). Lastly, we identified altered production of BAL cytokines (IFN-γ, IL-9, and IL-1) that were also isolated in the BAL of patients with COVID-19-related acute respiratory distress syndrome (27). These multiple associations with COVID-19 data further validate the utility of our model.

The presence of myeloid chemokines in the serum and BAL of animals infected with MHV-1 starting as early as day 2 post-infection highlights the importance of myeloid cells in the pathogenicity of the model and as identified in COVID-19 (80). Increased BAL IL-13, IL-5, and eotaxin at day 5 suggest increased activation and recruitment of eosinophils which have been implicated in COVID-19 pathogenesis (81). COVID-19 patients exhibit hyperactive Th1, Th2, and Th17 cytokine responses (4), and here, we detected this signature in both serum and BAL.

Examination of the effects of MHV-1 on immune cell surface markers revealed induction of PD-L1 on most immune subsets, indicating activation of these cells (82). These results in our lethal model are also consistent with elevated histological PD-L1 in lung tissue from patients who died from COVID-19 (83). PD-1 induction on lung CD4 and CD8 T cells occurred at day 5 and 10, respectively, indicating antigen exposure (84).

Also similar to SARS-CoV-2, MHV-1 reduced the detection of its receptor, CD66a, on most immune subsets. CD66a downregulation on monocytes is associated with hyper IL-6 responses and temperature depression (85), suggesting that increased IL-6 production and temperature depression in our study may be similarly associated. Loss of CD66a in neutrophils is inversely associated with neutrophil IL-1β production (86), and in response to MHV-1, neutrophil CD66a decreased and IL-1β production increased. Likewise, MHV-1 reduced CD66a on lung and splenic CD19+ B cells, reflecting their activation (87).

In neutrophils (88), monocytes, and macrophages (89), ACE is an activation marker. ACE may also regulate renin–angiotensin activity (18) or act as an endogenous enzyme in the processing of peptides for presentation via major histocompatibility classes I (90) and II (91). In response to MHV-1, ACE surface expression significantly increased on nearly all immune cells tested.

In Study 2, we examined the effects of MHV-1 in the presence of PD-L1mAb or isotype control pretreatment. Changes in isotype-treated MHV-1-infected animals strongly paralleled the findings of MHV-1-challenged untreated animals in Study 1. PD-L1mAb pretreatment in infected animals significantly blocked PD-L1 detection. Compared with isomAb, PD-L1mAb pretreatment induced the upregulation of PD-1 on liver and spleen lymphocytes, suggesting heightened systemic inflammation.

CD66a downregulation was enhanced in the lung of PD-L1mAb-treated animals, suggesting increased myeloid (86) and B-cell activation (87). CD66a isoforms with a long cytoplasmic tail contain two immunoreceptor tyrosine-based inhibitory motifs (ITIMs) to transmit inhibitory signals (92), similar to the checkpoint receptors PD-1 and cytotoxic T-lymphocyte associate (CTLA)-4 (93). Reduced CD66a may therefore affect CD66a trans-homophilic and/or trans-heterophilic signals that decrease the activity of the immunological synapse and inflammation (92). This may include CD66a negative regulation of TLR signals (86) that potentiate PD-L1 surface expression (94). Possibly, CD66a downregulation in response to PD-L1mAb in our study is a compensatory mechanism to induce cell signals associated with PD-L1 surface expression. Further investigation of the crosstalk between PD-L1 and CD66a may lead to better therapies that target the PD-1 axis or CD66a.

In response to MHV-1 and/or PD-L1mAb in our model, ACE was induced on immune cell subsets. Like PD-L1 (82), ACE is transcriptionally activated by hypoxia-inducible factor 1alpha (HIF-1α) (95), reflecting hypoxic metabolism in the presence of MHV-1 and/or PD-L1mAb. Immune cell ACE contributes to antigen presentation, microbicidal activity, and reactive oxygen species production (96). As an enzyme, ACE competes with ACE2 for angiotensin substrates, resulting in proinflammatory and anti-inflammatory downstream responses, respectively (18). Because ACE2 was inhibited by MHV-1 and/or PD-L1mAb in our model, angiotensin substrates favor ACE cleavage and downstream proinflammatory responses, as occurs in COVID-19. Increased immune cell ACE and reduced lung tissue ACE2 in response to PD-L1mAb have not been previously described and may contribute to the function of ACE inhibitors during ICI therapy (30). Similarly, we are the first to describe MHV-1-induced immune cell ACE and reduced lung tissue ACE2. Because CD66a deficiency increases ACE production (22), reduced cell surface CD66a may contribute to higher expression levels of ACE in our model. This regulatory crosstalk between ACE and CD66a may also play a role in therapies that target these molecules.

Despite PD-L1mAb-induced increases in immune activation, weight gain, and O_2_Sat, survival was not affected. The lack of PD-L1mAb benefit may be due to PD-L1mAb-induced production of clotting factors [fibrinogen, D-dimer (18); cdh6 (66)] and markers of injury [liver injury score, Matn2 (56)]. We also identified molecular signs of tissue hypoxia, which is a confounding factor in the diagnosis and treatment of COVID-19 (97). Hypoxia induces the production of PD-L1 (98), ACE (95), and the alarmins, S100A8 and S100A9, which are associated with COVID-19 severity (68, 69). In our model, MHV-1 induced S100A9 dimer and significantly increased S100A8 production. With PD-L1mAb pretreatment, S100A9 dimers were significantly enhanced, possibly as a compensatory mechanism of PD-L1 blockade (99).

S100A9 activates AKT (100), a demonstrated downstream response to SARS-CoV-2 (101). S100A9 activation of AKT also phosphorylates and targets FOXO1 for nuclear export and proteasome degradation (102). Prolonged inflammatory conditions suppress ACE2 and its product Ang(1-7), which activates MasR and promotes FOXO1 stability and transcriptional activation of antioxidant genes (103). High glucose levels are associated with increased ACE2 levels in bronchial submucosal cells (104), and conversely, ACE2 deficiency has been linked to increased glucose utilization (104). In our model, MHV-1 increased AKT activation, inhibited FOXO1 and ACE2, and promoted GLUT1 production in association with a reduction in blood glucose levels. Whether ACE2, an endogenous inhibitor of ACE (18), has a role in cell surface ACE or FOXO1-induced PD-1 (105) requires further study.

In our Olink analysis, we identified several markers that have been associated with outcomes in COVID-19, including Vegfd (63), Tnfsf12 (TWEAK) (57), Ccl2, and Cxcl9 (106). In a bivariate genome-wide association study (GWAS), elevated *NOTCH1* in whole blood increased the risk of COVID-19 critical illness (107), and we identified increased production of the NOTCH1 ligand Dll1 and reduced detection of the inhibitor Dlk1. We also identified increased Tnfrsf11b, which is a discriminator of severe COVID-19 neurological symptoms in patients (108).

COVID-19 is associated with promoting radiculopathies (109), neuropathies (110), myopathies (111), and myasthenic syndromes (112). Here, when assessing the overall virus effect, we identified the production of the glial cell-derived neurotrophic factor (GDNF) and GDNF receptor alpha (GFRa1), which is a co-receptor in promoting GDNF neurite outgrowth and neuronal survival (49). Increased contactin 1 (Cntn1) and tenascin R (Tnr) form a complex to induce neuronal action potential (113). Higher levels of matrilin 2 (Matn2) may be released from neurons following injury (114). Adam23, a neuronal receptor that contributes to high-frequency firing, was elevated in the serum (115). Lastly, increased seizure-related 6 homolog-like 2 (Sez6l2) in the model may be linked to the molecules’ role in promoting persistent neuronal synapses through binding interactions with glutamate receptors and adducins (116). Similarly, pretreatment with PD-L1mAb induced the production of these neurological mediators in infected animals. ICI-related neurotoxicity includes radiculopathies, neuropathies, myopathies, and myasthenic syndromes (117). Thus, the neurotoxic responses associated with COVID-19 and ICI therapy may be linked to these mediators.

This study has potential limitations. Firstly, only one PD-L1mAb was assessed. Additional clones may react differentially with epitopes and Fc receptors in specific strains of mice, producing different outcomes. Other ICI targets (e.g., PD-1, CTLA-4) may also have distinct effects based on their specific mechanisms of action and tissue distribution. Secondly, the regimen used in our study was established in murine tumor models (31, 32), and exposure to the drug might not have been of sufficient duration to provide clinically evident outcomes in our model. However, the regimen blocked the detection of PD-L1 on most cells tested and had significant effects compared with isomAb on other markers of disease. Third, lethality in the model was high. However, with 93 animals each in the PD-L1mAb- and isomAb-treated groups (>180 animals total) and with virtually similar 20% survival in each group, there was ample power to demonstrate either a harmful or beneficial effect signal with treatment. Finally, increased inflammation, angiogenesis, and neuronal activity identified as the effects of PD-L1mAb by proteomic analysis were not fully explored mechanistically.

In conclusion, we have established a murine coronavirus lung injury model that recapitulates several of the features of COVID-19 and allowed us to assess changes in checkpoint molecule expression on tissue immune cells. In this model, prior treatment with PD-L1mAb did not influence survival, supporting clinical findings that prior ICI treatment may not adversely impact outcomes in cancer patients presenting with COVID-19 (1).

## Data availability statement

The original contributions presented in the study are included in the article/**Supplementary Material**. Further inquiries can be directed to the corresponding author.

## Ethics statement

The animal study was approved by the Clinical Center Animal Care and Use Committee. The study was conducted in accordance with the local legislation and institutional requirements.

## Author contributions

CC: Conceptualization, Data curation, Formal analysis, Investigation, Methodology, Supervision, Writing – original draft, Writing – review & editing. XC: Conceptualization, Data curation, Formal analysis, Investigation, Methodology, Project administration, Resources, Supervision, Validation, Writing – original draft, Writing – review & editing. YL: Conceptualization, Data curation, Formal analysis, Investigation, Methodology, Supervision, Validation, Writing – review & editing. MJ: Data curation, Formal analysis, Methodology, Writing – review & editing. JS: Formal analysis, Investigation, Methodology, Validation, Writing – review & editing. CD: Formal analysis, Investigation, Methodology, Validation, Writing – review & editing. SM: Data curation, Investigation, Writing – review & editing. VH: Conceptualization, Formal analysis, Investigation, Methodology, Validation, Visualization, Writing – review & editing. RD: Data curation, Writing – review & editing. SC: Data curation, Formal analysis, Investigation, Methodology, Writing – review & editing. CB: Data curation, Writing – review & editing. AI: Data curation, Formal analysis, Investigation, Methodology, Writing – review & editing. PE: Conceptualization, Formal analysis, Investigation, Methodology, Project administration, Supervision, Writing – original draft, Writing – review & editing. PT: Conceptualization, Data curation, Formal analysis, Investigation, Methodology, Project administration, Resources, Supervision, Validation, Writing – original draft, Writing – review & editing.

## Supplementary material

Supplementary Figure 1 Experimental scheme, intravascular staining protocol, and gating strategy. **(A)** Mice were intratracheally administered MHV-1 or saline diluent control. Anesthetized mice were administered a fluorescently labeled (AF780) CD45 antibody 3m prior to euthanasia to identify circulating immune cells. Single cell suspensions from digested lung tissue were stained *ex-vivo* with an additional fluorescently labeled (PerCP) CD45 antibody to identify tissue associated immune cells. The PerCP positive AF780 negative cells were gated for live cells and immune cell subsets as displayed. **(B)** Mice were similarly treated as in **(A)** but due to the vascularity of both liver and spleen, the AF780 CD45 fluorescent marker was ignored. Single cell suspensions from liver and spleen were harvested, gated for live cells and immune cell subsets as displayed.

Supplementary Figure 2 The dose effect of intratracheal mouse hepatitis virus-1 (MHV-1) on proportional survival of A/J mice. Kaplan-Meier survival curves were plotted with increasing doses of MHV-1 [plaque-forming units (PFU)/mouse] compared to diluent controls.

Supplementary Figure 3 Mean (±SEM) physiological parameters, blood neutrophils, coagulation tests, and percent animals with change in lung and liver histology in mice challenged with MHV-1 or diluent control. **(A)** Mean heart rate and **(B)** respiratory rate from animals challenged with diluent control or MHV-1 (n = 12-24 mice/group). **(C)** Blood neutrophil [log(cell/μl)] from animals challenged with diluent control or MHV-1 (n = 4-7 mice/group). **(D)** Serum D-Dimers [log(ng/ml)] and thrombin-anti-thrombin [TAT, log(ng/ml)] or mean tissue factor pathway inhibitor (TFPI, ng/ml) from animals challenged with diluent control or MHV-1 (n = 5-10 mice/group). **(E)** Percent animals with changes in lung and **(F)** liver histology from animals challenged with diluent control or MHV-1 (n = 4-7 mice/group). Each experimental chart represents 3-4 independent experiments. 0.01<*p≤0.05, 0.001<†p≤0.01 and §p≤0.001 for MHV-1 vs control. ‡p-value for overall challenge effect. #p-value for challenge-time interaction.

Supplementary Figure 4 MHV-1 induced immune cell phenotypes over time. Lung, liver, and spleen immune cells from diluent control and MHV-1 infected animals were assessed for cell surface markers (PD-L1, PD-1, CD66a, ACE) at 2d, 5d, and 10d. The median fluorescence intensities (MFIs) were obtained for each marker and the respective isotype MFIs were subtracted. Heatmaps of control and MHV-1-challenged animals are displayed with each square representing a single animal (n=6/group over 2 independent experiments). x̄ represents the mean intensity for the group and time; #p-values comparing MHV-1 vs control; **p-values for the challenge-time interaction (MHV-1* time). Identified markers, at each timepoint and for the virus effect at each timepoint, increased (red) or decreased (blue), p≤0.05.

Supplementary Figure 5 PD-L1mAb-induced immune cell phenotypes at 2 days after MHV-1 or diluent control challenge compared to isomAb challenge. Lung, liver, and spleen from isomAb or PDL1mAb pre-treated animals challenged with diluent control or MHV-1 at day 2 were assessed for cell surface markers (PD-L1, PD-1, CD66a, ACE). The median fluorescence intensities (MFIs) were obtained for each marker and the respective isotype MFIs were subtracted. Heatmaps are displayed with each square representing a single animal (n=6/group over 2 independent experiments). x̄ represents the mean intensity for the group and time. # - p-values for Isotype mAb vs PD-L1 mAb. **p-values for overall effect of the virus challenge (MHV-1), or PD-L1 mAb treatment (Treat) or the interaction of challenge and treatment (MHV-1*Treat). Identified markers: at each timepoint and for the overall virus effect, increased (red) or decreased (blue), p≤0.05. NA-not applicable effect due to a significant challenge-treatment interaction.

Supplementary Figure 6 PD-L1mAb-induced immune cell phenotypes at 5 days after MHV-1 or diluent control challenge compared to isomAb challenge. Lung, liver, and spleen from isomAb or PDL1mAb pre-treated animals challenged with diluent control or MHV-1 at day 5 were assessed for cell surface markers (PD-L1, PD-1, CD66a, ACE). The median fluorescence intensities (MFIs) were obtained for each marker and the respective isotype MFIs were subtracted. Heatmaps are displayed with each square representing a single animal (N=6/group over 4 independent experiments). x̄ represents the mean intensity for the group and time. # - p-values for Isotype mAb vs PD-L1 mAb. **p-values for overall effect of the virus challenge (MHV-1); or PD-L1 mAb treatment (Treat) or the interaction of challenge and treatment (MHV-1*Treat). Identified markers, at each timepoint and for the overall virus effect, increased (red) or decreased (blue), p≤0.05. NA-not applicable effect due to a significant challenge-treatment interaction.

Supplementary Figure 7 Effect of PD-L1mAb or isomAb pre-treatment on mean (±SEM) physiological parameters, lung and liver viral titers, complete blood cell counts, coagulation measures, bronchoalveolar lavage cell count and lung wet-dry ratio in mice challenged with MHV-1 or diluent control at 2 and 5 days. **(A)** O2 saturation (%), **(B)** heart rate, **(C)** respiratory rate, and **(D)** body temperature (°C) for animals pre-treated with isomAb or PD-L1mAb and challenged with diluent or MHV-1 (n = 19-33 mice/group). **(E)** Complete blood cell counts [log(cells/ul)] from animals pre-treated with isomAb or PD-L1mAb and challenged with diluent or MHV-1 (n = 7-16 mice/group). **(F)** Plasma fibrinogen (μg/ml), tissue factor pathway inhibitor (TFPI)(ng/ml), and D-Dimers, tissue factor (TF) and thrombin-anti-thrombin (TAT) [log(pg/ml)] from animals pre-treated with isomAb or PD-L1mAb diluent or challenged with diluent or MHV-1 (n = 8-12 mice/group). **(G)** Bronchoalveolar lavage cell counts [log(cells/μl)] and protein [log(μg/μl)] from animals pre-treated with isomAb or PD-L1mAb diluent or challenged with diluent or MHV-1 (n = 5-9 mice/group). **(H)** Lung wet-to-dry ratios from animals pretreated with isomAb or PD-L1mAb diluent or challenged with diluent or MHV-1 (n = 5-11 mice/group). Each experimental chart represents 3-4 independent experiments. 0.001<†p≤0.01 for PD-L1 mAb vs isomAb within each challenge. #p-value for overall challenge effect. **pvalues for challenge and treatment interaction. ‡p-value for overall treatment effect.

Supplementary Figure 8 Effect of PD-L1mAb or isomAb pre-treatment on lung and liver histology in mice challenged with MHV-1 or diluent control at 2 and 5 days. **(A)** Histological evaluation of lungs from animals pre-treated with isomAb or PD-L1 and challenged with diluent control or MHV-1, and **(B)** histological evaluation of livers from animals pre-treated with isomAb or PDL1 and challenged with diluent control or MHV-1 (n=3-11 mice/group over 3-4 independent experiments). 0.01<*p≤0.05, 0.001<†p≤0.01 for PD-L1mAb vs isomAb. #p-value for overall challenge effect. ‡p-value for overall treatment effect. **p-value for challenge and treatment interaction. HPF, high powered field.

Supplementary Figure 9 Effect of PD-L1mAb or isomAb pre-treatment on bronchoalveolar lavage mediators in mice challenged with MHV-1 or diluent control at 2 and 5 days. **(A)** Bronchoalveolar lavage fluid mediators ([log(ug/ml)] was assessed by Bioplex on animals pre-treated with isomAb or PD-L1mAb and challenged with diluent control or MHV-1 (n = 5-9 mice/group over 3-4 independent experiments). 0.01<*p≤0.05 for PD-L1mAb vs isomAb. #pvalue for overall challenge effect. ‡p-value for overall treatment effect.

Supplementary Figure 10 Lung cell signals in isomAb or PD-L1mAb pre-treated MHV-1 or diluent challenged mice. **(A–D)** Whole lung lysates were assessed by immunoblot for phosphorylated AKT (pAKT, threonine 308) and total AKT **(A)** and total FOXO1 **(B)**. Representative images and densitometry relative to total actin are displayed for 6 mice/group over 3 independent experiments. The ratio of pAKT/total AKT is also charted. #p-value for the overall challenge effect.
