# Supplementary material for: Anti-PD-L1 therapy altered inflammation but not survival in a lethal murine hepatitis virus-1 pneumonia model
Source: Front Immunol. 2024 Jan 8;14:1308358. doi: 10.3389/fimmu.2023.1308358 (PMC10801642; doi:10.3389/fimmu.2023.1308358)
Supplement: Supplementary Figure 1 — Experimental scheme, intravascular staining protocol, and gating strategy. (A) Mice were intratracheally administered MHV-1 or saline diluent control. Anesthetized mice were administered a fluorescently labeled (AF780) CD45 antibody 3m prior to euthanasia to identify circulating immune cells. Single cell suspensions from digested lung tissue were stained ex-vivo with an additional fluorescently labeled (PerCP) CD45 antibody to identify tissue associated immune cells. The PerCP positive AF780 negative cells were gated for live cells and immune cell subsets as displayed. (B) Mice were similarly treated as in (A) but due to the vascularity of both liver and spleen, the AF780 CD45 fluorescent marker was ignored. Single cell suspensions from liver and spleen were harvested, gated for live cells and immune cell subsets as displayed. [file DataSheet_1.docx]

***Supplementary Material***

**Anti-PD-L1 therapy altered inflammation but not survival in a lethal murine hepatitis virus-1 pneumonia model**

**Colleen S. Curran, Xizhong Cui, Yan Li, Mark Jeakle, Junfeng Sun, Cumhur Y. Demirkale, Samuel Minkove, Victoria Hoffmann, Rhea Dhamapurkar, Symya Chumbris, Cameron Bolyard, Akunna Iheanacho, Peter Q. Eichacker and Parizad Torabi-Parizi**

**Correspondence:** Parizad Torabi-Parizi: [parizad.torabi-parizi@nih.gov](mailto:parizad.torabi-parizi@nih.gov)

**
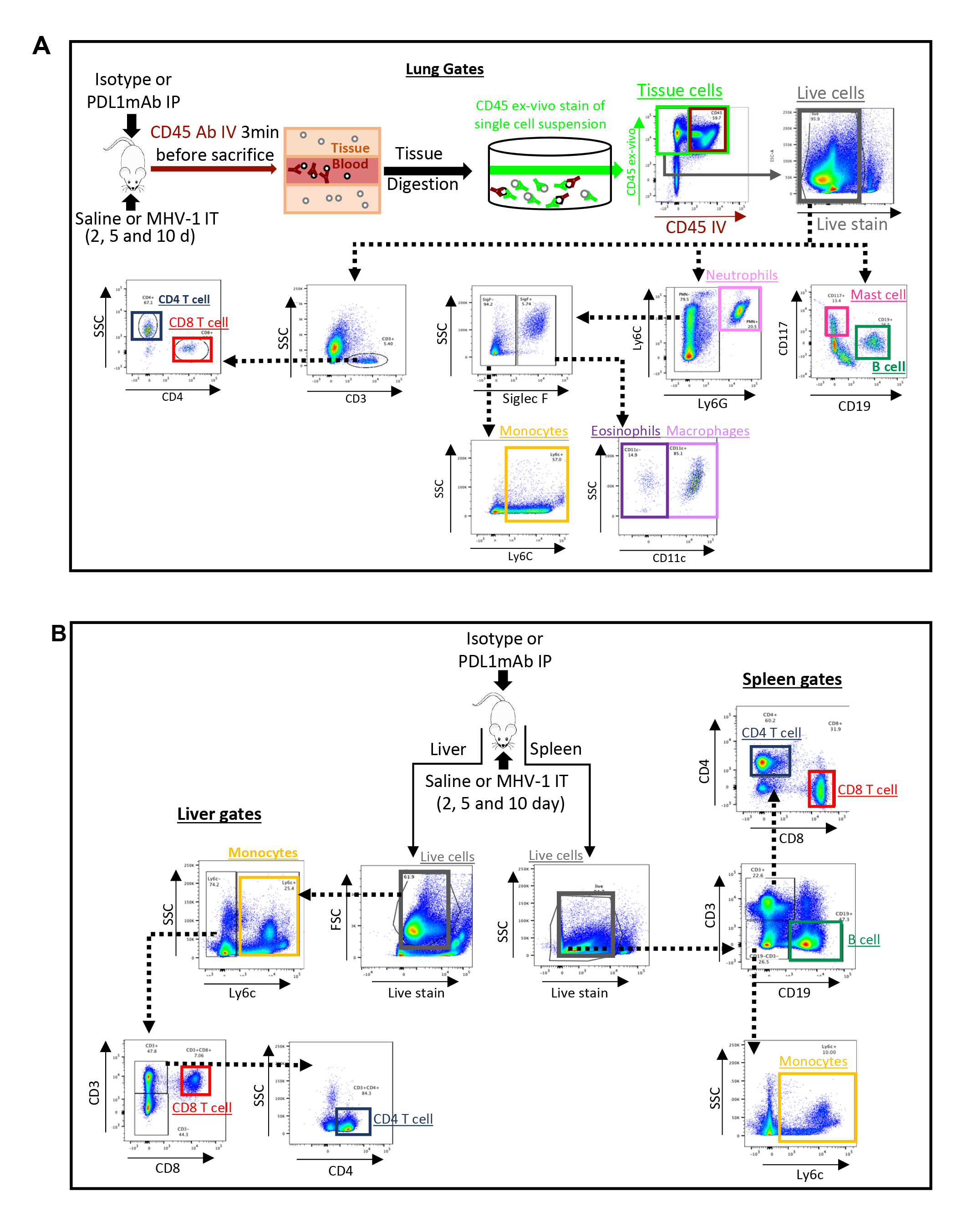
**

**SFigure 1. Experimental scheme, intravascular staining protocol, and gating strategy. (A**) Mice were intratracheally administered MHV-1 or saline diluent control. Anesthetized mice were administered a fluorescently labeled (AF780) CD45 antibody 3m prior to euthanasia to identify circulating immune cells. Single cell suspensions from digested lung tissue were stained ex-vivo with an additional fluorescently labeled (PerCP) CD45 antibody to identify tissue associated immune cells. The PerCP positive AF780 negative cells were gated for live cells and immune cell subsets as displayed. (**B**) Mice were similarly treated as in (A) but due to the vascularity of both liver and spleen, the AF780 CD45 fluorescent marker was ignored. Single cell suspensions from liver and spleen were harvested, gated for live cells and immune cell subsets as displayed.

**
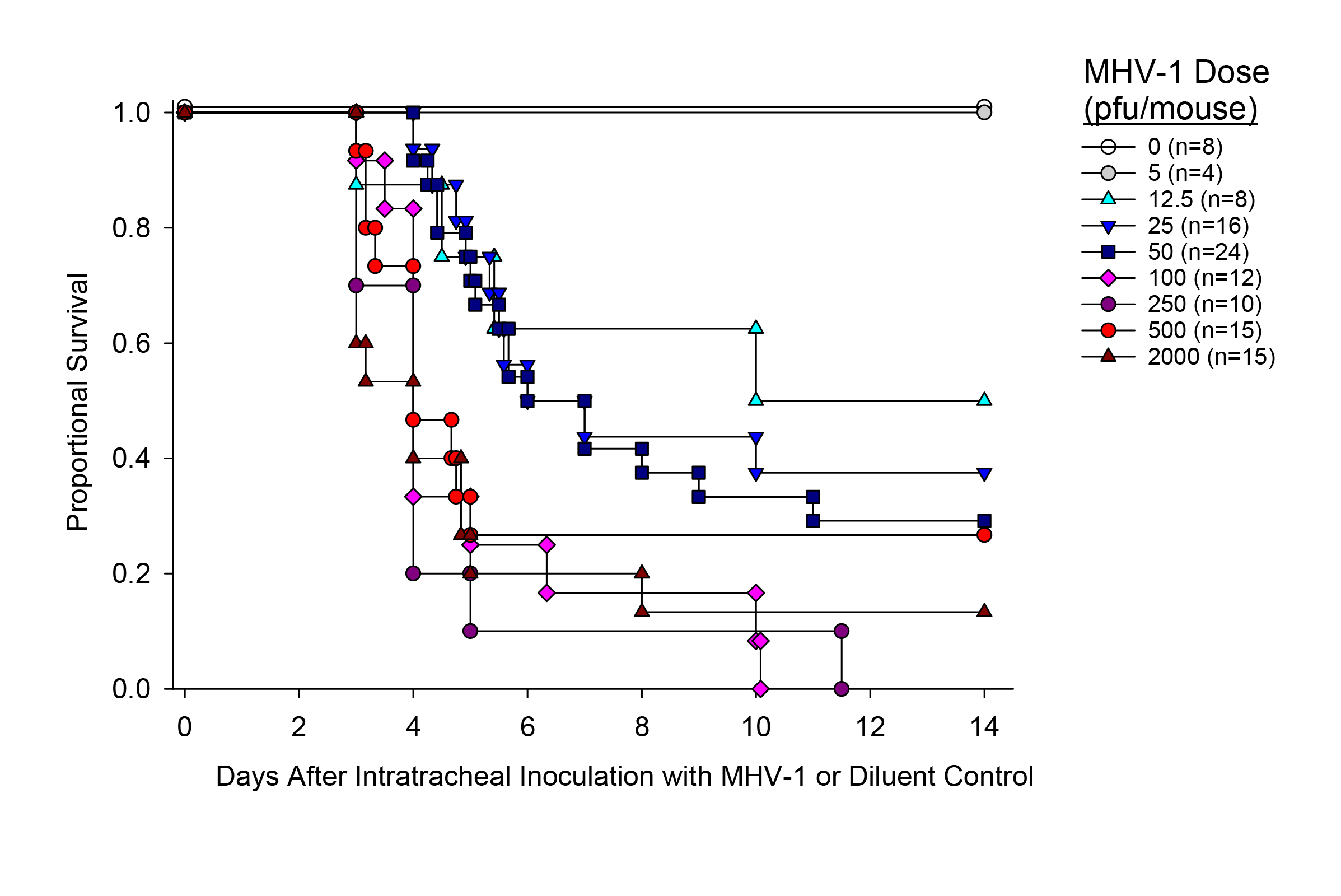
**

**SFigure 2. The dose effect of intratracheal mouse hepatitis virus-1 (MHV-1) on proportional survival of A/J mice.** Kaplan-Meier survival curves were plotted with increasing doses of MHV-1 [plaque-forming units (PFU)/mouse] compared to diluent controls.

**
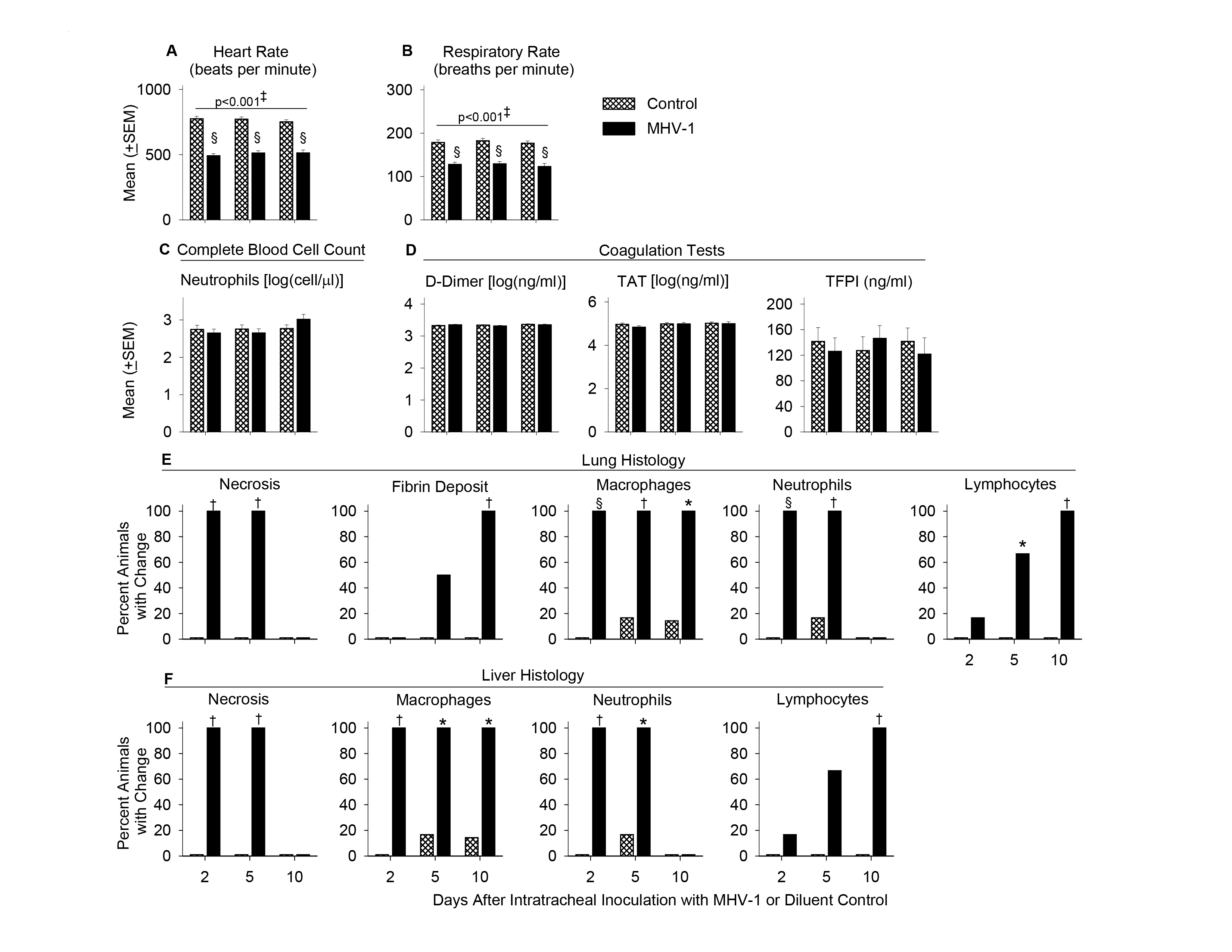
**

**SFigure 3. Mean (±SEM) physiological parameters, blood neutrophils, coagulation tests, and percent animals with change in lung and liver histology in mice challenged with MHV-1 or diluent control.** (**A**) Mean heart rate and (**B**) respiratory rate from animals challenged with diluent control or MHV-1 (n = 12-24 mice/group). (**C**) Blood neutrophil [log(cell/μl)] from animals challenged with diluent control or MHV-1 (n = 4-7 mice/group). (**D**) Serum D-Dimers [log(ng/ml)] and thrombin-anti-thrombin [TAT, log(ng/ml)] or mean tissue factor pathway inhibitor (TFPI, ng/ml) from animals challenged with diluent control or MHV-1 (n = 5-10 mice/group). (**E**) Percent animals with changes in lung and (**F**) liver histology from animals challenged with diluent control or MHV-1 (n = 4-7 mice/group). Each experimental chart represents 3-4 independent experiments. 0.01<*p≤0.05, 0.001<†p≤0.01 and §p≤0.001 for MHV-1 vs control. ‡p-value for overall challenge effect. #p-value for challenge-time interaction.


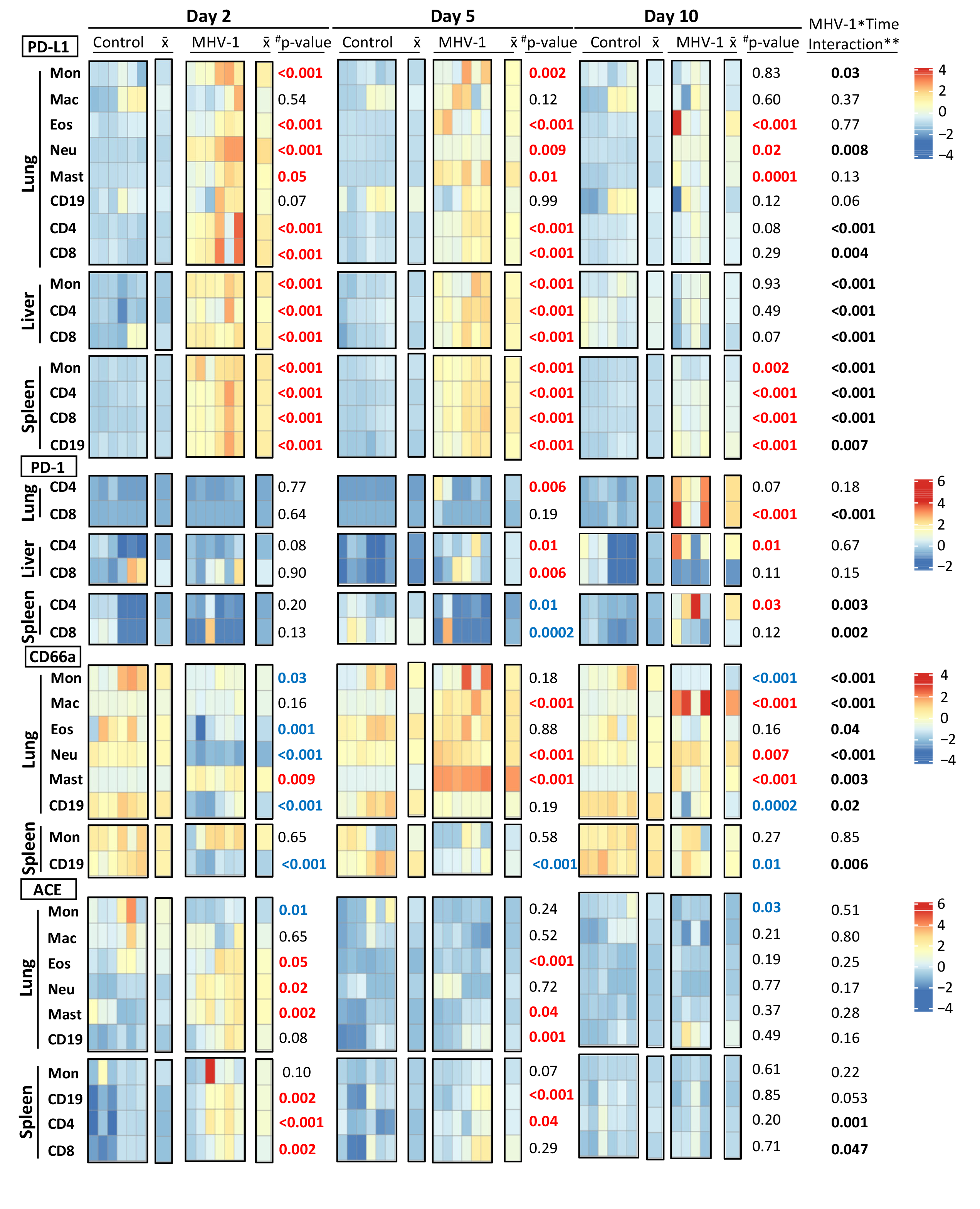


**SFigure 4. MHV-1 induced immune cell phenotypes over time.** Lung, liver, and spleen immune cells from diluent control and MHV-1 infected animals were assessed for cell surface markers (PD-L1, PD-1, CD66a, ACE) at 2d, 5d, and 10d. The median fluorescence intensities (MFIs) were obtained for each marker and the respective isotype MFIs were subtracted. Heatmaps of control and MHV-1-challenged animals are displayed with each square representing a single animal (n=6/group over 2 independent experiments). x̄ represents the mean intensity for the group and time; #p-values comparing MHV-1 vs control; **p-values for the challenge-time interaction (MHV-1* time). Identified markers, at each timepoint and for the virus effect at each timepoint, increased (red) or decreased (blue), p≤0.05.

**
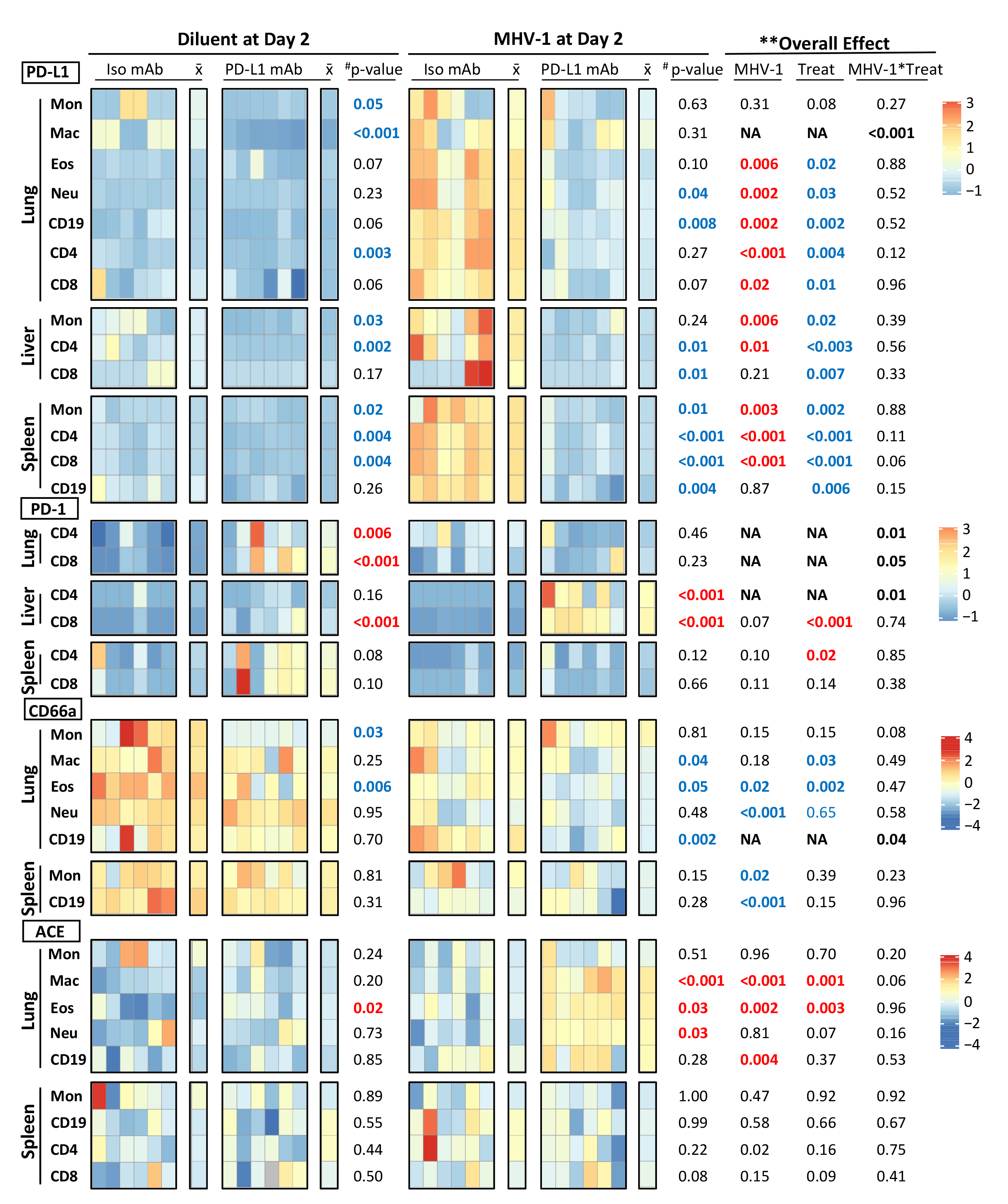
**

**SFigure 5. PD-L1mAb-induced immune cell phenotypes at 2 days after MHV-1 or diluent control challenge compared to isomAb challenge.** Lung, liver, and spleen from isomAb or PD-L1mAb pre-treated animals challenged with diluent control or MHV-1 at day 2 were assessed for cell surface markers (PD-L1, PD-1, CD66a, ACE). The median fluorescence intensities (MFIs) were obtained for each marker and the respective isotype MFIs were subtracted. Heatmaps are displayed with each square representing a single animal (n=6/group over 2 independent experiments). x̄ represents the mean intensity for the group and time. # - p-values for Isotype mAb vs PD-L1 mAb. **p-values for overall effect of the virus challenge (MHV-1), or PD-L1 mAb treatment (Treat) or the interaction of challenge and treatment (MHV-1*Treat). Identified markers: at each timepoint and for the overall virus effect, increased (red) or decreased (blue), p≤0.05. NA-not applicable effect due to a significant challenge-treatment interaction.


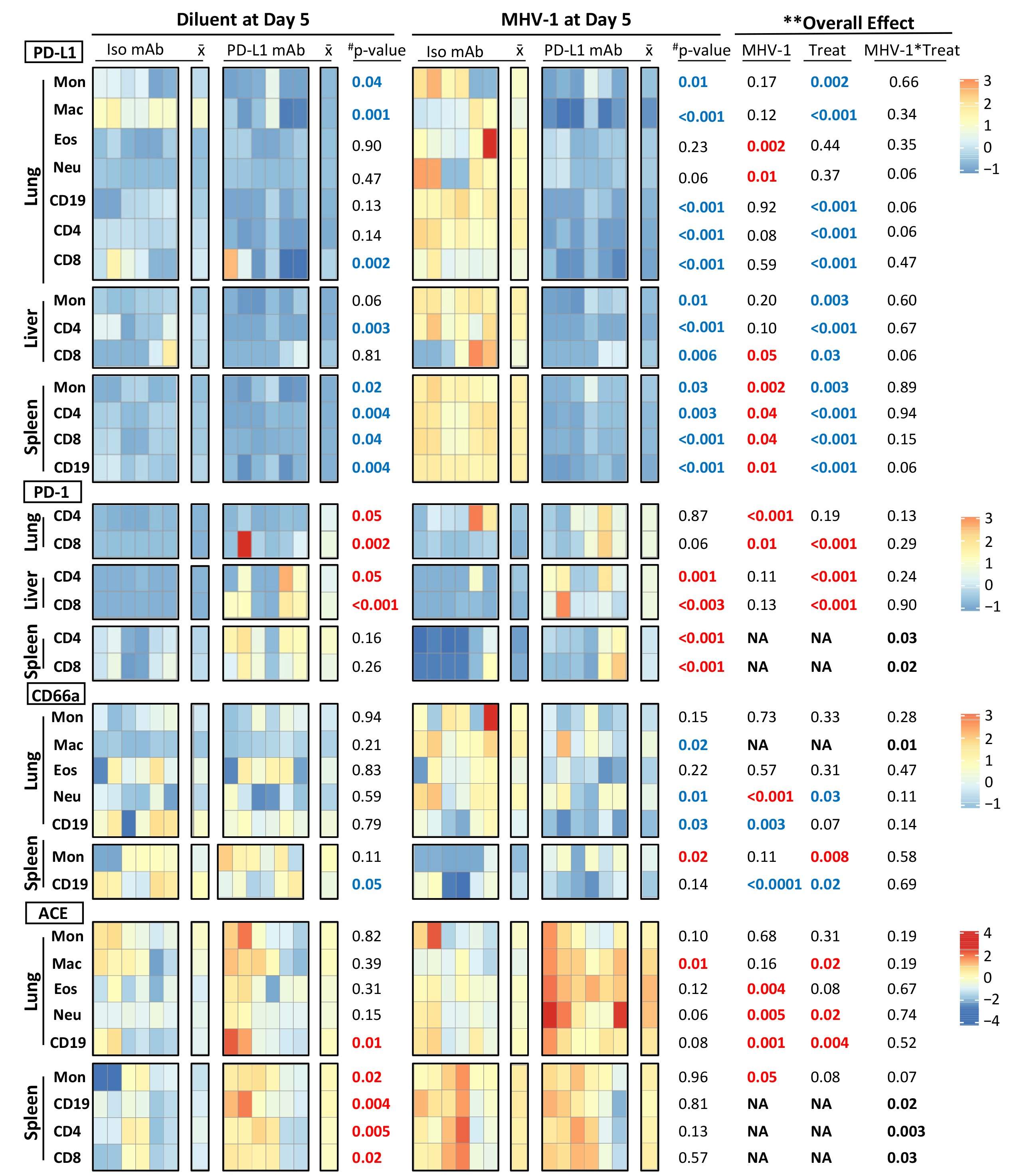


**SFigure 6. PD-L1mAb-induced immune cell phenotypes at 5 days after MHV-1 or diluent control challenge compared to isomAb challenge.** Lung, liver, and spleen from isomAb or PD-L1mAb pre-treated animals challenged with diluent control or MHV-1 at day 5 were assessed for cell surface markers (PD-L1, PD-1, CD66a, ACE). The median fluorescence intensities (MFIs) were obtained for each marker and the respective isotype MFIs were subtracted. Heatmaps are displayed with each square representing a single animal (N=6/group over 4 independent experiments). x̄ represents the mean intensity for the group and time. # - p-values for Isotype mAb vs PD-L1 mAb. **p-values for overall effect of the virus challenge (MHV-1); or PD-L1 mAb treatment (Treat) or the interaction of challenge and treatment (MHV-1*Treat). Identified markers, at each timepoint and for the overall virus effect, increased (red) or decreased (blue), p≤0.05. NA-not applicable effect due to a significant challenge-treatment interaction.

**
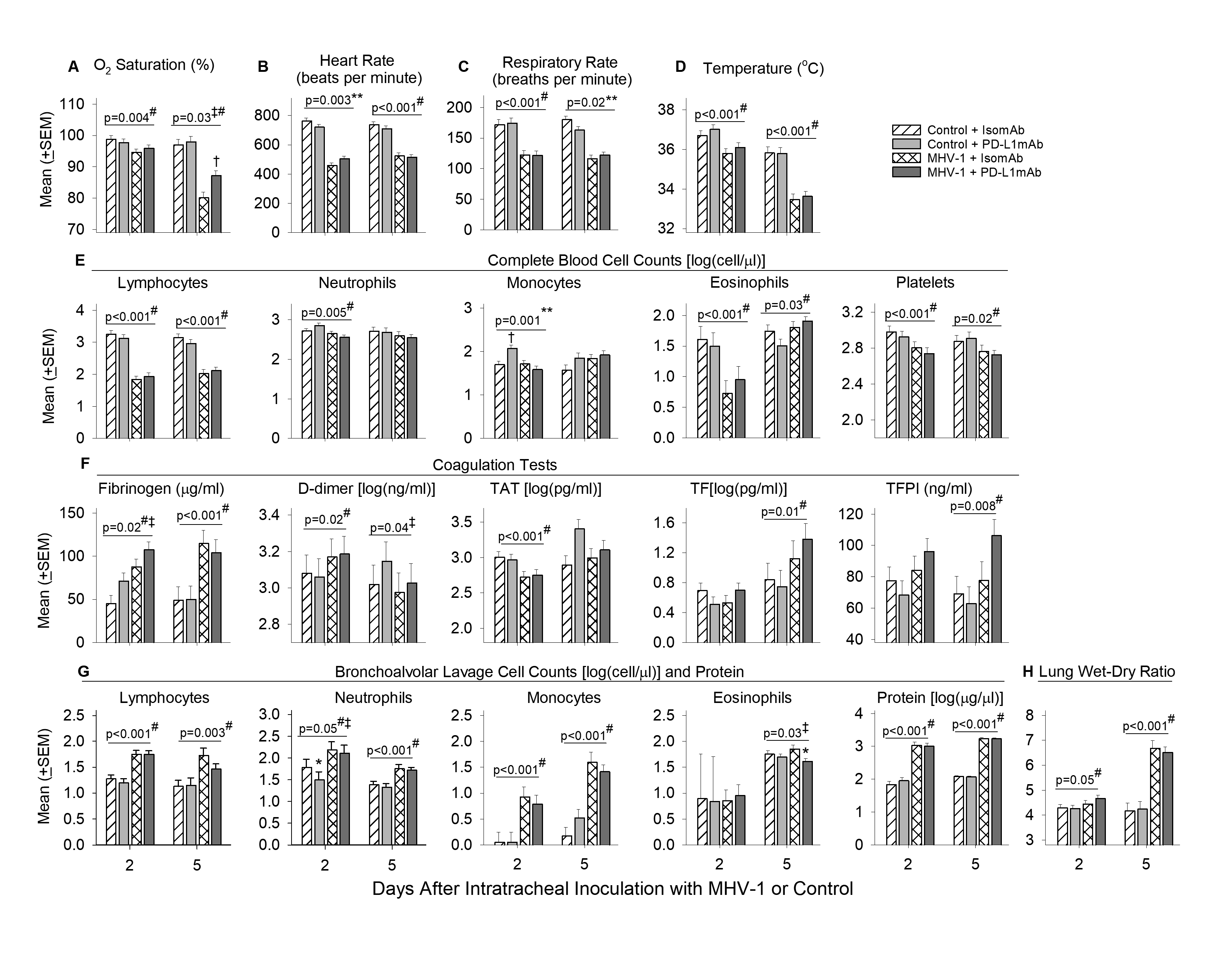
**

**SFigure 7. Effect of PD-L1mAb or isomAb pre-treatment on mean (±SEM) physiological parameters, lung and liver viral titers, complete blood cell counts, coagulation measures, bronchoalveolar lavage cell count and lung wet-dry ratio in mice challenged with MHV-1 or diluent control at 2 and 5 days**. (**A**) O_2_ saturation (%), (**B**) heart rate, (**C**) respiratory rate, and (**D**) body temperature (°C) for animals pre-treated with isomAb or PD-L1mAb and challenged with diluent or MHV-1 (n = 19-33 mice/group). (**E**) Complete blood cell counts [log(cells/ul)] from animals pre-treated with isomAb or PD-L1mAb and challenged with diluent or MHV-1 (n = 7-16 mice/group). (**F**) Plasma fibrinogen (μg/ml), tissue factor pathway inhibitor (TFPI)(ng/ml), and D-Dimers, tissue factor (TF) and thrombin-anti-thrombin (TAT) [log(pg/ml)] from animals pre-treated with isomAb or PD-L1mAb diluent or challenged with diluent or MHV-1 (n = 8-12 mice/group). (**G**) Bronchoalveolar lavage cell counts [log(cells/μl)] and protein [log(μg/μl)] from animals pre-treated with isomAb or PD-L1mAb diluent or challenged with diluent or MHV-1 (n = 5-9 mice/group). (**H**) Lung wet-to-dry ratios from animals pre-treated with isomAb or PD-L1mAb diluent or challenged with diluent or MHV-1 (n = 5-11 mice/group). Each experimental chart represents 3-4 independent experiments. 0.001<†p≤0.01 for PD-L1 mAb vs isomAb within each challenge. #p-value for overall challenge effect. **p-values for challenge and treatment interaction. ‡p-value for overall treatment effect.

**
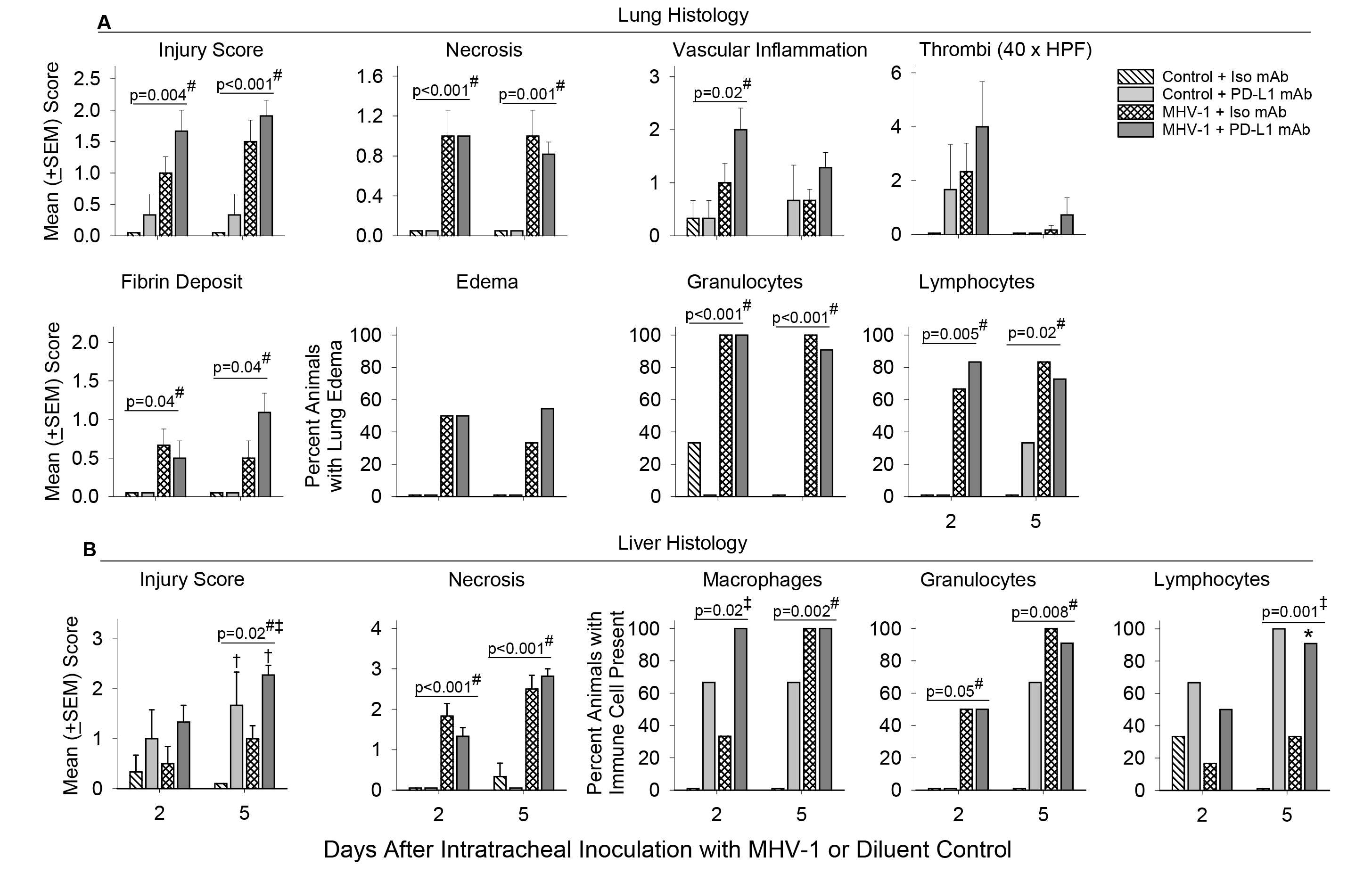
**

**SFigure 8. Effect of PD-L1mAb or isomAb pre-treatment on lung and liver histology in mice challenged with MHV-1 or diluent control at 2 and 5 days.** (**A**) Histological evaluation of lungs from animals pre-treated with isomAb or PD-L1 and challenged with diluent control or MHV-1, and (**B**) histological evaluation of livers from animals pre-treated with isomAb or PD-L1 and challenged with diluent control or MHV-1 (n=3-11 mice/group over 3-4 independent experiments). 0.01<*p≤0.05, 0.001<†p≤0.01 for PD-L1mAb vs isomAb. #p-value for overall challenge effect. ‡p-value for overall treatment effect. **p-value for challenge and treatment interaction. HPF, high powered field.

**
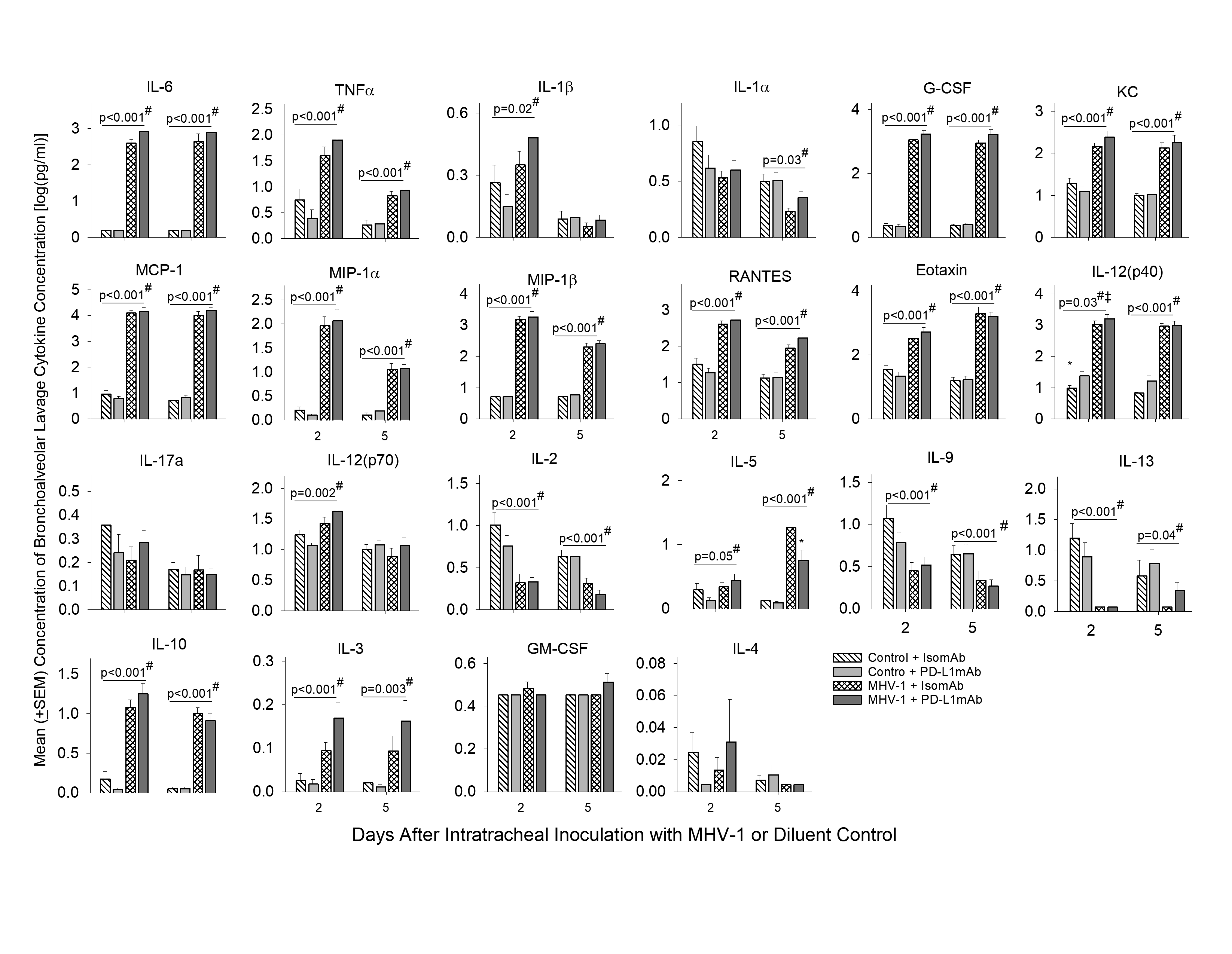
SFigure 9. Effect of PD-L1mAb or isomAb pre-treatment on bronchoalveolar lavage mediators in mice challenged with MHV-1 or diluent control at 2 and 5 days.** (**A**) Bronchoalveolar lavage fluid mediators ([log(ug/ml)] was assessed by Bioplex on animals pre-treated with isomAb or PD-L1mAb and challenged with diluent control or MHV-1 (n = 5-9 mice/group over 3-4 independent experiments). 0.01<*p≤0.05 for PD-L1mAb vs isomAb. #p-value for overall challenge effect. ‡p-value for overall treatment effect.

**
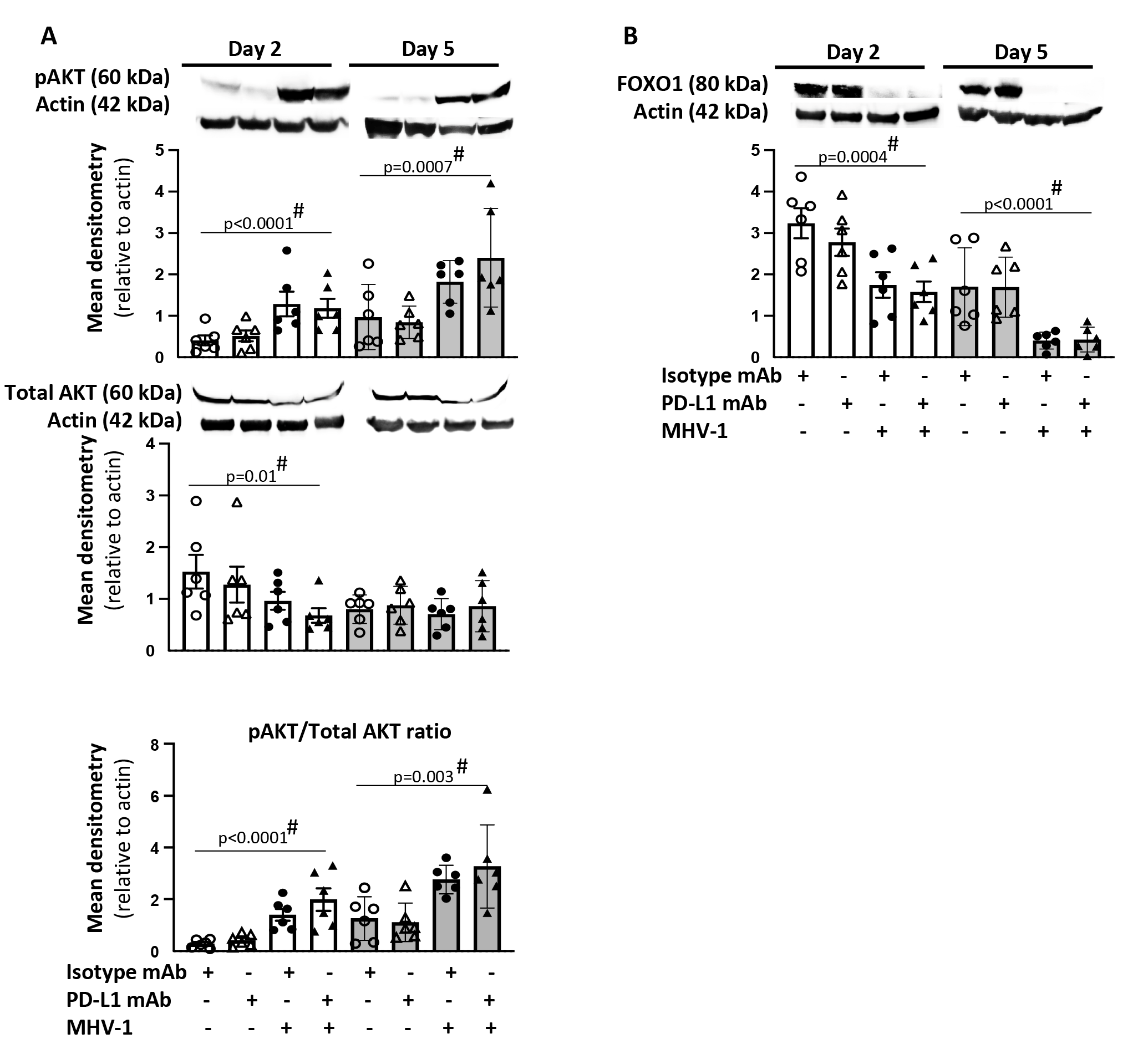
**

**SFigure 10. Lung cell signals in isomAb or PD-L1mAb pre-treated MHV-1 or diluent challenged mice.** (**A-D**) Whole lung lysates were assessed by immunoblot for phosphorylated AKT (pAKT, threonine 308) and total AKT (**A**) and total FOXO1 (**B**). Representative images and densitometry relative to total actin are displayed for 6 mice/group over 3 independent experiments. The ratio of pAKT/total AKT is also charted. #p-value for the overall challenge effect.
