# Supplementary material for: Anti-PD-L1 therapy altered inflammation but not survival in a lethal murine hepatitis virus-1 pneumonia model
Source: Front Immunol. 2024 Jan 8;14:1308358. doi: 10.3389/fimmu.2023.1308358 (PMC10801642; doi:10.3389/fimmu.2023.1308358)
Supplement: Supplementary file 2 [file Table_1.docx]

| **Supplemental Table 1. Mean (±SEM) serum and bronchoalveolar lavage (BAL) cytokines levels [log(pg/µl)] with challenge of MHV-1 or its diluent (Control) at Day 2, 5 and 10** | | | | | | | | | | | | | | | |
| --- | --- | --- | --- | --- | --- | --- | --- | --- | --- | --- | --- | --- | --- | --- | --- |
| Cytokines |  | Serum | | | | |  |  |  | BAL | | | | |  |
|  |  | Control |  |  |  | MHV-1 |  |  |  | Control |  |  |  | MHV-1 |  |
|  | 2 | 5 | 10 |  | 2 | 5 | 10 |  | 2 | 5 | 10 |  | 2 | 5 | 10 |
| IL-6 | 0.15±0.08 | 0.10±0.04 | 0.34±0.15 |  | 1.33±0.09^§^ | 1.62±0.17^§^ | 0.75±0.22^*^ |  | 0.21±0.07 | 0.18±0.08 | 0.20±0.04 |  | 2.54±0.05^§^ | 2.35±0.15^§^ | 0.76±0.23^§^ |
| TNFα | 1.11±0.10 | 1.24±0.06 | 1.27±0.12 |  | 1.54±0.05^†^ | 1.51±0.17^*^ | 1.80±0.20^†^ |  | 0.64±0.13 | 0.56±0.10 | 0.71±0.13 |  | 1.69±0.12^§^ | 0.95±0.07^†^ | 1.22±0.07^†^ |
| IL-1β | 0.24±0.04 | 0.24±0.03 | 0.29±0.02 |  | 0.32±0.03 | 0.38±0.03^†^ | 0.42±0.04^*^ |  | 0.07±0.02 | 0.07±0.02 | 0.13±0.01 |  | 0.48±0.05^§^ | 0.10±0.02 | 0.09±0.03 |
| IL-1α | 0.14±0.03 | 0.17±0.06 | 0.27±0.08 |  | 0.38±0.08^*^ | 0.41±0.12^†^ | 0.57±0.13^†^ |  | 0.78±0.05 | 0.77±0.09 | 0.84±0.07 |  | 0.71±0.04 | 0.27±0.02^§^ | 0.22±0.05^§^ |
| G-CSF | 1.20±0.09 | 1.30±0.02 | 1.96±0.20 |  | 3.00±0.04^§^ | 2.86±0.07^§^ | 2.13±0.32 |  | 0.23±0.05 | 0.24±0.08 | 0.51±0.08 |  | 2.78±0.04^§^ | 2.65±0.06^§^ | 1.80±0.17^§^ |
| KC | 1.46±0.04 | 1.44±0.03 | 1.54±0.10 |  | 1.99±0.05^§^ | 2.09±0.11^§^ | 1.75±0.10^*^ |  | 0.91±0.04 | 0.92±0.05 | 1.32±0.06 |  | 2.16±0.05^§^ | 1.93±0.06^§^ | 1.69±0.03^§^ |
| MCP-1 | 1.74±0.09 | 1.73±0.09 | 2.00±0.09 |  | 3.60±0.04^§^ | 3.19±0.09^§^ | 2.65±0.07^§^ |  | 0.80±0.09 | 0.75±0.03 | 0.81±0.07 |  | 4.05±0.03^§^ | 3.74±0.10^§^ | 2.91±0.28^§^ |
| MIP-1α | 0.21±0.03 | 0.25±0.03 | 0.26±0.04 |  | 0.87±0.04^§^ | 0.81±0.05^§^ | 0.74±0.08^§^ |  | 0.10±0.02 | 0.12±0.05 | 0.11±0.02 |  | 2.32±0.13^§^ | 0.85±0.11^§^ | 0.99±0.08^§^ |
| MIP-1β | 1.38±0.13 | 1.32±0.13 | 1.26±0.20 |  | 2.58±0.03^§^ | 2.48±0.05^§^ | 2.20±0.08^§^ |  | 1.35±0.17 | 1.31±0.19 | 1.34±0.10 |  | 3.27±0.07^§^ | 1.99±0.13^§^ | 1.72±0.10 |
| RANTES | 1.78±0.05 | 1.75±0.05 | 1.72±0.04 |  | 2.50±0.03^§^ | 2.32±0.03^§^ | 2.16±0.09^§^ |  | 1.04±0.15 | 0.98±0.08 | 1.26±0.06 |  | 2.50±0.08^§^ | 1.61±0.11^§^ | 1.99±0.11^§^ |
| Eotaxin | 2.88±0.07 | 2.72±0.06 | 2.70±0.03 |  | 2.97±0.03 | 3.00±0.08^†^ | 3.18±0.07^§^ |  | 1.01±0.12 | 1.07±0.06 | 1.23±0.07 |  | 1.98±0.07^§^ | 2.41±0.12^§^ | 2.29±0.14^§^ |
| IL12(p40) | 2.61±0.04 | 2.65±0.05 | 2.56±0.05 |  | 3.23±0.03^§^ | 3.22±0.09^§^ | 2.49±0.08 |  | 0.72±0.09 | 0.76±0.08 | 0.81±0.08 |  | 2.73±0.04^§^ | 2.47±0.09^§^ | 2.21±0.13^§^ |
| IL-17a | 1.50±0.08 | 1.51±0.05 | 1.64±0.10 |  | 1.73±0.05 | 1.56±0.17 | 1.82±0.22 |  | 0.06±0.04 | 0.04±0.02 | 0.04±0.01 |  | 0.14±0.02^†^ | 0.06±0.02 | 0.11±0.03^*^ |
| IL12(p70) | 0.86±0.21 | 0.41±0.13 | 1.07±0.25 |  | 1.78±0.20^†^ | 1.46±0.33^†^ | 1.87±0.46^*^ |  | 0.91±0.13 | 0.76±0.16 | 0.89±0.09 |  | 1.33±0.03^†^ | 0.51±0.09 | 0.36±0.00^†^ |
| IL-2 | 0.08±0.00 | 0.08±0.00 | 0.20±0.12 |  | 0.17±0.06 | 0.26±0.12 | 0.84±0.13^§^ |  | 0.96±0.06 | 0.92±0.09 | 1.04±0.06 |  | 0.70±0.06^†^ | 0.52±0.03^§^ | 0.61±0.05^§^ |
| IFNγ | 0.34±0.09 | 0.33±0.09 | 0.42±0.12 |  | 1.84±0.04^§^ | 1.58±0.03^§^ | 1.33±0.11^§^ |  | 0.98±0.09 | 0.94±0.13 | 0.98±0.07 |  | 0.94±0.07 | 1.04±0.18 | 1.13±0.05 |
| IL-5 | 0.23±0.07 | 0.22±0.13 | 0.12±0.04 |  | 0.50±0.08^*^ | 1.20±0.14^§^ | 0.73±0.18^§^ |  | 0.09±0.06 | 0.06±0.03 | 0.07±0.02 |  | 0.30±0.02^*^ | 0.95±0.14^§^ | 0.13±0.04 |
| IL-9 | 0.76±0.08 | 0.80±0.05 | 0.88±0.08 |  | 0.98±0.06^*^ | 1.26±0.07^§^ | 1.45±0.08^§^ |  | 1.36±0.13 | 1.34±0.17 | 1.42±0.09 |  | 0.81±0.04^§^ | 0.47±0.08^§^ | 0.60±0.13^§^ |
| IL-13 | 0.65±0.29 | 0.48±0.16 | 0.55±0.14 |  | 0.54±0.09 | 0.85±0.17 | 1.25±0.14^†^ |  | 0.35±0.21 | 0.19±0.12 | 0.67±0.14 |  | 0.07±0.00 | 0.71±0.18^*^ | 0.27±0.20 |
| IL-10 | 0.41±0.00 | 0.63±0.21 | 0.41±0.00 |  | 1.38±0.20^§^ | 1.21±0.19^†^ | 0.67±0.17 |  | 0.11±0.07 | 0.04±0.02 | 0.08±0.03 |  | 1.09±0.03^§^ | 0.75±0.08^§^ | 0.66±0.08^§^ |
| *, †, § - 0.01<p≤0.05, 0.001<p≤0.01 and p≤0.001 respectively for MHV-1 vs control. Interleukin - IL, tumor necrosis factor α - TNFα, Granulocyte Colony-Stimulating Factor – G-CSF, keratinocytes-derived chemokine - KC, Monocyte Chemoattractant Protein-1 - MCP-1, Macrophage Inflammatory Protein - MIP, Regulated upon Activation, Normal T Cell Expressed and Presumably Secreted – RANTES, interferon γ - IFNγ. | | | | | | | | | | | | | | | |

| **Supplemental Table 1. Mean (±SEM) serum and bronchoalveolar lavage (BAL) cytokines levels [log(pg/µl)] with challenge of MHV-1 or its diluent (Control) at Day 2, 5 and 10** | | | | | | | | | | | | | | | |
| --- | --- | --- | --- | --- | --- | --- | --- | --- | --- | --- | --- | --- | --- | --- | --- |
| Cytokines |  | Serum | | | | |  |  |  | BAL | | | | |  |
|  |  | Control |  |  |  | MHV-1 |  |  |  | Control |  |  |  | MHV-1 |  |
|  | 2 | 5 | 10 |  | 2 | 5 | 10 |  | 2 | 5 | 10 |  | 2 | 5 | 10 |
| IL-6 | 0.15±0.08 | 0.10±0.04 | 0.34±0.15 |  | 1.33±0.09^§^ | 1.62±0.17^§^ | 0.75±0.22^*^ |  | 0.21±0.07 | 0.18±0.08 | 0.20±0.04 |  | 2.54±0.05^§^ | 2.35±0.15^§^ | 0.76±0.23^§^ |
| TNFα | 1.11±0.10 | 1.24±0.06 | 1.27±0.12 |  | 1.54±0.05^†^ | 1.51±0.17^*^ | 1.80±0.20^†^ |  | 0.64±0.13 | 0.56±0.10 | 0.71±0.13 |  | 1.69±0.12^§^ | 0.95±0.07^†^ | 1.22±0.07^†^ |
| IL-1β | 0.24±0.04 | 0.24±0.03 | 0.29±0.02 |  | 0.32±0.03 | 0.38±0.03^†^ | 0.42±0.04^*^ |  | 0.07±0.02 | 0.07±0.02 | 0.13±0.01 |  | 0.48±0.05^§^ | 0.10±0.02 | 0.09±0.03 |
| IL-1α | 0.14±0.03 | 0.17±0.06 | 0.27±0.08 |  | 0.38±0.08^*^ | 0.41±0.12^†^ | 0.57±0.13^†^ |  | 0.78±0.05 | 0.77±0.09 | 0.84±0.07 |  | 0.71±0.04 | 0.27±0.02^§^ | 0.22±0.05^§^ |
| G-CSF | 1.20±0.09 | 1.30±0.02 | 1.96±0.20 |  | 3.00±0.04^§^ | 2.86±0.07^§^ | 2.13±0.32 |  | 0.23±0.05 | 0.24±0.08 | 0.51±0.08 |  | 2.78±0.04^§^ | 2.65±0.06^§^ | 1.80±0.17^§^ |
| KC | 1.46±0.04 | 1.44±0.03 | 1.54±0.10 |  | 1.99±0.05^§^ | 2.09±0.11^§^ | 1.75±0.10^*^ |  | 0.91±0.04 | 0.92±0.05 | 1.32±0.06 |  | 2.16±0.05^§^ | 1.93±0.06^§^ | 1.69±0.03^§^ |
| MCP-1 | 1.74±0.09 | 1.73±0.09 | 2.00±0.09 |  | 3.60±0.04^§^ | 3.19±0.09^§^ | 2.65±0.07^§^ |  | 0.80±0.09 | 0.75±0.03 | 0.81±0.07 |  | 4.05±0.03^§^ | 3.74±0.10^§^ | 2.91±0.28^§^ |
| MIP-1α | 0.21±0.03 | 0.25±0.03 | 0.26±0.04 |  | 0.87±0.04^§^ | 0.81±0.05^§^ | 0.74±0.08^§^ |  | 0.10±0.02 | 0.12±0.05 | 0.11±0.02 |  | 2.32±0.13^§^ | 0.85±0.11^§^ | 0.99±0.08^§^ |
| MIP-1β | 1.38±0.13 | 1.32±0.13 | 1.26±0.20 |  | 2.58±0.03^§^ | 2.48±0.05^§^ | 2.20±0.08^§^ |  | 1.35±0.17 | 1.31±0.19 | 1.34±0.10 |  | 3.27±0.07^§^ | 1.99±0.13^§^ | 1.72±0.10 |
| RANTES | 1.78±0.05 | 1.75±0.05 | 1.72±0.04 |  | 2.50±0.03^§^ | 2.32±0.03^§^ | 2.16±0.09^§^ |  | 1.04±0.15 | 0.98±0.08 | 1.26±0.06 |  | 2.50±0.08^§^ | 1.61±0.11^§^ | 1.99±0.11^§^ |
| Eotaxin | 2.88±0.07 | 2.72±0.06 | 2.70±0.03 |  | 2.97±0.03 | 3.00±0.08^†^ | 3.18±0.07^§^ |  | 1.01±0.12 | 1.07±0.06 | 1.23±0.07 |  | 1.98±0.07^§^ | 2.41±0.12^§^ | 2.29±0.14^§^ |
| IL12(p40) | 2.61±0.04 | 2.65±0.05 | 2.56±0.05 |  | 3.23±0.03^§^ | 3.22±0.09^§^ | 2.49±0.08 |  | 0.72±0.09 | 0.76±0.08 | 0.81±0.08 |  | 2.73±0.04^§^ | 2.47±0.09^§^ | 2.21±0.13^§^ |
| IL-17a | 1.50±0.08 | 1.51±0.05 | 1.64±0.10 |  | 1.73±0.05 | 1.56±0.17 | 1.82±0.22 |  | 0.06±0.04 | 0.04±0.02 | 0.04±0.01 |  | 0.14±0.02^†^ | 0.06±0.02 | 0.11±0.03^*^ |
| IL12(p70) | 0.86±0.21 | 0.41±0.13 | 1.07±0.25 |  | 1.78±0.20^†^ | 1.46±0.33^†^ | 1.87±0.46^*^ |  | 0.91±0.13 | 0.76±0.16 | 0.89±0.09 |  | 1.33±0.03^†^ | 0.51±0.09 | 0.36±0.00^†^ |
| IL-2 | 0.08±0.00 | 0.08±0.00 | 0.20±0.12 |  | 0.17±0.06 | 0.26±0.12 | 0.84±0.13^§^ |  | 0.96±0.06 | 0.92±0.09 | 1.04±0.06 |  | 0.70±0.06^†^ | 0.52±0.03^§^ | 0.61±0.05^§^ |
| IFNγ | 0.34±0.09 | 0.33±0.09 | 0.42±0.12 |  | 1.84±0.04^§^ | 1.58±0.03^§^ | 1.33±0.11^§^ |  | 0.98±0.09 | 0.94±0.13 | 0.98±0.07 |  | 0.94±0.07 | 1.04±0.18 | 1.13±0.05 |
| IL-5 | 0.23±0.07 | 0.22±0.13 | 0.12±0.04 |  | 0.50±0.08^*^ | 1.20±0.14^§^ | 0.73±0.18^§^ |  | 0.09±0.06 | 0.06±0.03 | 0.07±0.02 |  | 0.30±0.02^*^ | 0.95±0.14^§^ | 0.13±0.04 |
| IL-9 | 0.76±0.08 | 0.80±0.05 | 0.88±0.08 |  | 0.98±0.06^*^ | 1.26±0.07^§^ | 1.45±0.08^§^ |  | 1.36±0.13 | 1.34±0.17 | 1.42±0.09 |  | 0.81±0.04^§^ | 0.47±0.08^§^ | 0.60±0.13^§^ |
| IL-13 | 0.65±0.29 | 0.48±0.16 | 0.55±0.14 |  | 0.54±0.09 | 0.85±0.17 | 1.25±0.14^†^ |  | 0.35±0.21 | 0.19±0.12 | 0.67±0.14 |  | 0.07±0.00 | 0.71±0.18^*^ | 0.27±0.20 |
| IL-10 | 0.41±0.00 | 0.63±0.21 | 0.41±0.00 |  | 1.38±0.20^§^ | 1.21±0.19^†^ | 0.67±0.17 |  | 0.11±0.07 | 0.04±0.02 | 0.08±0.03 |  | 1.09±0.03^§^ | 0.75±0.08^§^ | 0.66±0.08^§^ |
| *, †, § - 0.01<p≤0.05, 0.001<p≤0.01 and p≤0.001 respectively for MHV-1 vs control. Interleukin - IL, tumor necrosis factor α - TNFα, Granulocyte Colony-Stimulating Factor – G-CSF, keratinocytes-derived chemokine - KC, Monocyte Chemoattractant Protein-1 - MCP-1, Macrophage Inflammatory Protein - MIP, Regulated upon Activation, Normal T Cell Expressed and Presumably Secreted – RANTES, interferon γ - IFNγ. | | | | | | | | | | | | | | | |
