# Supplementary material for: Anti-PD-L1 therapy altered inflammation but not survival in a lethal murine hepatitis virus-1 pneumonia model
Source: Front Immunol. 2024 Jan 8;14:1308358. doi: 10.3389/fimmu.2023.1308358 (PMC10801642; doi:10.3389/fimmu.2023.1308358)
Supplement: Supplementary file 3 [file Table_2.docx]

| **Supplemental Table 2**. The overall serum protein expression [log2(fold change)(log2(FC)] in MHV-1 compared to diluent challenged animals across all treatments (virus effect) and in PD-L1mAb compared to isomAb treated animals across all challenges (treatment effect) at Day 2 and 5 | | | | | | | | | | | |
| --- | --- | --- | --- | --- | --- | --- | --- | --- | --- | --- | --- |
| Virus effect at Day 2 | | | Virus effect at Day 5 | | | Treatment effect at Day 2 | | | Treatment effect at Day 5 | | |
| Protein | log2(FC) ±SE | FDR* | Protein | log2(FC) ±SE | FDR* | Protein | log2(FC) ± SE | FDR* | Protein | log2(FC) ± SE | FDR* |
| Ccl2 | 4.181±0.101 | <0.001 | Cntn4 | -1.295±0.067 | <0.001 | Tnfrsf11b# | 0.625±0.080 | <0.001 | Matn2 | 0.912±0.101 | <0.001 |
| Ccl3 | 2.138±0.110 | <0.001 | Wfikkn2 | 1.425±0.090 | <0.001 | Sez6l2 | 0.212±0.036 | <0.001 | Sez6l2 | 0.479±0.063 | <0.001 |
| Cxcl9 | 4.056±0.214 | <0.001 | Ccl2 | 3.995±0.257 | <0.001 | Igsf3 | 0.300±0.058 | <0.001 | Vegfd | 0.394±0.070 | <0.001 |
| Il6 | 1.436±0.087 | <0.001 | Tnf | 1.776±0.121 | <0.001 | Ccl3# | 0.527±0.111 | 0.001 | Ccl3 | 0.797±0.150 | <0.001 |
| Il10 | 2.461±0.126 | <0.001 | Tnfrsf11b | 2.097±0.148 | <0.001 | Matn2 | 0.474±0.106 | 0.001 | Igsf3 | 0.374±0.072 | <0.001 |
| Lgmn | 0.754±0.051 | <0.001 | Ccl3 | 2.074±0.149 | <0.001 | Gfra1 | 0.292±0.065 | 0.001 | Tnfsf12 | 0.564±0.120 | 0.001 |
| Dll1 | 0.833±0.057 | <0.001 | Dll1 | 0.978±0.073 | <0.001 | Adam23 | 0.350±0.078 | 0.001 | Flrt2 | 0.324±0.069 | 0.001 |
| Cxcl1 | 1.649±0.116 | <0.001 | Cyr61 | -0.875±0.071 | <0.001 | Cntn4 | -0.174±0.042 | 0.003 | Tnfrsf11b | 0.590±0.150 | 0.005 |
| Cyr61 | -0.815±0.068 | <0.001 | Il10 | 2.197±0.180 | <0.001 | Tnfsf12# | 0.295±0.075 | 0.004 | Tnr | 0.226±0.059 | 0.006 |
| Wfikkn2 | 0.553±0.046 | <0.001 | Clmp | 1.070±0.087 | <0.001 | Qdpr | -0.668±0.184 | 0.009 | Il5 | -1.072±0.283 | 0.006 |
| Cntn4 | -0.488±0.042 | <0.001 | Lgmn | 0.830±0.070 | <0.001 | Csf2 | -0.774±0.222 | 0.012 | Tnf | 0.440±0.123 | 0.010 |
| Tnf | 1.114±0.097 | <0.001 | Cxcl1 | 2.200±0.194 | <0.001 | Clmp | 0.146±0.050 | 0.046 | Wisp1 | -0.255±0.073 | 0.011 |
| Wisp1 | -0.649±0.065 | <0.001 | Parp1 | 2.286±0.212 | <0.001 | Cxcl9# | 0.620±0.215 | 0.049 | Dll1 | 0.258±0.074 | 0.011 |
| Csf2 | 1.915±0.221 | <0.001 | Cxcl9 | 3.367±0.353 | <0.001 | Tnr | 0.158±0.056 | 0.055 | Cxcl9 | 1.199±0.357 | 0.014 |
| Tnfrsf11b | 0.676±0.079 | <0.001 | Fas | 1.393±0.150 | <0.001 | Tpp1 | 0.169±0.061 | 0.055 | Cdh6 | 0.294±0.089 | 0.015 |
| Map2k6 | -1.206±0.142 | <0.001 | Tpp1 | 0.492±0.053 | <0.001 | Dlk1 | -0.205±0.076 | 0.065 | Notch3 | 0.181±0.055 | 0.016 |
| Fst | 1.247±0.148 | <0.001 | Csf2 | 1.640±0.178 | <0.001 | Tgfa | -0.339±0.133 | 0.085 | Ccl5 | 1.096±0.342 | 0.017 |
| Nadk | 1.007±0.123 | <0.001 | Il6 | 2.009±0.219 | <0.001 | Wfikkn2 | 0.115±0.047 | 0.096 | Gfra1 | 0.476±0.150 | 0.017 |
| Fas | 0.581±0.071 | <0.001 | Tgfbr3 | 0.421±0.047 | <0.001 | Flrt2 | 0.156±0.064 | 0.101 | Plxna4 | -0.705±0.251 | 0.042 |
| Parp1 | 1.105±0.148 | <0.001 | Cntn1 | -0.631±0.070 | <0.001 | Dll1 | 0.139±0.058 | 0.101 | Clstn2 | 0.337±0.121 | 0.042 |
| Clstn2 | -0.597±0.082 | <0.001 | Itgb6 | 0.679±0.091 | <0.001 | Il17f | -0.367±0.156 | 0.106 | Cntn1 | 0.195±0.071 | 0.045 |
| Epo | -1.117±0.157 | <0.001 | Cpe | -0.640±0.087 | <0.001 | Vegfd | 0.128±0.054 | 0.106 | Adam23 | 0.345±0.134 | 0.061 |
| Plxna4 | -1.341±0.195 | <0.001 | Tnfsf12 | 0.854±0.118 | <0.001 | Ddah1 | -0.292±0.127 | 0.106 | Qdpr | -0.499±0.194 | 0.061 |
| Casp3 | -0.934±0.144 | <0.001 | Eda2r | 1.026±0.144 | <0.001 | Il17a | -0.466±0.202 | 0.106 | Vsig2 | 0.459±0.183 | 0.069 |
| Sez6l2 | -0.213±0.036 | <0.001 | Flrt2 | -0.449±0.068 | <0.001 | Cpe | -0.170±0.075 | 0.112 | Il1a | 0.397±0.163 | 0.077 |
| Cntn1 | -0.169±0.029 | <0.001 | Eno2 | 1.695±0.263 | <0.001 | Nadk | -0.276±0.124 | 0.114 | Map2k6 | -0.359±0.149 | 0.077 |
| Flrt2 | -0.370±0.064 | <0.001 | Apbb1ip | 0.833±0.136 | <0.001 | Cyr61 | 0.149±0.068 | 0.117 | Il17f | -0.500±0.212 | 0.084 |
| Vegfd | 0.301±0.054 | <0.001 | Gdnf | 0.628±0.104 | <0.001 | Cxcl1 | 0.256±0.117 | 0.117 | Riox2 | 0.515±0.219 | 0.084 |
| Il1a | -0.680±0.124 | <0.001 | Fstl3 | 0.539±0.090 | <0.001 | Tgfbr3 | 0.101±0.046 | 0.117 | Crim1 | 0.180±0.078 | 0.090 |
| Tpp1 | 0.318±0.061 | <0.001 | Riox2 | 1.279±0.216 | <0.001 | Riox2 | 0.353±0.169 | 0.136 | Tpp1 | 0.114±0.054 | 0.133 |
| Hgf | -0.915±0.177 | <0.001 | Il5 | 1.639±0.280 | <0.001 | Lpl | -0.198±0.108 | 0.223 | Epo | -0.743±0.355 | 0.133 |
| Cdh6 | -0.318±0.063 | <0.001 | Tnr | 0.329±0.058 | <0.001 | Yes1 | -0.221±0.127 | 0.261 | Fas | 0.312±0.152 | 0.139 |
| Dlk1 | -0.381±0.076 | <0.001 | Yes1 | 1.257±0.223 | <0.001 | Plxna4 | -0.332±0.196 | 0.270 | Ddah1 | -0.408±0.210 | 0.167 |
| Tnr | -0.272±0.056 | <0.001 | Dctn2 | 1.229±0.218 | <0.001 | Pak4 | 0.166±0.098 | 0.270 | Il10 | 0.353±0.182 | 0.167 |
| Crim1 | -0.266±0.062 | <0.001 | Ntf3 | 0.590±0.105 | <0.001 | Cant1 | 0.112±0.067 | 0.272 | Casp3 | -0.275±0.149 | 0.195 |
| Vsig2 | -0.428±0.104 | <0.001 | Mia | -0.421±0.075 | <0.001 | Vsig2 | 0.161±0.105 | 0.343 | Prdx5 | -0.327±0.181 | 0.198 |
| Eda2r | 0.470±0.116 | <0.001 | Fst | 0.622±0.115 | <0.001 | Fstl3 | -0.076±0.050 | 0.350 | Snap29 | -0.236±0.131 | 0.198 |
| Il1b | 0.251±0.064 | 0.001 | Nadk | 0.975±0.180 | <0.001 | Epo | -0.236±0.158 | 0.350 | Eno2 | 0.477±0.266 | 0.198 |
| Notch3 | -0.176±0.046 | 0.001 | Clstn2 | -0.645±0.120 | <0.001 | Lgmn | 0.076±0.051 | 0.354 | Cntn4 | 0.121±0.067 | 0.198 |
| Ppp1r2 | 0.445±0.118 | 0.002 | Ghrl | -0.747±0.143 | <0.001 | Itgb1bp2 | 0.139±0.095 | 0.355 | Acvrl1 | -0.166±0.095 | 0.201 |
| Epcam | -0.322±0.086 | 0.002 | Igsf3 | 0.357±0.071 | <0.001 | S100a4 | 0.186±0.130 | 0.364 | Cyr61 | 0.126±0.072 | 0.201 |
| S100a4 | -0.480±0.130 | 0.002 | Tnfrsf12a | 0.546±0.111 | <0.001 | Apbb1ip | 0.151±0.107 | 0.364 | Nadk | -0.315±0.182 | 0.206 |
| Cant1 | -0.245±0.066 | 0.002 | Map2k6 | -0.708±0.147 | <0.001 | Notch3 | 0.064±0.046 | 0.371 | Plin1 | -0.418±0.255 | 0.238 |
| Ghrl | -0.455±0.127 | 0.002 | Prdx5 | 0.842±0.179 | <0.001 | Tnni3 | 0.801±0.586 | 0.376 | Ntf3 | 0.159±0.106 | 0.300 |
| Tnfrsf12a | -0.257±0.077 | 0.004 | Ca13 | 0.744±0.158 | <0.001 | Pdgfb | 0.170±0.125 | 0.376 | Parp1 | 0.305±0.214 | 0.337 |
| Rgma | -0.177±0.053 | 0.005 | Cdh6 | -0.406±0.087 | <0.001 | Erbb4 | 0.089±0.067 | 0.379 | Gdnf | 0.146±0.106 | 0.355 |
| Cpe | -0.237±0.075 | 0.006 | Rgma | -0.227±0.051 | <0.001 | Fli1 | 0.206±0.155 | 0.380 | Tgfbr3 | 0.063±0.047 | 0.372 |
| Erbb4 | 0.205±0.066 | 0.008 | Qdpr | 0.772±0.192 | 0.001 | Cdh6 | 0.079±0.063 | 0.419 | Pla2g4a | -0.215±0.174 | 0.424 |
| Adam23 | 0.231±0.078 | 0.010 | Ppp1r2 | 0.481±0.121 | 0.001 | Clstn2 | 0.100±0.083 | 0.437 | S100a4 | 0.139±0.112 | 0.424 |
| Tgfb1 | -0.459±0.165 | 0.016 | Matn2 | 0.399±0.100 | 0.001 | Map2k6 | -0.162±0.143 | 0.486 | Il6 | -0.270±0.222 | 0.427 |
| Ccl5 | 0.864±0.334 | 0.026 | Gfra1 | 0.587±0.148 | 0.001 | Ccl5 | -0.375±0.336 | 0.491 | Il17a | -0.284±0.240 | 0.436 |
| Ahr | -0.169±0.067 | 0.029 | Il1a | 0.630±0.161 | 0.001 | Eno2 | 0.069±0.068 | 0.558 | Cant1 | 0.095±0.081 | 0.436 |
| Ca13 | -0.251±0.105 | 0.039 | Acvrl1 | 0.362±0.094 | 0.001 | Il23r | -0.060±0.062 | 0.583 | Pak4 | 0.256±0.219 | 0.436 |
| Igsf3 | -0.133±0.058 | 0.046 | Epo | 1.358±0.351 | 0.001 | Itgb6 | 0.113±0.120 | 0.604 | Hgf | -0.188±0.169 | 0.465 |
| Pdgfb | -0.279±0.125 | 0.054 | Vegfd | 0.268±0.069 | 0.001 | Fas | 0.066±0.072 | 0.610 | Itgb6 | -0.101±0.092 | 0.465 |
| Il17a | -0.432±0.201 | 0.064 | Notch3 | -0.206±0.055 | 0.001 | Eda2r | -0.105±0.117 | 0.610 | Gcg | -0.224±0.207 | 0.472 |
| Snap29 | 0.276±0.140 | 0.091 | Plxna4 | -0.919±0.248 | 0.001 | Il1a | 0.111±0.125 | 0.610 | Fstl3 | -0.093±0.091 | 0.502 |
| Mia | -0.202±0.106 | 0.105 | Sez6l2 | -0.208±0.062 | 0.003 | Hgf | 0.154±0.178 | 0.625 | Tgfa | -0.156±0.154 | 0.502 |
| Matn2 | -0.187±0.105 | 0.131 | S100a4 | -0.367±0.111 | 0.004 | Ca13 | -0.083±0.105 | 0.680 | Epcam | -0.058±0.062 | 0.558 |
| Gfra1 | -0.111±0.065 | 0.145 | Il23r | 0.223±0.069 | 0.004 | Gdnf | -0.046±0.061 | 0.701 | Pdgfb | -0.163±0.221 | 0.715 |
| Tgfa | -0.215±0.132 | 0.172 | Adam23 | 0.428±0.132 | 0.004 | Rgma | -0.040±0.054 | 0.701 | Apbb1ip | 0.100±0.138 | 0.715 |
| Pla2g4a | -0.185±0.116 | 0.179 | Ddah1 | 0.648±0.208 | 0.006 | Fst | -0.102±0.149 | 0.740 | Fli1 | 0.095±0.138 | 0.724 |
| Eno2 | 0.101±0.068 | 0.214 | Tgfa | -0.438±0.152 | 0.010 | Prdx5 | -0.093±0.139 | 0.740 | Kitlg | 0.056±0.082 | 0.724 |
| Tnni3 | 0.833±0.583 | 0.234 | Casp3 | -0.423±0.147 | 0.010 | Wisp1 | -0.042±0.065 | 0.740 | Tnfrsf12a | -0.075±0.112 | 0.734 |
| Apbb1ip | 0.148±0.106 | 0.244 | Vsig2 | -0.507±0.181 | 0.013 | Ahr | 0.043±0.067 | 0.740 | Tnni3 | 0.314±0.553 | 0.781 |
| Ddah1 | 0.175±0.126 | 0.244 | Ahr | 0.231±0.089 | 0.019 | Pla2g4a | 0.069±0.117 | 0.777 | Yes1 | -0.126±0.225 | 0.781 |
| Acvrl1 | 0.147±0.109 | 0.253 | Ccl5 | 0.881±0.338 | 0.019 | Tnfrsf12a | -0.044±0.077 | 0.783 | Ccl2 | -0.142±0.260 | 0.781 |
| Pak4 | -0.131±0.097 | 0.253 | Pla2g4a | 0.447±0.171 | 0.019 | Ghrl | -0.072±0.128 | 0.783 | Il1b | 0.046±0.086 | 0.781 |
| Il23r | 0.079±0.061 | 0.270 | Wisp1 | -0.158±0.072 | 0.048 | Ppp1r2 | 0.065±0.119 | 0.787 | Csf2 | 0.094±0.181 | 0.781 |
| Yes1 | 0.163±0.126 | 0.270 | Itgb1bp2 | -0.205±0.098 | 0.060 | Tnf | -0.051±0.097 | 0.793 | Cxcl1 | -0.100±0.197 | 0.781 |
| Clmp | 0.063±0.049 | 0.277 | Snap29 | 0.259±0.129 | 0.070 | Crim1 | 0.030±0.063 | 0.819 | Mia | -0.038±0.076 | 0.781 |
| Tgfbr3 | -0.058±0.046 | 0.2773 | Crim1 | 0.155±0.077 | 0.070 | Casp3 | -0.063±0.145 | 0.848 | Itgb1bp2 | -0.049±0.099 | 0.781 |
| Itgb1bp2 | -0.117±0.095 | 0.286 | Lpl | 0.379±0.193 | 0.074 | Parp1 | 0.064±0.149 | 0.848 | Ppp1r2 | 0.059±0.122 | 0.781 |
| Ntf3 | -0.215±0.182 | 0.305 | Tnni3 | 1.019±0.547 | 0.090 | Acvrl1 | -0.039±0.109 | 0.886 | Foxo1 | -0.031±0.065 | 0.781 |
| Il17f | -0.168±0.156 | 0.354 | Pdgfb | -0.400±0.219 | 0.095 | Ccl20 | 0.097±0.271 | 0.886 | Lpl | -0.093±0.195 | 0.781 |
| Foxo1 | -0.057±0.056 | 0.378 | Gcg | -0.363±0.205 | 0.105 | Il1b | 0.021±0.064 | 0.904 | Ccl20 | -0.093±0.222 | 0.810 |
| Fli1 | -0.152±0.154 | 0.396 | Cant1 | -0.139±0.080 | 0.109 | Il6 | -0.021±0.088 | 0.952 | Erbb4 | 0.036±0.087 | 0.810 |
| Gcg | 0.155±0.164 | 0.414 | Dlk1 | -0.118±0.068 | 0.109 | Epcam | -0.019±0.086 | 0.952 | Ca13 | -0.063±0.160 | 0.810 |
| Plin1 | 0.178±0.206 | 0.460 | Plin1 | -0.429±0.252 | 0.115 | Ccl2 | -0.022±0.101 | 0.952 | Rgma | 0.020±0.051 | 0.810 |
| Dctn2 | -0.129±0.160 | 0.491 | Il1b | 0.143±0.085 | 0.121 | Kitlg | 0.013±0.064 | 0.952 | Lgmn | 0.027±0.071 | 0.810 |
| Il5 | -0.105±0.162 | 0.593 | Pak4 | 0.344±0.217 | 0.139 | Snap29 | 0.024±0.140 | 0.952 | Axin1 | -0.036±0.102 | 0.820 |
| Prdx5 | 0.078±0.138 | 0.647 | Epcam | -0.084±0.061 | 0.201 | Ntf3 | -0.030±0.182 | 0.952 | Fst | 0.038±0.116 | 0.835 |
| Itgb6 | -0.065±0.119 | 0.653 | Fli1 | 0.160±0.136 | 0.277 | Il10 | 0.020±0.127 | 0.952 | Eda2r | -0.044±0.146 | 0.850 |
| Lpl | -0.055±0.107 | 0.667 | Hgf | -0.188±0.167 | 0.295 | Mia | 0.017±0.107 | 0.952 | Il23r | -0.018±0.069 | 0.859 |
| Gdnf | 0.029±0.060 | 0.688 | Tgfb1 | -0.172±0.167 | 0.340 | Tgfb1 | -0.025±0.165 | 0.952 | Dctn2 | -0.058±0.221 | 0.859 |
| Qdpr | 0.085±0.183 | 0.692 | Ccl20 | 0.218±0.219 | 0.350 | Cntn1 | 0.003±0.029 | 0.976 | Wfikkn2 | -0.021±0.091 | 0.878 |
| Fstl3 | 0.017±0.050 | 0.780 | Axin1 | 0.085±0.100 | 0.427 | Foxo1 | -0.004±0.056 | 0.976 | Cpe | -0.019±0.088 | 0.882 |
| Ccl20 | -0.073±0.270 | 0.8239 | Erbb4 | 0.064±0.086 | 0.485 | Dctn2 | 0.011±0.161 | 0.976 | Ghrl | -0.027±0.145 | 0.893 |
| Kitlg | -0.008±0.064 | 0.929 | Foxo1 | 0.042±0.064 | 0.531 | Plin1 | -0.013±0.207 | 0.976 | Clmp | 0.015±0.089 | 0.896 |
| Axin1 | 0.003±0.083 | 0.992 | Il17f | 0.116±0.209 | 0.596 | Gcg | 0.009±0.164 | 0.976 | Dlk1 | -0.007±0.068 | 0.944 |
| Tnfsf12 | 0.001±0.075 | 0.992 | Il17a | 0.010±0.237 | 0.695 | Il5 | -0.006±0.163 | 0.981 | Ahr | 0.007±0.090 | 0.948 |
| Riox2 | 0.002±0.168 | 0.992 | Kitlg | -0.010±0.080 | 0.923 | Axin1 | -0.002±0.083 | 0.981 | Tgfb1 | 0.001±0.170 | 0.993 |
| *- FDR: false discovery rate, #- Proteins demonstrating an interaction effect with the virus cannot be fully explained by the treatment alone, identified as increased (red) or decreased (blue) compared to MHV-1 diluent or ISO-mAb at each timepoint. Grayed cells indicate proteins common to both 2d and 5d. | | | | | | | | | | | |
