# Supplementary material for: Anti-PD-L1 therapy altered inflammation but not survival in a lethal murine hepatitis virus-1 pneumonia model
Source: Front Immunol. 2024 Jan 8;14:1308358. doi: 10.3389/fimmu.2023.1308358 (PMC10801642; doi:10.3389/fimmu.2023.1308358)
Supplement: Supplementary file 4 [file Table_3.docx]

| **Supplemental Table 3**. The overall serum protein expression [log2(fold change)(log2(FC)] in treatment with PD-L1mAb over isomAb in MHV-1 or diluent challenged animals at Day 2 or 5 | | | | | | | | | | | | |
| --- | --- | --- | --- | --- | --- | --- | --- | --- | --- | --- | --- | --- |
| Diluent at Day 2 | | | Diluent at Day 5 | | | MHV-1 at Day 2 | | | MHV-1 at Day 5 | | | |
| Protein | log2(FC)±SE | FDR* | Protein | log2(FC) ±SE | FDR* | Protein | log2(FC) ±SE | FDR* | Protein | log2(FC) ±SE | FDR* |  |
| Tnfrsf11b | 1.009±0.114 | <0.001 | Ccl3 | 1.093±0.204 | 0.001 | Tnfsf12 | 0.575±0.106 | 0.001 | Matn2 | 1.237±0.150 | <0.001 |  |
| Ccl3 | 1.005±0.157 | <0.001 | Tnfrsf11b | 0.996±0.203 | 0.001 | Matn2 | 0.644±0.149 | 0.006 | Sez6l2 | 0.646±0.093 | <0.001 |  |
| Cxcl9 | 1.604±0.305 | <0.001 | Matn2 | 0.588±0.137 | 0.005 | Gfra1 | 0.365±0.092 | 0.011 | Tnfsf12 | 0.947±0.177 | <0.001 |  |
| Sez6l2 | 0.235±0.051 | 0.001 | Nadk | -0.972±0.247 | 0.010 | Csf2 | -1.215±0.313 | 0.011 | Flrt2 | 0.519±0.102 | <0.001 |  |
| Cntn4 | -0.235±0.060 | 0.007 | Sez6l2 | 0.311±0.085 | 0.017 | Sez6l2 | 0.188±0.051 | 0.014 | Il5 | -2.124±0.419 | <0.001 |  |
| Igsf3 | 0.308±0.082 | 0.010 | Cxcl9 | 1.705±0.484 | 0.018 | Igsf3 | 0.292±0.081 | 0.017 | Vegfd | 0.488±0.104 | 0.001 |  |
| Adam23 | 0.401±0.111 | 0.013 | Ddah1 | -0.993±0.285 | 0.018 | Cxcl1 | 0.496±0.165 | 0.065 | Igsf3 | 0.468±0.107 | 0.002 |  |
| Dll1 | 0.252±0.082 | 0.047 | Qdpr | -0.914±0.263 | 0.018 | Qdpr | -0.764±0.260 | 0.068 | Cntn1 | 0.404±0.105 | 0.007 |  |
| Fas | 0.284±0.102 | 0.087 | Vegfd | 0.300±0.095 | 0.037 | Tpp1 | 0.246±0.086 | 0.073 | Wisp1 | -0.409±0.107 | 0.007 |  |
| Vegfd | 0.213±0.077 | 0.087 | Il1a | 0.658±0.221 | 0.051 | Adam23 | 0.299±0.110 | 0.096 | Tnr | 0.315±0.087 | 0.009 |  |
| Vsig2 | 0.389±0.148 | 0.109 | Ccl5 | 1.337±0.463 | 0.058 | Tgfbr3 | 0.173±0.065 | 0.102 | Gfra1 | 0.766±0.221 | 0.013 |  |
| Cpe | -0.266±0.107 | 0.137 | Igsf3 | 0.280±0.098 | 0.058 | Tnr | 0.179±0.079 | 0.233 | Cdh6 | 0.452±0.131 | 0.013 |  |
| Lpl | -0.371±0.153 | 0.146 | Il17f | -0.813±0.287 | 0.058 | Tnfrsf11b | 0.240±0.113 | 0.290 | Map2k6 | -0.736±0.220 | 0.015 |  |
| Gfra1 | 0.218±0.092 | 0.156 | Dll1 | 0.277±0.100 | 0.062 | S100a4 | 0.379±0.184 | 0.292 | Riox2 | 1.061±0.324 | 0.017 |  |
| Dlk1 | -0.249±0.108 | 0.172 | Adam23 | 0.488±0.181 | 0.071 | Flrt2 | 0.186±0.090 | 0.292 | Cntn4 | 0.317±0.100 | 0.021 |  |
| Qdpr | -0.572±0.261 | 0.203 | Notch3 | 0.198±0.075 | 0.074 | Clmp | 0.141±0.070 | 0.304 | Tnf | 0.547±0.182 | 0.030 |  |
| Clmp | 0.151±0.070 | 0.214 | Tgfa | -0.529±0.208 | 0.089 | Tgfa | -0.366±0.188 | 0.321 | Casp3 | -0.627±0.220 | 0.042 |  |
| Erbb4 | 0.195±0.095 | 0.241 | Cpe | -0.281±0.120 | 0.131 | Cntn4 | -0.113±0.059 | 0.327 | Plxna4 | -0.968±0.371 | 0.071 |  |
| Matn2 | 0.305±0.150 | 0.241 | Lpl | -0.616±0.265 | 0.131 | Tnf | -0.259±0.137 | 0.327 | Eno2 | 0.889±0.393 | 0.146 |  |
| Nadk | -0.344±0.175 | 0.267 | Vsig2 | 0.506±0.248 | 0.226 | Wfikkn2 | 0.117±0.066 | 0.363 | Ccl3 | 0.501±0.222 | 0.146 |  |
| Il17f | -0.420±0.222 | 0.292 | Cant1 | 0.220±0.109 | 0.226 | Cyr61 | 0.170±0.096 | 0.363 | Crim1 | 0.255±0.116 | 0.156 |  |
| Epo | -0.404±0.224 | 0.324 | Tnf | 0.334±0.166 | 0.226 | Pdgfb | 0.308±0.177 | 0.363 | Dll1 | 0.238±0.109 | 0.156 |  |
| Itgb1bp2 | 0.238±0.135 | 0.324 | Clstn2 | 0.305±0.164 | 0.290 | Ddah1 | -0.312±0.179 | 0.363 | Il10 | 0.581±0.269 | 0.156 |  |
| Notch3 | 0.113±0.065 | 0.324 | Il1b | 0.206±0.117 | 0.342 | Pak4 | 0.230±0.138 | 0.390 | Fas | 0.471±0.225 | 0.164 |  |
| Plxna4 | -0.478±0.277 | 0.324 | Tnr | 0.137±0.079 | 0.343 | Il17a | -0.474±0.285 | 0.390 | Il6 | -0.685±0.328 | 0.164 |  |
| Wfikkn2 | 0.113±0.066 | 0.324 | Il17a | -0.557±0.325 | 0.343 | Fas | -0.153±0.101 | 0.461 | Clstn2 | 0.369±0.179 | 0.164 |  |
| Tnr | 0.136±0.080 | 0.324 | Epo | -0.748±0.481 | 0.443 | Itgb6 | 0.255±0.169 | 0.461 | S100a4 | 0.341±0.166 | 0.164 |  |
| Riox2 | 0.407±0.240 | 0.324 | Ntf3 | 0.210±0.144 | 0.508 | Ccl2 | -0.214±0.143 | 0.461 | Notch3 | 0.164±0.082 | 0.182 |  |
| Tgfa | -0.312±0.189 | 0.342 | Acvrl1 | -0.179±0.128 | 0.548 | Dlk1 | -0.161±0.108 | 0.461 | Tpp1 | 0.157±0.080 | 0.189 |  |
| Il17a | -0.457±0.287 | 0.370 | Flrt2 | 0.129±0.094 | 0.549 | Il17f | -0.315±0.220 | 0.500 | Pla2g4a | -0.487±0.257 | 0.208 |  |
| Ddah1 | -0.273±0.180 | 0.398 | Kitlg | 0.144±0.111 | 0.584 | Lgmn | 0.099±0.073 | 0.534 | Cpe | 0.243±0.131 | 0.216 |  |
| Yes1 | -0.268±0.180 | 0.398 | Plxna4 | -0.441±0.339 | 0.584 | Riox2 | 0.300±0.238 | 0.614 | Itgb6 | -0.241±0.136 | 0.246 |  |
| Clstn2 | 0.174±0.117 | 0.398 | Ghrl | -0.247±0.196 | 0.604 | Eda2r | -0.205±0.164 | 0.614 | Parp1 | 0.538±0.317 | 0.266 |  |
| Ccl5 | -0.706±0.476 | 0.398 | Mia | -0.123±0.103 | 0.651 | Tnni3 | 0.995±0.826 | 0.614 | Ppp1r2 | 0.305±0.181 | 0.266 |  |
| Wisp1 | -0.134±0.092 | 0.412 | Rgma | -0.079±0.070 | 0.659 | Nadk | -0.209±0.174 | 0.614 | Ccl5 | 0.855±0.506 | 0.266 |  |
| Flrt2 | 0.125±0.091 | 0.455 | Ppp1r2 | -0.187±0.165 | 0.659 | Cxcl9 | -0.363±0.304 | 0.614 | Cxcl1 | -0.468±0.291 | 0.301 |  |
| Cyr61 | 0.128±0.096 | 0.467 | Cdh6 | 0.135±0.120 | 0.659 | Cant1 | 0.110±0.094 | 0.622 | Prdx5 | -0.424±0.267 | 0.305 |  |
| Fstl3 | -0.095±0.071 | 0.467 | Tnfsf12 | 0.180±0.162 | 0.659 | Tnfrsf12a | -0.125±0.109 | 0.630 | Cyr61 | 0.166±0.106 | 0.305 |  |
| Apbb1ip | 0.195±0.152 | 0.488 | Plin1 | -0.380±0.345 | 0.659 | Cdh6 | 0.100±0.089 | 0.630 | Rgma | 0.119±0.076 | 0.305 |  |
| Cant1 | 0.113±0.095 | 0.551 | Wisp1 | -0.100±0.098 | 0.682 | Gdnf | -0.095±0.085 | 0.630 | Snap29 | -0.295±0.194 | 0.313 |  |
| Ccl2 | 0.170±0.144 | 0.551 | Cxcl1 | 0.268±0.266 | 0.682 | Hgf | 0.269±0.252 | 0.656 | Vsig2 | 0.411±0.271 | 0.313 |  |
| Il1b | 0.106±0.091 | 0.553 | Crim1 | 0.106±0.106 | 0.682 | Il23r | -0.092±0.087 | 0.656 | Lpl | 0.429±0.289 | 0.324 |  |
| Tnf | 0.157±0.138 | 0.561 | Clmp | 0.120±0.120 | 0.682 | Map2k6 | -0.201±0.202 | 0.684 | Epo | -0.738±0.525 | 0.364 |  |
| Tpp1 | 0.092±0.086 | 0.609 | Snap29 | -0.177±0.177 | 0.682 | Pla2g4a | 0.164±0.165 | 0.684 | Gcg | -0.416±0.306 | 0.385 |  |
| Csf2 | -0.333±0.315 | 0.609 | Tpp1 | 0.071±0.073 | 0.682 | Yes1 | -0.174±0.179 | 0.690 | Fstl3 | -0.178±0.134 | 0.391 |  |
| Fli1 | 0.227±0.220 | 0.620 | Gdnf | 0.136±0.143 | 0.682 | Ccl20 | 0.365±0.382 | 0.692 | Hgf | -0.329±0.250 | 0.391 |  |
| Dctn2 | 0.228±0.228 | 0.633 | Ccl20 | -0.282±0.300 | 0.682 | Fst | -0.196±0.210 | 0.692 | Cxcl9 | 0.693±0.529 | 0.391 |  |
| Eno2 | 0.080±0.096 | 0.795 | Prdx5 | -0.230±0.245 | 0.682 | Dctn2 | -0.207±0.227 | 0.692 | Pak4 | 0.413±0.324 | 0.400 |  |
| Ghrl | -0.138±0.181 | 0.816 | Gfra1 | 0.186±0.203 | 0.686 | Ntf3 | -0.235±0.257 | 0.692 | Nadk | 0.343±0.269 | 0.400 |  |
| Tnni3 | 0.607±0.830 | 0.816 | Tgfb1 | 0.207±0.230 | 0.688 | Fli1 | 0.184±0.219 | 0.744 | Pdgfb | -0.408±0.328 | 0.409 |  |
| Rgma | -0.056±0.076 | 0.816 | Cyr61 | 0.086±0.097 | 0.691 | Ppp1r2 | 0.135±0.167 | 0.744 | Plin1 | -0.455±0.377 | 0.426 |  |
| Pak4 | 0.101±0.139 | 0.816 | Eda2r | -0.171±0.198 | 0.696 | Fstl3 | -0.057±0.071 | 0.744 | Tnni3 | 0.975±0.818 | 0.429 |  |
| Il1a | 0.127±0.177 | 0.816 | Csf2 | 0.209±0.245 | 0.696 | Prdx5 | -0.157±0.196 | 0.744 | Acvrl1 | -0.154±0.140 | 0.487 |  |
| Lgmn | 0.052±0.073 | 0.816 | Cntn4 | -0.076±0.091 | 0.703 | Ca13 | -0.113±0.149 | 0.761 | Tgfbr3 | 0.075±0.070 | 0.492 |  |
| Ntf3 | 0.175±0.259 | 0.842 | Tgfbr3 | 0.051±0.064 | 0.721 | Mia | 0.111±0.151 | 0.761 | Fst | 0.178±0.171 | 0.516 |  |
| Cdh6 | 0.059±0.090 | 0.845 | Fas | 0.153±0.206 | 0.758 | Apbb1ip | 0.108±0.151 | 0.761 | Adam23 | 0.202±0.198 | 0.518 |  |
| Kitlg | 0.056±0.091 | 0.866 | Yes1 | -0.217±0.305 | 0.775 | Epcam | -0.086±0.121 | 0.761 | Gdnf | 0.156±0.156 | 0.521 |  |
| Map2k6 | -0.124±0.203 | 0.866 | Fli1 | 0.130±0.187 | 0.775 | Il1b | -0.064±0.091 | 0.761 | Erbb4 | 0.128±0.129 | 0.521 |  |
| Parp1 | 0.110±0.211 | 0.941 | Lgmn | 0.066±0.096 | 0.775 | Cpe | -0.074±0.106 | 0.761 | Tgfa | 0.216±0.227 | 0.544 |  |
| Mia | -0.077±0.151 | 0.941 | Dlk1 | 0.062±0.093 | 0.778 | Plxna4 | -0.187±0.276 | 0.773 | Ghrl | 0.194±0.214 | 0.568 |  |
| Ccl20 | -0.171±0.384 | 0.980 | Tnfrsf12a | -0.099±0.152 | 0.778 | Eno2 | 0.059±0.096 | 0.817 | Itgb1bp2 | -0.132±0.147 | 0.568 |  |
| Ahr | 0.042±0.095 | 0.980 | Fst | -0.101±0.157 | 0.778 | Crim1 | 0.050±0.089 | 0.840 | Il1b | -0.113±0.128 | 0.568 |  |
| Tgfbr3 | 0.028±0.066 | 0.981 | Epcam | -0.044±0.084 | 0.876 | Vegfd | 0.042±0.077 | 0.840 | Tnfrsf11b | 0.184±0.221 | 0.604 |  |
| Epcam | 0.047±0.122 | 0.990 | Il10 | 0.125±0.246 | 0.885 | Il1a | 0.095±0.176 | 0.840 | Tgfb1 | -0.204±0.251 | 0.606 |  |
| Il10 | 0.067±0.180 | 0.990 | Il6 | 0.145±0.300 | 0.887 | Wisp1 | 0.050±0.092 | 0.840 | Apbb1ip | 0.161±0.204 | 0.615 |  |
| Ca13 | -0.053±0.149 | 0.990 | Erbb4 | -0.056±0.118 | 0.887 | Ahr | 0.045±0.095 | 0.887 | Epcam | -0.071±0.091 | 0.615 |  |
| Tnfrsf12a | 0.037±0.110 | 0.990 | Tnni3 | -0.348±0.749 | 0.887 | Vsig2 | -0.068±0.147 | 0.887 | Dctn2 | -0.251±0.327 | 0.615 |  |
| Il23r | -0.029±0.087 | 0.990 | Dctn2 | 0.135±0.299 | 0.887 | Snap29 | 0.089±0.198 | 0.887 | Dlk1 | -0.075±0.101 | 0.625 |  |
| Il6 | -0.041±0.124 | 0.990 | S100a4 | -0.064±0.152 | 0.901 | Casp3 | -0.084±0.204 | 0.905 | Ntf3 | 0.109±0.157 | 0.647 |  |
| Il5 | -0.064±0.231 | 0.990 | Casp3 | 0.077±0.201 | 0.928 | Acvrl1 | -0.062±0.154 | 0.905 | Clmp | -0.090±0.131 | 0.647 |  |
| Axin1 | 0.027±0.118 | 0.990 | Pak4 | 0.099±0.297 | 0.959 | Kitlg | -0.029±0.091 | 0.924 | Foxo1 | -0.066±0.096 | 0.647 |  |
| Snap29 | -0.042±0.199 | 0.990 | Itgb6 | 0.039±0.124 | 0.966 | Ccl3 | 0.049±0.156 | 0.924 | Ca13 | -0.158±0.237 | 0.652 |  |
| Casp3 | -0.042±0.205 | 0.990 | Ccl2 | -0.097±0.352 | 0.966 | Rgma | -0.024±0.076 | 0.924 | Il17f | -0.187±0.314 | 0.699 |  |
| Plin1 | -0.054±0.294 | 0.990 | Pdgfb | 0.082±0.300 | 0.966 | Dll1 | 0.025±0.081 | 0.924 | Axin1 | -0.087±0.150 | 0.699 |  |
| Pdgfb | 0.032±0.178 | 0.990 | Parp1 | 0.072±0.290 | 0.966 | Epo | -0.069±0.223 | 0.924 | Ddah1 | 0.176±0.311 | 0.699 |  |
| Itgb6 | -0.030±0.170 | 0.990 | Itgb1bp2 | 0.033±0.135 | 0.966 | Itgb1bp2 | 0.041±0.135 | 0.924 | Il1a | 0.136±0.241 | 0.699 |  |
| Hgf | 0.040±0.253 | 0.990 | Pla2g4a | 0.056±0.235 | 0.966 | Axin1 | -0.031±0.118 | 0.944 | Ccl2 | -0.188±0.385 | 0.745 |  |
| Pla2g4a | -0.025±0.166 | 0.990 | Apbb1ip | 0.039±0.187 | 0.966 | Notch3 | 0.015±0.065 | 0.944 | Il23r | -0.050±0.103 | 0.745 |  |
| Prdx5 | -0.029±0.197 | 0.990 | Hgf | -0.048±0.229 | 0.966 | Il5 | 0.053±0.230 | 0.944 | Mia | 0.047±0.113 | 0.791 |  |
| Cntn1 | 0.006±0.041 | 0.990 | Eno2 | 0.066±0.360 | 0.966 | Clstn2 | 0.027±0.117 | 0.944 | Eda2r | 0.084±0.216 | 0.806 |  |
| Tnfsf12 | 0.014±0.107 | 0.990 | Cntn1 | -0.015±0.096 | 0.966 | Gcg | 0.046±0.232 | 0.958 | Tnfrsf12a | -0.050±0.166 | 0.846 |  |
| Crim1 | 0.011±0.089 | 0.990 | Ca13 | 0.032±0.217 | 0.966 | Erbb4 | -0.016±0.094 | 0.958 | Fli1 | 0.061±0.204 | 0.846 |  |
| Gcg | -0.027±0.233 | 0.990 | Il23r | 0.013±0.094 | 0.966 | Lpl | -0.025±0.152 | 0.958 | Qdpr | -0.085±0.287 | 0.846 |  |
| Acvrl1 | -0.016±0.155 | 0.990 | Wfikkn2 | -0.015±0.123 | 0.966 | Tgfb1 | -0.035±0.233 | 0.958 | Ccl20 | 0.096±0.328 | 0.846 |  |
| Cxcl1 | 0.016±0.165 | 0.990 | Gcg | -0.032±0.280 | 0.966 | Il10 | -0.026±0.179 | 0.958 | Cant1 | -0.031±0.119 | 0.852 |  |
| Tgfb1 | -0.016±0.234 | 0.990 | Axin1 | 0.015±0.138 | 0.966 | Foxo1 | -0.009±0.079 | 0.967 | Kitlg | -0.031±0.121 | 0.852 |  |
| Gdnf | 0.004±0.086 | 0.990 | Riox2 | -0.031±0.297 | 0.966 | Plin1 | 0.028±0.292 | 0.967 | Wfikkn2 | -0.027±0.134 | 0.891 |  |
| S100a4 | -0.008±0.185 | 0.990 | Map2k6 | 0.018±0.201 | 0.966 | Ccl5 | -0.043±0.473 | 0.967 | Ahr | 0.023±0.133 | 0.901 |  |
| Fst | -0.008±0.211 | 0.990 | Ahr | -0.009±0.121 | 0.966 | Parp1 | 0.017±0.210 | 0.967 | Lgmn | -0.012±0.105 | 0.934 |  |
| Ppp1r2 | -0.005±0.168 | 0.990 | Fstl3 | -0.009±0.123 | 0.966 | Ghrl | -0.005±0.180 | 0.997 | Yes1 | -0.036±0.333 | 0.934 |  |
| Eda2r | -0.004±0.165 | 0.990 | Il5 | -0.020±0.384 | 0.968 | Il6 | -0.002±0.124 | 0.997 | Csf2 | -0.020±0.267 | 0.951 |  |
| Foxo1 | 0.001±0.080 | 0.992 | Foxo1 | 0.003±0.088 | 0.976 | Cntn1 | 0.000±0.041 | 1.000 | Il17a | -0.011±0.355 | 0.974 |  |
| *- FDR: false discovery rate, identified as increased (red) or decreased (blue) compared to isotype-mAb at each timepoint. Grayed cells indicate proteins common to both 2d and 5d. | | | | | | | | | | | | |
